# Design, synthesis, and biological evaluation of new 6,*N*^2^-diaryl-1,3,5-triazine-2,4-diamines as anticancer agents selectively targeting triple negative breast cancer cells†

**DOI:** 10.1039/d0ra04970k

**Published:** 2020-07-06

**Authors:** Ahmad Junaid, Felicia Phei Lin Lim, Edward R. T. Tiekink, Anton V. Dolzhenko

**Affiliations:** School of Pharmacy, Monash University Malaysia Jalan Lagoon Selatan Bandar Sunway Selangor Darul Ehsan 47500 Malaysia anton.dolzhenko@monash.edu; Research Centre for Crystalline Materials, School of Science and Technology, Sunway University 5 Jalan Universiti Bandar Sunway Selangor Darul Ehsan 47500 Malaysia; School of Pharmacy and Biomedical Sciences, Curtin Health Innovation Research Institute, Faculty of Health Sciences, Curtin University GPO Box U1987 Perth Western Australia 6845 Australia

## Abstract

New 6,*N*^2^-diaryl-1,3,5-triazine-2,4-diamines were designed using the 3D-QSAR model developed earlier. These compounds were prepared and their antiproliferative activity was evaluated against three breast cancer cell lines (MDA-MB231, SKBR-3 and MCF-7) and non-cancerous MCF-10A epithelial breast cells. The synthesized compounds demonstrated selective antiproliferative activity against triple negative MDA-MB231 breast cancer cells. The most active compound in the series inhibited MDA-MB231 breast cancer cell growth with a GI_50_ value of 1 nM. None of the tested compounds significantly affected the growth of the normal breast cells. The time-dependent cytotoxic effect, observed when cytotoxicity was assessed at different time intervals after the treatment, and morphological features, observed in the fluorescence microscopy and live cell imaging experiments, suggested apoptosis as the main pathway for the antiproliferative activity of these compounds against MDA-MB231 cells.

## Abstract 2

New highly potent and selective 6,*N*^2^-diaryl-1,3,5-triazine-2,4-diamines were designed and prepared using the 3D-QSAR model developed earlier.

## Graphical abstract

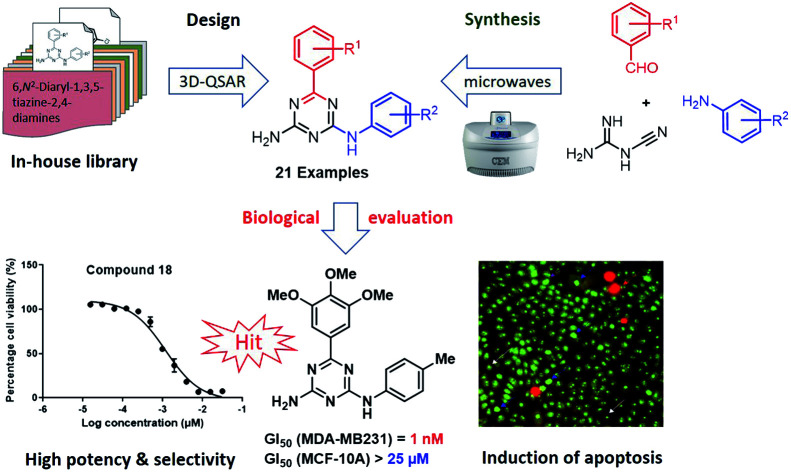

## 1. Introduction

Despite significant advancements in cancer therapy, cancer remains one of the diseases having the most negative impact on society. According to the World Health Organization, cancer was the second leading cause of patient lethality in 2018 causing almost 10 million deaths.^1^ Moreover, the cancer prevalence and mortality from cancer have been continuously growing worldwide, in both developing and developed countries. It was projected that from 14 million people suffering from cancer in 2012 the number of new cases per year will double by 2030.

Breast cancer had the highest incidence rates among all types of cancer in 2018 (46.3 per 100 000 females). In females, breast cancer is the most frequently diagnosed type of cancer and the prevalent cause of cancer deaths.^1^ Breast cancer is a rather heterogeneous form of cancer with cancer cells significantly varying in their properties and thus requiring different therapeutic approaches.^2^ On the basis of presence or absence of molecular markers, breast cancer is classified into 4 main subtypes: (1) human epidermal growth factor 2 (ERBB2) positive cancer with cells expressing ERBB2, (2) luminal A breast cancer with cells expressing estrogen or progesterone receptors but not ERBB2, (3) luminal B breast cancer with cells expressing hormone receptors and ERBB2 negative cells, and (4) triple negative breast cancer with cells lacking molecular markers used for this classification.

The current therapeutic options and agents under development for the treatment of different types of breast cancer vary significantly. The cancer cells overexpressing hormone receptors can be targeted by anti-estrogenic medicines, like tamoxifen, by aromatase inhibitors, like letrozole, or other medicines for endocrine therapy. To improve therapeutic outcome of the endocrine therapy, other agents with different mechanisms have been investigated: pan-class I phosphatidylinositol 3-kinase (PI3K) inhibitors (*e.g.* alpelisib and buparlisib),^3,4^ mammalian target of rapamycin (mTOR) inhibitors (*e.g.* everolimus),^5,6^ and cyclin-dependent kinase CDK4 and CDK6 inhibitors (*e.g.* palbociclib and ribociclib).^7–9^ For the treatment of ERBB2-positive breast cancer, PI3K and mTOR inhibitors are used together with ERBB2-targeted antibodies. Due to the absence of any targeted therapy for triple negative breast cancer, the general chemotherapy remains the main option available for the treatment of this most aggressive and mortal subtype of breast cancer. Typical medicines used against triple negative breast cancer include platinum drugs, taxanes, and anthracycline.^10^ A group of promising emerging medicines, poly(ADP-ribose) polymerase inhibitors (*e.g.* olaparib and talazoparib), have been identified as a more specific therapy for a subgroup of triple negative breast cancer with cells having a mutation of BRCA1/BRCA2 genes.^11^ New effective and selective anticancer agents are urgently needed for the safer and more effective treatment of triple negative breast cancer. The search for new potent compounds targeting breast cancer broadly covers various types of chemical structures.^12–14^

1,3,5-Triazine ring has been effectively used as a skeleton for the construction of new anticancer agents.^15^ Recently, we identified 6,*N*^2^-diaryl-1,3,5-triazine-2,4-diamines selectively targeting triple negative MDA-MB231 breast cancer cells.^16^ We also developed a 3D-QSAR model for the prediction of antiproliferative activity of this type of compounds against MDA-MB231 breast cancer cells. Herein, we are testing predictive power of this model for the design of new anticancer agents with the 6,*N*^2^-diaryl-1,3,5-triazine-2,4-diamine scaffold and continue our efforts on the development of highly potent and selective anticancer agents.

## 2. Results and discussion

### 2.1. QSAR-guided design of compounds

**Fig. 1:**
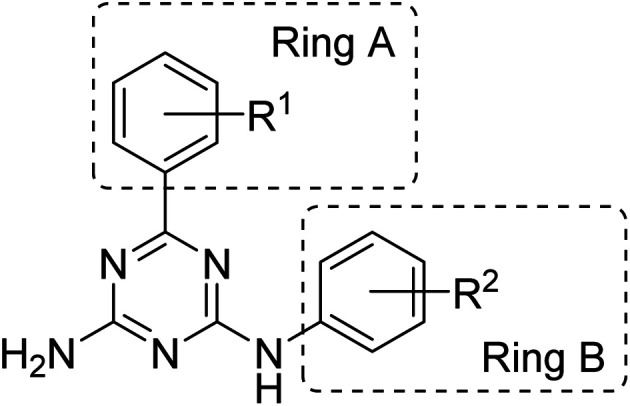
General structure of the designed compounds.

We previously reported synthesis of 6,*N*^2^-substituted 1,3,5-triazine-2,4-diamines (126 compounds) and their cytotoxic activity against breast cancer cell lines (MDA-MB231, SKBR-3 and MCF-7) and non-cancerous epithelial breast cells (MCF-10A).^16^ Some of the prepared compounds demonstrated selective activity against triple negative breast cancer cells (MDA-MB231). Twenty-five most active compounds were further evaluated and their GI_50_ values were estimated and used for the development of a 3D-QSAR model suitable for the design of new potent anticancer agents.^16^ The model is based on the activity of compounds with different substituents in phenyl rings A and B (Fig. 1).

The developed 3D-QSAR model indicated that bulky electron donating groups at the phenyl in the position 6 of the triazine, *i.e.* ring A would improve antiproliferative activity of compounds against MDA-MB231 cells. Based on this model, we designed a group of compounds bearing suitable functional groups at the phenyl rings A and B, with an expectation of higher activity against triple negative breast cancer, and applied the model to predict pGI_50_ values for these compounds (Table 1).

**Table 1:** 6,*N*^2^-Diaryl-1,3,5-triazine-2,4-diamines (1–21) and their antiproliferative activity against MDA-MB231 cells predicted using the QSAR model developed earlier^16^

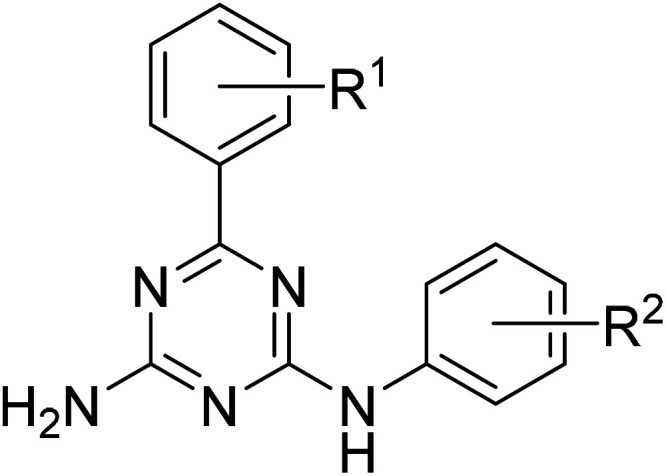			
Compound	R^1^	R^2^	Predicted pGI_50_
1	3-F	2-MeO	5.54
2	4-Cl	4-Me	5.51
3	4-CF_3_	2-Cl	5.22
4	4-CF_3_O	4-Cl	4.95
5	4-Me_2_N	2-Cl	5.79
6	4-Me	2-Cl	5.65
7	4-Me	4-Cl	5.51
8	4-MeO	H	6.58
9	4-MeO	4-Me	5.41
10	3,4,5-(MeO)_3_	H	5.58
11	3,4,5-(MeO)_3_	2-F	4.85
12	3,4,5-(MeO)_3_	2-Cl	5.10
13	3,4,5-(MeO)_3_	2-MeO	4.78
14	3,4,5-(MeO)_3_	3-Cl	4.62
15	3,4,5-(MeO)_3_	3-Me	4.49
16	3,4,5-(MeO)_3_	4-Cl	4.95
17	3,4,5-(MeO)_3_	4-Br	4.45
18	3,4,5-(MeO)_3_	4-Me	4.40
19	3,4,5-(MeO)_3_	4-MeO	4.79
20	3,4,5-(MeO)_3_	4-CF_3_O	4.49
21	3,4,5-(MeO)_3_	4-iPr	5.01

To test the earlier developed model, two main groups of compounds were selected for the synthesis. Number of substituents in each of the phenyl rings A and B for the first group of compounds (1–9) was limited to one functional group. The second group included 3,4,5-trimethoxyphenyl substituted compounds 10–21 to test effect of multiple substituents in ring A on the activity. Previously, we noticed that compounds with the R^1^ group in *meta*-position of ring A retained activity with a greater variety of substituents at another phenyl ring. Contrary, activity of compounds with *para*-position of R^1^ was very sensitive to the type and position of R^2^, disappearing when R^2^ was located in the *para*-position of ring B. Selecting compounds 10–21 with the preferred methoxy groups located in positions equivalent to the *para*- and both *meta*-positions of ring A, we intended to test which activity pattern they will follow. The predicted pGI_50_ values obtained from the 3D-QSAR model justified synthesis of the compounds.

### 2.2. Synthesis

Microwave irradiation has been widely used to facilitate synthesis of 1,3,5-triazines.^17^ Sometimes, microwave irradiation also changes outcome of reactions. The one-pot reaction of cyanoguanidine, benzaldehydes, and anilines in ethanol in the presence hydrochloric acid under conventional heating, followed by the treatment with aqueous sodium hydroxide (excess) was reported to produce 6,*N*^2^-diaryl-5,6-dihydro-1,3,5-triazine-2,4-diamines.^18^ However, a similar reaction under focused microwave irradiation resulted in the formation their fully aromatic analogues.^19^ This microwave-assisted methodology we applied for synthesis of new 6,*N*^2^-diaryl-1,3,5-triazine-2,4-diamines (1–21), which were designed using the 3D-QSAR model as describe above.

**Scheme 1:**
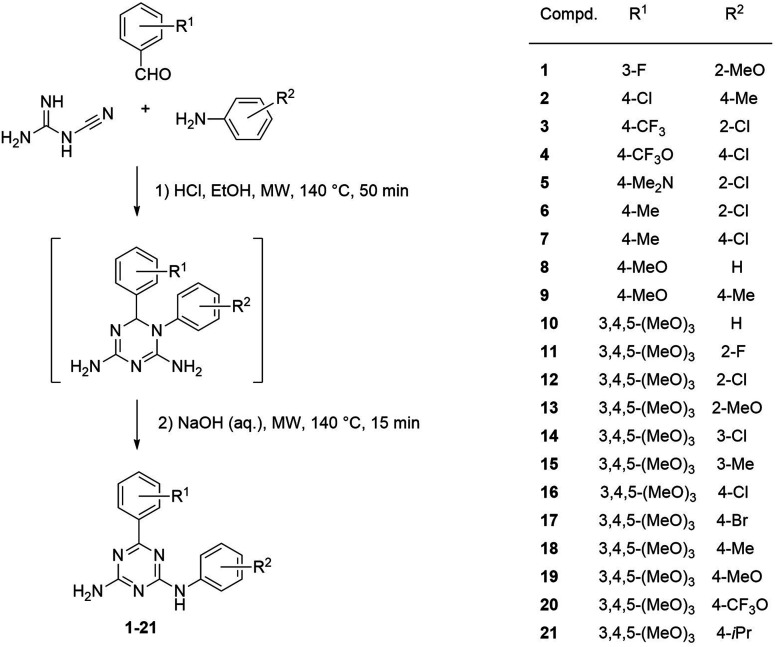
Synthesis of 6,*N*^2^-diaryl-1,3,5-triazine-2,4-diamines (1–21).

The reactions were performed in a one-pot manner with the three-component condensation of cyanoguanidine, benzaldehydes, and anilines at the first stage and the rearrangement accompanied with dehydrogenative aromatization at the second one (Scheme 1). The structure of the resulting 6,*N*^2^-diaryl-1,3,5-triazine-2,4-diamines (1–21) was confirmed by the NMR spectroscopic data and X-ray crystallographic study on one representative product, compound 20. The three diagnostic signals of the aromatic triazine ring quaternary carbon atoms appear in ^13^C NMR spectra of 1–21 in the region 164.4–170.2 ppm. In the ^1^H NMR spectra, the downfield shift of signals for protons in the *ortho*-positions of the phenyl ring directly attached to the 1,3,5-triazine ring should be attributed to the anisotropic effect of the coplanar triazine π-electron system.

**Fig. 2:**
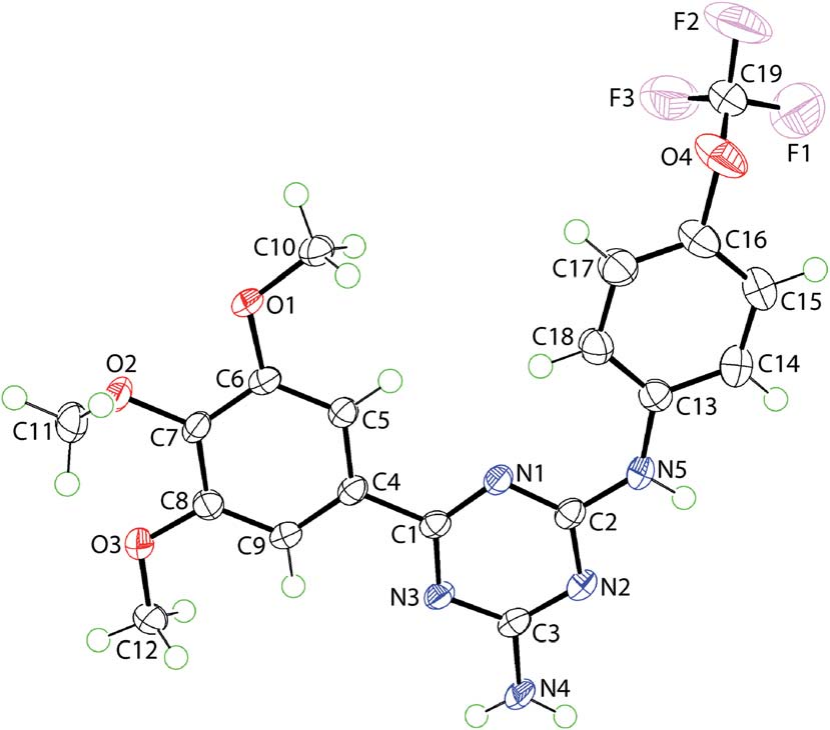
Molecular structure of 20, showing atom labelling scheme and anisotropic displacement parameters at the 70% probability level.

X-ray crystallography of 20 (Fig. 2) showed that to a first approximation the molecule is planar and has the shape of the letter U as both appended aromatic rings are orientated to the same side of the molecule. Within the triazine ring, the near equivalence of the C–N bond lengths is indicative of substantial delocalisation of π-electron density over the ring. Details of crystallographic analysis are available in ESI.†

### 2.3. Biological evaluation

#### 2.3.1. Cytotoxicity evaluation

The prepared compounds 1–21 were tested against three breast tumor cell lines: hormone (estrogen and progesterone) negative MDA-MB231 and hormone positive SKBR-3 and MCF-7. The initial screening of 6,*N*^2^-diaryl-1,3,5-triazine-2,4-diamines (1–21) was performed at one point concentration (10 μM) for preliminary assessment of their antiproliferative potential (Table 2). Percentage cell viability was calculated 72 h after treatment with compounds. In general, triple negative breast cancer cells (MDA-MB231) were more responsive than hormone positive breast cancer cells (SKBR-3 and MCF-7) to the treatment with the compounds. These results are similar to the trend observed earlier for their structural analogues.^16^

**Table 2:** Preliminary cytotoxic screening of 6,*N*^2^-1,3,5-triazine-2,4-diamines (1–21) on breast cancer cell lines at 10 μM

Compound	R^1^	R^2^	Cell viability^a^ (%)		
			MDA-MB231	SKBR-3	MCF-7
1	3-F	2-MeO	49	84	88
2	4-Cl	4-Me	29	85	100
3	4-CF_3_	2-Cl	49	83	96
4	4-CF_3_O	4-Cl	34	86	95
5	4-Me_2_N	2-Cl	28	55	86
6	4-Me	2-Cl	36	80	51
7	4-Me	4-Cl	51	76	95
8	4-MeO	H	28	67	85
9	4-MeO	4-Me	32	89	99
10	3,4,5-(MeO)_3_	H	14	37	62
11	3,4,5-(MeO)_3_	2-F	34	73	98
12	3,4,5-(MeO)_3_	2-Cl	42	80	100
13	3,4,5-(MeO)_3_	2-MeO	16	57	100
14	3,4,5-(MeO)_3_	3-Cl	40	58	96
15	3,4,5-(MeO)_3_	3-Me	14	43	86
16	3,4,5-(MeO)_3_	4-Cl	20	48	52
17	3,4,5-(MeO)_3_	4-Br	24	43	48
18	3,4,5-(MeO)_3_	4-Me	14	37	62
19	3,4,5-(MeO)_3_	4-MeO	20	48	52
20	3,4,5-(MeO)_3_	4-CF_3_O	20	45	51
21	3,4,5-(MeO)_3_	4-iPr	9	42	49

a MTT method, cells incubated with corresponding compounds (10 μM) for 72 h. Values are mean of three independent experiments.

Since all compounds 1–21 demonstrated significant antiproliferative activity against MDA-MB231 cells at the screening concentration, they were further tested at concentration ranging from 0.00002 μM to 20 μM to estimate their 50% growth inhibitory concentrations (GI_50_) against breast cancer cells (Table 3). Nilotinib and methotrexate were used as positive controls. For compounds active at the screening concentration against SKBR-3 and MCF-7 cells, concentration-dependent response was also evaluated and the corresponding GI_50_ values were estimated.

**Table 3:** Cytotoxicity^a^ of 6,*N*^2^-diaryl-1,3,5-triazine-2,4-diamines (1–21)

Compound	R^1^	R^2^	GI_50_ ± SD^b^ (μM)			
			MDA-MB231	SKBR-3	MCF-7	MCF-10A
1	3-F	2-MeO	17.3 ± 0.6	>20	>20	>20
2	4-Cl	4-Me	13.8 ± 1.9	>20	>20	>20
3	4-CF_3_	2-Cl	13.7 ± 0.6	17.7 ± 1.4	>20	>20
4	4-CF_3_O	4-Cl	16.7 ± 1.2	>20	>20	>20
5	4-Me_2_N	2-Cl	0.1 ± 0.001	0.4 ± 0.04	>20	>20
6	4-Me	2-Cl	3.8 ± 0.4	>20	10.7 ± 1.0	>20
7	4-Me	4-Cl	9.6 ± 0.2	>20	>20	>20
8	4-MeO	H	8.4 ± 0.3	19.6 ± 0.9	14.2 ± 1.7	>20
9	4-MeO	4-Me	6.1 ± 0.6	>20	>20	>20
10	3,4,5-(MeO)_3_	H	9.7 ± 0.6	17.2 ± 0.4	>20	>20
11	3,4,5-(MeO)_3_	2-F	7.9 ± 0.5	>20	>20	>20
12	3,4,5-(MeO)_3_	2-Cl	11.3 ± 1.1	>20	>20	>20
13	3,4,5-(MeO)_3_	2-MeO	2.1 ± 0.2	14.0 ± 1.6	>20	>20
14	3,4,5-(MeO)_3_	3-Cl	9.1 ± 1.1	16.7 ± 1.4	>20	>20
15	3,4,5-(MeO)_3_	3-Me	2.2 ± 0.2	6.0 ± 0.1	>20	>20
16	3,4,5-(MeO)_3_	4-Cl	0.007 ± 0.00001	0.3 ± 0.04	12.5 ± 0.2	>20
17	3,4,5-(MeO)_3_	4-Br	0.008 ± 0.0005	0.17 ± 0.01	>20	>20
18	3,4,5-(MeO)_3_	4-Me	0.001 ± 0.00001	0.21 ± 0.01	>20	>20
19	3,4,5-(MeO)_3_	4-MeO	0.01 ± 0.001	0.27 ± 0.02	>20	>20
20	3,4,5-(MeO)_3_	4-CF_3_O	1.5 ± 0.1	5.0 ± 0.35	>20	>20
21	3,4,5-(MeO)_3_	4-iPr	0.04 ± 0.002	1.1 ± 0.05	10.7 ± 1.1	>20
Methotrexate^c^			0.01 ± 0.001	ND	5.8 ± 0.5	ND
Nilotinib^c^			0.04 ± 0.001	9.60 ± 0.5	ND	ND

a MTT method, cells incubated with compounds for 72 h, experiments performed in triplicates.

b Standard deviation of mean values.

c Positive control.

Compounds 1–21 were also tested against MCF-10A normal breast cells to evaluate their selectivity towards cancer cells. None of the compounds showed significant inhibition of the normal breast cell growth at the compound concentration of 20 μM.

The prepared 6,*N*^2^-diaryl-1,3,5-triazine-2,4-diamines 1–21 possessed specific cytotoxicity against triple negative MDA-MB231 breast cancer cells with GI_50_ values ranging widely. However, the most intriguing results were obtained for compounds 10–21 with the 3,4,5-trimethoxyphenyl moiety as the ring A. This substitution was exceptionally beneficial for the anticancer activity, particularly in a combination with the *para*-substitution at the phenyl ring B. Changing location of the substituents to *ortho*- or *meta*-position in the phenylamino moiety dramatically decreased potency of compounds. The GI_50_ values for these subgroups have a 2–3 order difference. For example, relocation of the methoxy group from the *ortho*- to *para*-position of the ring B resulted in a 200-fold increase in the antiproliferative activity (13*vs.*19). Even greater improvement in the activity was achieved when methyl or chloro substituents changed their location at the ring B from *meta*- to *para*-position leading to compounds 1300–2000-fold more potent than their regioisomers (14*vs.*16, 15*vs.*18). At the same time, it appeared that for the trimethoxyphenyl-substituted series (10–21) an increase in size of the R^2^ group in *para*-position from the most potent compound with a methyl group (18) decreased the activity. Nevertheless, most of the triazines combining trimethoxyphenyl as the ring A and *para*-substituted phenylamino moieties as the ring B possessed activity comparable or higher than that of reference drugs methotrexate and nilotinib. These compounds also demonstrated good antiproliferative activity against SKBR-3 cells. The most active 6,*N*^2^-diaryl-1,3,5-triazine-2,4-diamine identified in the series was compound 18, which was 10-fold more active than methotrexate and 40-fold more potent than nilotinib against MDA-MB231 breast cancer cells. This compound (18) and its analogue 16, with the chloro substituent instead of the *para*-methyl group in the ring B, were selected for further experiments to better understand processes underlying antiproliferative effects of these compounds.

To assess predictive power of the earlier developed 3D-QSAR model, we compared experimental and predicted pGI_50_ values, calculated using the 3D-QSAR model (Table 4). The residual error values for the first series of compounds (1–9) were rather acceptable *viz.* without extreme differences between the experimental and predicted values. However, a large discrepancy between the predicted and experimental values was observed for many trimethoxyphenyl-substituted compounds. These compounds, especially those with the R^2^ group in *para*-position of the ring B (16–21), appeared to be much more potent than it was predicted by the model. These findings indicated a limitation of the earlier prepared 3D-QSAR model,^16^ which seemed to be valid for compounds with monosubstituted phenyl rings and should be used with a caution for more complex structures.

**Table 4:** Antiproliferative activities obtained experimentally and predicted for 6,*N*^2^-diaryl-1,3,5-triazine-2,4-diamines (1–21) by the 3D-QSAR model^a^

Compound	R^1^	R^2^	Experimental pGI_50_^b^	Predicted pGI_50_^c^	Residual error^d^
1	3-F	2-MeO	4.76	5.54	−0.78
2	4-Cl	4-Me	4.86	5.51	−0.64
3	4-CF_3_	2-Cl	4.86	5.22	−0.35
4	4-CF_3_O	4-Cl	4.78	4.95	−0.18
5	4-Me_2_N	2-Cl	7.00	5.79	1.21
6	4-Me	2-Cl	5.42	5.65	−0.23
7	4-Me	4-Cl	5.02	5.51	−0.49
8	4-MeO	H	5.08	6.58	−1.50
9	4-MeO	4-Me	5.21	5.41	−0.19
10	3,4,5-(MeO)_3_	H	5.01	5.58	−0.56
11	3,4,5-(MeO)_3_	2-F	5.10	4.85	0.25
12	3,4,5-(MeO)_3_	2-Cl	4.95	5.10	−0.15
13	3,4,5-(MeO)_3_	2-MeO	5.69	4.78	0.91
14	3,4,5-(MeO)_3_	3-Cl	5.04	4.62	0.42
15	3,4,5-(MeO)_3_	3-Me	5.66	4.49	1.17
16	3,4,5-(MeO)_3_	4-Cl	8.15	4.95	3.20
17	3,4,5-(MeO)_3_	4-Br	8.10	4.45	3.65
18	3,4,5-(MeO)_3_	4-Me	8.70	4.40	4.30
19	3,4,5-(MeO)_3_	4-MeO	8.00	4.79	3.21
20	3,4,5-(MeO)_3_	4-CF_3_O	5.83	4.49	1.34
21	3,4,5-(MeO)_3_	4-iPr	7.40	5.01	2.39

a QSAR model reported earlier.^16^

b Experimental pGI_50_ calculated as pGI_50_ = −log 10 × GI_50_.

c pGI_50_ values predicted by the QSAR model.

d Difference between the predicted and experimental pGI_50_ values.

#### 2.3.2. Time-dependent cytotoxicity

To further evaluate cytotoxicity of the prepared compound against cancer cells, time-dependent cell viability experiments were carried out with the selected most active compounds 16 and 18 using MDA-MB231 breast cancer cell line. The MDA-MB231 cell viability was assessed after the exposure of the cells to compounds 16 or 18 for 12, 24, 48, and 72 h at concentrations ranging from 0.2 nM to 125 nM. The GI_50_ values were estimated when treatment with the highest concentration (125 nM) of tested compounds resulted in more than 80% of cell growth inhibition (Table 5).

**Table 5:** Time-dependent cytotoxic effect^a^ of the most active compounds (16 and 18) against MDA-MB231 breast cancer cell line

Compound	GI_50_ ± SD (nM) or growth inhibition at 125 nM			
	12 h	24 h	48 h	72 h
16	17%^b^	61%^b^	75%^b^	7 ± 0.6
18	15%^b^	5 ± 0.1	4 ± 0.3	1 ± 0.02

a MTT method, values are the mean ± SD, all experiments performed at least three times.

b Percentage cell growth inhibition at 125 nM concentration of test compounds.

Compound 18 possessed higher antiproliferative activity than 16 against MDA-MB231 cells for all duration of observations. For both compounds, the cytotoxic effect developed gradually and no significant inhibition of the cell growth was detected 12 h after the treatment. However, compound 18 started showing activity in nanomolar concentrations (GI_50_ = 5 nM) at 24 h with an increase in the potency over the following 24 h (GI_50_ = 4 nM) and even more after the total exposure for 72 h (GI_50_ = 1 nM). A similar time-dependent pattern was observed for compound 16.

These results suggest that the antiproliferative effect of compounds 16 and 18 develop gradually and without an immediate toxic effect on the cells. A negligible cytotoxicity 12 h after the treatment suggests that the compounds are less likely to cause cell necrosis and probably induce apoptosis. To further test this assumption, we performed fluorescent microscopy experiments assessing effects of compounds 16 and 18 on the morphology of MDA-MB231 breast cancer cell.

#### 2.3.3. Acridine orange and propidium iodide double staining experiments

After the determination of cytotoxicity by the MTT assay, morphological changes of MDA-MB231 cells treated with most active compounds 16 and 18 were studied using fluorescence microscopy. The acridine orange (AO) and propidium iodide (PI) double staining method was used to determine morphological features of apoptotic cells (chromatin condensation, cell blebbing and apoptotic bodies). AO emits green light by intercalating the DNA of the live and dead cells, while PI emits red fluorescence by intercalating the DNA of dead cells only.^20^

**Fig. 3:**
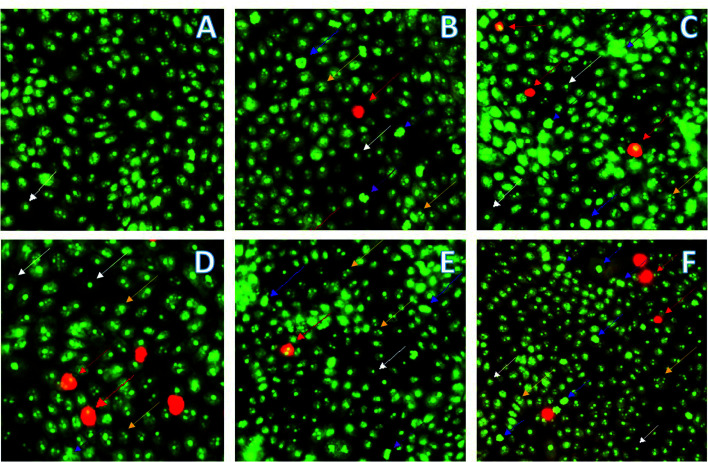
AO/PI double staining of MDA-MB231 cells with signs of apoptosis 24 h after the treatment with compounds. (A) Cells treated with a vehicle, 1% DMSO, negative control; (B) cells treated with compound 16 (125 nM); (C) cells treated with compound 16 with (250 nM); (D) cells treated with methotrexate (10 nM), positive control; (E) cells treated with compound 18 IC_20_ (2.5 nM); (F) cells treated with compound 18 IC_50_ (5 nM). Images were taken with a fluorescence microscope at 400×. White arrow points to live cells, red arrow shows apoptotic cells, blue arrow points to chromatin condensation, purple arrow shows cell blebbing and yellow arrow indicates multi-nucleated cells.

MDA-MB231 cells were treated with compounds 16 and 18 and incubated for 24 h prior to the observation of changes in cell morphology. The selected representative images of fluorescence microscopy are presented in Fig. 3: live cells emit green color (white arrow) because of AO intercalation with DNA and apoptotic cells appear reddish-orange (red arrow) by intercalating PI to the DNA because of altered membrane permeability. Mid-stage apoptosis is evident by the presence of cells with nuclear chromatic condensation (blue arrow), cell blebbing (purple arrow), and multi-nucleated cells (yellow arrow).

#### 2.3.4. Live cell imaging

To visualize morphological changes in the cells in real time, live cell imaging of MDA-MB231 cells treated with compound 18 was carried out. The cells were stained with AO and PI and treated with 18 (10 μM). The pictures were taken after every 10 minutes for 4 h and intercalated into video (see ESI†). The video clearly shows the morphological changes of the cells initiated by 18 at different times, like formation of multinucleation, chromatin condensation, cell blebbing and apoptotic bodies. The death of the breast cancer cells (MDA-MB231) treated with compound 18 was also evident from turning of live cells (green color) to dead cells (red color). These observations suggest that compound 18 realizes its cytotoxic activity by inducing apoptosis in MDA-MB231 cells.

### 2.4. Prediction of ADME properties

In the design of biologically active agents, optimization of lead compounds and selection of drug candidates, *in silico* evaluation of absorption, distribution, metabolism and elimination (ADME) of compounds has become a common practice.^21^ QikProp (version 4.3) module of the Schrödinger software was used to predict the molecular properties influencing critical pharmacokinetic parameters of compounds 1–21 (Table 6). Parameters like octanol/water partition coefficient (*QP* log *P*, o/w) and aqueous solubility (*QP* log *S*) are important for the prediction of drug absorption, transport and distribution in the body. These parameters calculated for 1–12 have values similar to those, which are typical for commonly used drugs. Steric and molecular surface descriptors *i.e.*, total solvent accessible area (SASA) and its hydrophobic (FOSA) and hydrophilic (FISA) components were also calculated and found to be within the 95% range of values for known drugs. Lipinski's rule of five has been often used as a first filter for the prediction the drug-like properties of compounds.^22^ None of the prepared compounds violate Lipinski's rule of five. The complete absorption and absence of effects on CNS were predicted for compounds 1–21. Overall, all evaluated compounds were predicted to possess ADME properties favorable for potential agents targeting breast cancer cells. More detailed ADME profile for the compounds predicted by QikProp module is available in ESI.†

**Table 6:** Selected ADME properties of 6,*N*^2^-diaryl-1,3,5-triaizne-2,4-diamines (1–21)^a^

Compound	MW^b^	SASA^c^	Donor	Accpt	*QP* log *P*	*QPP*	#Metab^h^	Percent human oral absorption^i^
			HB^d^	HB^e^	o/w^f^	Caco^g^		
1	311.32	663.94	3	4.75	3.50	849.24	2	100
2	311.77	659.67	3	4.00	3.86	784.19	1	100
3	365.75	642.85	3	4.00	4.06	922.41	1	100
4	381.74	696.37	3	4.00	4.56	800.65	1	100
5	340.81	709.92	3	5.00	4.03	903.51	2	100
6	311.77	664.90	3	4.00	3.89	924.51	2	100
7	311.77	668.19	3	4.00	3.87	783.98	1	100
8	293.33	644.11	3	4.75	3.17	789.12	2	100
9	307.35	666.66	3	4.75	3.44	787.51	2	100
10	353.38	735.08	3	6.25	3.43	825.33	4	100
11	371.37	742.37	3	6.25	3.67	887.43	4	100
12	387.83	755.90	3	6.25	3.93	969.42	4	100
13	383.41	766.44	3	7.00	3.34	893.13	5	100
14	387.83	759.21	3	6.25	3.91	822.03	4	100
15	367.41	766.57	3	6.25	3.72	824.23	5	100
16	387.83	759.20	3	6.25	3.91	822.03	3	100
17	432.28	764.15	3	6.25	3.99	821.52	3	100
18	367.41	758.05	3	6.25	3.71	822.74	4	100
19	383.41	750.27	3	7.00	3.44	821.39	4	100
20	437.38	803.46	3	6.25	4.78	802.60	4	100
21	395.46	800.44	3	6.25	4.27	818.46	4	100

a Calculated using QikProp 4.3 module of the Schrödinger software.

b Molecular weight.

c Total solvent accessible surface area in Å^2^ using a probe with a 1.4 Å radius, range 95% of drugs (300.0–1000.0).

d Estimated number of hydrogen bonds that would be donated by the solute to water molecules in an aqueous solution, range 95% of drugs (0.0–6.0).

e Estimated number of hydrogen bonds that would be accepted by the solute from water molecules in an aqueous solution, range 95% of drugs (2.0–20.0).

f Predicted log of the octanol/water partition coefficient, range 95% of drugs (−2–6.5).

g Caco-2 cell permeability in nm s^−1^, range 95% of drugs (<25 poor, >500 great). Caco-2 cells are a model for the gut blood barrier, non-active transport.

h Number of likely metabolic reactions; range 95% of drugs (1–8).

i Human oral absorption predicted on the basis of a quantitative multiple linear regression model. 0 to 100% scale (>85% high).

## 3. Conclusions

We synthesized a library of novel 6,*N*^2^-diaryl-1,3,5-triazine-2,4-diamines designed using the 3D-QSAR data from the previous report.^16^ Their antiproliferative activity was evaluated against three breast cancer cell lines and it was found that triple negative breast cancer cells (MDA-MB231) were significantly more sensitive to the treatment with the prepared compounds. Some 6,*N*^2^-diaryl-1,3,5-triazine-2,4-diamines demonstrated good antiproliferative activity against SKBR-3 cells, but MCF-7 cells were generally resistant to the treatments with these compounds.

Some of the synthesized compounds demonstrated even greater activity against MDA-MB231 cells than it was predicted by the 3D-QSAR model. The 3D-QSAR model limitation might originate from multiple targets responsible for the activity of 6,*N*^2^-diaryl-1,3,5-triazine-2,4-diamines and hence requires further investigations. The discrepancy between the predicted values and the experimental data was particularly evident for *N*^2^-aryl-6-(3,4,5-trimethoxyphenyl)-1,3,5-triazine-2,4-diamines 16–21 possessing *para*-substituted phenyl ring B. The most active compound in the series also belongs to this group: compound 18 inhibited triple negative MDA-MB231 breast cancer cell growth with GI_50_ value of 1 nM. Importantly, the prepared compounds demonstrated no cytotoxicity towards non-cancerous MCF-10A breast cells. The cytotoxic evaluation at different time intervals for the most active compounds 16 and 18 showed that these compounds possessed a concentration- and time-dependent cytotoxic effect on MDA-MB231 breast cancer cells. Morphological features observed by the fluorescent microscopy and live cell imaging after the AO/PI double staining suggested that the tested compounds induced apoptosis in MDA-MB231 cells. All compounds, including 18, were predicted to have ADME profiles favorable for potential antiproliferative agents targeting breast cancer.

## 4. Experimental

### 4.1. General

Melting points (uncorrected) were determined using a Stuart™ SMP40 automatic melting point apparatus. ^1^H and ^13^C NMR spectra were recorded on a Bruker Fourier NMR spectrometer (300 MHz) using DMSO-*d*_6_ as a solvent and TMS as an internal reference. Microwave-assisted reactions were carried out in the closed vessel focused single mode using a Discover SP microwave synthesizer (CEM, USA) monitoring reaction temperature by the equipped IR sensor.

### 4.2. General method for the synthesis of 6,*N*^2^-diaryl-1,3,5-triazine-2,4-diamines (1–21)

The microwave irradiation parameters optimized earlier^19^ for the synthesis of 6,*N*^2^-diaryl-1,3,5-triazine-2,4-diamines were applied for the preparation of 1–21. To a solution of cyanoguanidine (0.21 g, 2.5 mmol), a substituted benzaldehyde (2.5 mmol), and an aniline (2.5 mmol) in EtOH (2 mL) in a 10 mL seamless pressure vial, conc. HCl (0.21 mL, 2.5 mmol) was added. The reaction mixture was heated at 140 °C for 50 min by irradiation in the Discover SP (CEM) microwave reactor operating at maximal microwave power up to 150 W. Then, an aq. solution of NaOH (5 N, 1 mL) was added to the reaction mixture and heating was continued for another 15 min at 140 °C. After cooling, the precipitated product was filtered, washed with water and recrystallized from suitable solvents (EtOH, aq. EtOH, or MeCN) specified below. Yields of products 1–21 are reported as overall isolated yields for the one-pot procedure.

#### 4.2.1. 6-(3-Fluorophenyl)-*N*^2^-(2-methoxyphenyl)-1,3,5-triazine-2,4-diamine (1)

Yield 33%. Mp 140–142 °C (EtOH/water). ^1^H NMR (300 MHz, DMSO-*d*_6_): *δ* 3.87 (OCH_3_), 6.95–7.10 (3H, m, H-3′′, H-4′′ and H-5′′), 7.22 (2H, brs, NH_2_), 7.40 (1H, dddd, ^4^*J*_HH_ = 0.8 Hz, ^4^*J*_HH_ = 2.6 Hz, ^3^*J*_HH_ = 8.5 Hz, ^3^*J*_HF_ = 8.4 Hz, H-4′), 7.56 (1H, ddd, ^4^*J*_HF_ = 6.0 Hz, ^3^*J*_HH_ = 8.0 Hz, ^3^*J*_HH_ = 8.0 Hz, H-5′), 8.00 (1H, ddd, ^4^*J*_HH_ = 1.3 Hz, ^4^*J*_HH_ = 2.6 Hz, ^3^*J*_HF_ = 10.6 Hz, H-2′), 8.12 (1H, s, NH), 8.13–8.17 (2H, m, H-6′′ and H-6′); ^13^C NMR (75 MHz, DMSO-*d*_6_): *δ* 55.7 (OCH_3_), 111.0 (C-3′′), 114.0 (d, ^2^*J*_CF_ = 23.1 Hz, C-2′), 118.2 (d, ^2^*J*_CF_ = 21.8 Hz, C-4′), 120.2 (C-5′′), 122.3 (C-6′′), 123.7 (d, ^4^*J*_CF_ = 2.2 Hz, C-6′), 123.8 (C-4′′), 127.6 (C-1′′), 130.3 (d, ^3^*J*_CF_ = 8.2 Hz, C-5′), 139.2 (d, ^3^*J*_CF_ = 7.5 Hz, C-1′), 149.9 (C-2′′), 162.1 (d, ^1^*J*_CF_ = 242.9 Hz, C-3′), 164.6 (C-2), 167.2 (C-4), 169.0 (d, ^4^*J*_CF_ = 3.0 Hz, C-6). Anal. calcd for C_16_H_14_FN_5_O: C, 61.73; H, 4.53; N, 22.50. Found: C, 61.65; H, 4.77; N, 22.26.

#### 4.2.2. 6-(4-Chlorophenyl)-*N*^2^-(4-methylphenyl)-1,3,5-triazine-2,4-diamine (2)

Yield 45%. Mp 179–181 °C (MeCN). ^1^H NMR (300 MHz, DMSO-*d*_6_): *δ* 2.27 (3H, s, CH_3_), 7.11 (2H, d, *J* = 8.3 Hz, H-3′′ and H-5′′), 7.12 (2H, brs, NH_2_), 7.58 (2H, d, *J* = 8.6 Hz, H-3′ and H-5′), 7.68 (2H, d, *J* = 8.3 Hz, H-2′′ and H-6′′), 8.29 (2H, d, *J* = 8.6 Hz, H-2′ and H-6′), 9.46 (1H, s, NH); ^13^C NMR (75 MHz, DMSO-*d*_6_): *δ* 20.3 (CH_3_), 120.1 (C-2′′ and C-6′′), 128.4 (C-3′ and C-5′), 128.8 (C-3′′ and C-5′′), 129.4 (C-2′ and C-6′), 130.9 (C-1′′), 135.7 (C-1′), 136.1 (C-4′), 137.2 (C-4′′), 164.5 (C-4), 167.1 (C-6), 169.1 (C-2). Anal. calcd for C_16_H_14_ClN_5_: C, 61.64; H, 4.53; N, 22.46. Found: C, 61.54; H, 4.50; N, 22.29.

#### 4.2.3. *N* ^2^-(2-Chlorophenyl)-6-(4-(trifluoromethyl)phenyl)-1,3,5-triazine-2,4-diamine (3)

Yield 38%. Mp 165–167 °C (EtOH). ^1^H NMR (300 MHz, DMSO-*d*_6_): *δ* 7.23 (2H, brs, NH_2_), 7.23 (1H, ddd, *J* = 1.6 Hz, *J* = 7.7 Hz, *J* = 7.7 Hz, H-4′′), 7.38 (1H, ddd, *J* = 1.3 Hz, *J* = 7.7 Hz, *J* = 7.7 Hz, H-5′′), 7.53 (1H, dd, *J* = 1.4 Hz, *J* = 8.0 Hz, H-3′′), 7.80 (1H, dd, *J* = 1.6 Hz, *J* = 8.0 Hz, H-6′′), 7.88 (2H, d, *J* = 8.3 Hz, H-3′ and H-5′), 8.45 (2H, d, *J* = 8.1 Hz, H-2′ and H-6′), 8.97 (1H, s, NH); ^13^C NMR (75 MHz, DMSO-*d*_6_): *δ* 120.5 (q, ^1^*J*_CF_ = 273.6 Hz, CF_3_), 125.2 (q, ^3^*J*_CF_ = 3.5 Hz, C-3′ and C-5′), 126.2 (C-6′′), 127.3 (C-2′′), 127.5 (C-5′′), 128.4 (C-2′ and C-6′), 128.5 (C-4′′), 129.4 (C-3′′), 131.1 (q, ^2^*J*_CF_ = 31.8, C-4′), 135.6 (C-1′′), 140.5 (C-1′), 165.3 (C-4), 167.3 (C-6), 169.0 (C-2). Anal. calcd for C_16_H_11_ClF_3_N_5_: C, 52.54; H, 3.03; N, 19.15. Found: C, 52.33; H, 2.95; N, 18.98.

#### 4.2.4. *N* ^2^-(4-Chlorophenyl)-6-(4-(trifluoromethoxy)phenyl)-1,3,5-triazine-2,4-diamine (4)

Yield 55%. Mp 201–203 °C (EtOH). ^1^H NMR (300 MHz, DMSO-*d*_6_): *δ* 7.27 (2H, brs, NH_2_), 7.36 (2H, d, *J* = 8.9 Hz, H-3′′ and H-5′′), 7.51 (2H, dd, ^5^*J*_HF_ = 0.9 Hz, ^3^*J*_HH_ = 9.0 Hz, H-3′ and H-5′), 7.89 (2H, d, *J* = 8.9 Hz, H-2′′ and H-6′′), 8.43 (2H, d, *J* = 8.9 Hz, H-2′ and H-6′), 9.75 (1H, s, NH); ^13^C NMR (75 MHz, DMSO-*d*_6_): *δ* 120.0 (q, ^1^*J*_CF_ = 257.0 Hz, OCF_3_), 120.5 (C-3′ and C-5′), 121.4 (C-2′′ and C-6′′), 125.7 (C-1′′), 128.2 (C-3′′ and C-5′′), 129.9 (C-2′ and C-6′), 135.7 (C-1′), 138.8 (C-4′′), 150.6 (q, ^3^*J*_CF_ = 1.7 Hz, C-4′), 164.5 (C-4), 167.1 (C-6), 169.1 (C-2). Anal. calcd for C_16_H_11_ClF_3_N_5_O: C, 50.34; H, 2.90; N, 18.35. Found: C, 50.22; H, 3.02; N, 18.26.

#### 4.2.5. *N* ^2^-(2-Chlorophenyl)-6-(4-(dimethylamino)phenyl)-1,3,5-triazine-2,4-diamine (5)

Yield 15%. Mp 191–193 °C (EtOH). ^1^H NMR (300 MHz, DMSO-*d*_6_): *δ* 2.99 (6H, s, N(CH_3_)_2_), 6.74 (2H, d, *J* = 9.1 Hz, H-3′ and H-5′), 6.89 (2H, brs, NH_2_), 7.16 (1H, ddd, *J* = 1.6 Hz, *J* = 7.7 Hz, *J* = 7.7 Hz, H-4′′), 7.36 (1H, ddd, *J* = 1.2 Hz, *J* = 7.8 Hz, *J* = 7.8 Hz, H-5′′), 7.50 (1H, dd, *J* = 1.5 Hz, *J* = 8.0 Hz, H-3′′), 7.96 (1H, dd, *J* = 1.5 Hz, *J* = 8.1 Hz, H-6′′), 8.13 (2H, d, *J* = 9.2 Hz, H-2′ and H-6′), 8.43 (1H, s, NH); ^13^C NMR (75 MHz, DMSO-*d*_6_): *δ* 39.6 (N(CH_3_)_2_), 110.9 (C-3′ and C-5′), 123.2 (C-1′), 125.2 (C-6′′), 126.2 (C-2′′), 126.9 (C-4′′), 127.2 (C-5′′), 129.2 (C-2′ and C-6′), 129.2 (C-3′′), 135.9 (C-1′′), 152.5 (C-4′), 164.8 (C-4), 167.0 (C-6), 170.2 (C-2). Anal. calcd for C_17_H_17_ClN_6_: C, 59.91; H, 5.03; N, 24.66. Found: C, 59.79; H, 4.96; N, 24.47.

#### 4.2.6. *N* ^2^-(2-Chlorophenyl)-6-(4-methylphenyl)-1,3,5-triazine-2,4-diamine (6)

Yield 40%. Mp 146–148 °C (EtOH). ^1^H NMR (300 MHz, DMSO-*d*_6_): *δ* 2.37 (3H, s, CH_3_), 7.07 (2H, brs, NH_2_), 7.20 (1H, ddd, *J* = 1.6 Hz, *J* = 7.8 Hz, *J* = 7.6 Hz, H-4′′), 7.29 (2H, d, *J* = 8.0 Hz, H-3′ and H-5′), 7.37 (1H, ddd, *J* = 1.4 Hz, *J* = 7.7 Hz, *J* = 7.9 Hz, H-5′′), 7.52 (1H, dd, *J* = 1.4 Hz, *J* = 8.0 Hz, H-3′′), 7.87 (1H, dd, *J* = 1.5 Hz, *J* = 8.1 Hz, H-6′′), 8.18 (2H, d, *J* = 8.2 Hz, H-2′ and H-6′), 8.71 (1H, s, NH); ^13^C NMR (75 MHz, DMSO-*d*_6_): *δ* 21.0 (CH_3_), 125.7 (C-6′′), 126.9 (C-4′′), 127.2 (C-2′′), 127.8 (C-5′′, C-3′ and C-5′), 128.8 (C-2′ and C-6′), 129.3 (C-3′′), 133.8 (C-1′), 135.7 (C-1′′), 141.2 (C-4′), 165.1 (C-4), 167.2 (C-6), 170.2 (C-2). Anal. calcd for C_16_H_14_ClN_5_: C, 61.64; H, 4.53; N, 22.46. Found: C, 61.53; H, 4.35; N, 22.28.

#### 4.2.7. *N* ^2^-(4-Chlorophenyl)-6-(4-methylphenyl)-1,3,5-triazine-2,4-diamine (7)

Yield 26%. Mp 202–204 °C (MeCN). ^1^H NMR (300 MHz, DMSO-*d*_6_): *δ* 2.08 (3H, s, CH_3_), 7.14 (2H, brs, NH_2_), 7.32 (2H, d, *J* = 8.6 Hz, H-3′ and H-5′), 7.35 (2H, d, *J* = 9.0 Hz, H-3′′ and H-5′′), 7.90 (2H, d, *J* = 8.9 Hz, H-2′′ and H-6′′), 8.23 (2H, d, *J* = 8.2 Hz, H-2′ and H-6′), 9.65 (1H, s, NH); ^13^C NMR (75 MHz, DMSO-*d*_6_): *δ* 21.0 (CH_3_), 121.2 (C-2′′ and C-6′′), 125.4 (C-1′′), 127.8 (C-3′ and C-5′), 128.2 (C-3′′ and C-5′′), 128.8 (C-2′ and C-6′), 133.9 (C-1′), 139.0 (C-4′′), 141.3 (C-4′), 164.4 (C-4), 167.0 (C-6), 170.2 (C-2). Anal. calcd for C_16_H_14_ClN_5_: C, 61.64; H, 4.53; N, 22.46. Found: C, 61.49; H, 4.47; N, 22.32.

#### 4.2.8. 6-(4-Methoxyphenyl)-*N*^2^-phenyl-1,3,5-triazine-2,4-diamine (8)

Yield 50%. Mp 190–192 °C (EtOH). ^1^H NMR (300 MHz, DMSO-*d*_6_): *δ* 3.84 (3H, OCH_3_), 7.00 (1H, t, *J* = 7.4 Hz, H-4′′), 7.04 (2H, brs, NH_2_), 7.07 (2H, d, *J* = 9.0 Hz, H-3′ and H-5′), 7.31 (2H, dd, *J* = 7.5 Hz, *J* = 8.4 Hz, H-3′′ and H-5′′), 7.86 (2H, dd, *J* = 1.1 Hz, *J* = 8.6 Hz H-2′′ and H-6′′), 8.31 (2H, d, *J* = 9.0 Hz, H-2′ and H-6′), 9.46 (1H, s, NH); ^13^C NMR (75 MHz, DMSO-*d*_6_): *δ* 55.2 (OCH_3_), 113.6 (C-3′ and C-5′), 119.8 (C-2′′ and C-6′′), 121.8 (C-1′′), 128.3 (C-3′′ and C-5′′), 129.0 (C-1′), 129.5 (C-2′ and C-6′), 140.0 (C-4′′), 161.9 (C-4′), 164.5 (C-4), 167.0 (C-6), 169.8 (C-2). Anal. calcd for C_16_H_15_N_5_O: C, 65.52; H, 5.15; N, 23.88. Found: C, 65.52; H, 5.15; N, 23.88.

#### 4.2.9. 6-(4-Methoxyphenyl)-*N*^2^-(4-methylphenyl)-1,3,5-triazine-2,4-diamine (9)

Yield 41%. Mp 186–188 °C (MeCN). ^1^H NMR (300 MHz, DMSO-*d*_6_): *δ* 2.27 (3H, s, CH_3_), 3.84 (3H, s, OCH_3_), 6.99 (2H, brs, NH_2_), 7.06 (2H, d, *J* = 9.0 Hz, H-3′ and H-5′), 7.11 (2H, d, *J* = 8.3 Hz, H-3′′ and H-5′′), 7.71 (2H, d, *J* = 8.4 Hz, H-2′′ and H-6′′), 8.29 (2H, d, *J* = 9.0 Hz, H-2′ and H-6′), 9.35 (1H, s, NH); ^13^C NMR (75 MHz, DMSO-*d*_6_): *δ* 20.3 (CH_3_), 55.2 (OCH_3_), 113.5 (C-3′ and C-5′), 120.0 (C-2′′ and C-6′′), 128.7 (C-3′′ and C-5′′), 129.1 (C-1′′), 129.5 (C-2′ and C-6′), 130.7 (C-1′), 137.4 (C-4′′), 161.9 (C-4′), 164.4 (C-4), 167.0 (C-6), 169.7 (C-2). Anal. calcd for C_17_H_17_N_5_O: C, 66.43; H, 5.58; N, 22.79. Found: C, 66.32; H, 5.44; N, 22.68.

#### 4.2.10. *N* ^2^-Phenyl-6-(3,4,5-trimethoxyphenyl)-1,3,5-triazine-2,4-diamine (10)

Yield 48%. Mp 111–113 °C (EtOH). ^1^H NMR (300 MHz, DMSO-*d*_6_): *δ* 3.75 (3H, s, *p*-OCH_3_), 3.87 (6H, s, *m*-(OCH_3_)_2_), 6.99 (1H, t, *J* = 7.3 Hz, H-4′′), 7.11 (2H, brs, NH_2_), 7.30 (2H, dd, *J* = 7.7 Hz, *J* = 8.1 Hz, H-3′′ and H-5′′), 7.68 (2H, s, H-2′ and H-6′), 7.85 (2H, dd, *J* = 0.9 Hz, *J* = 8.5 Hz, H-2′′ and H-6′′), 9.51 (1H, s, NH); ^13^C NMR (75 MHz, DMSO-*d*_6_): *δ* 55.8 (*m*-(OCH_3_)_2_), 60.0 (*p*-OCH_3_), 105.0 (C-2′′and C-6′′), 119.9 (C-2′ and C-6′), 121.9 (C-1′), 128.3 (C-3′ and C-5′), 132.0 (C-1′′), 139.9 (C-4′), 140.3 (C-4′′), 152.6 (C-3′′ and C-5′′), 164.4 (C-4), 167.1 (C-6), 169.6 (C-2). Anal. calcd for C_18_H_19_N_5_O_3_: C, 61.18; H, 5.42; N, 19.82. Found: C, 60.96; H, 5.34; N, 19.67.

#### 4.2.11. *N* ^2^-(2-Fluorophenyl)-6-(3,4,5-trimethoxyphenyl)-1,3,5-triazine-2,4-diamine (11)

Yield 50%. Mp 177–179 °C (EtOH). ^1^H NMR (300 MHz, DMSO-*d*_6_): *δ* 3.74 (3H, *p*-OCH_3_), 3.84 (6H, *m*-(OCH_3_)_2_), 7.07 (2H, brs, NH_2_), 7.16–7.29 (3H, m, H-3′′, H-4′′ and H-5′′), 7.64 (2H, s, H-2′ and H-6′), 7.78–7.84 (1H, m, H-6′′), 9.03 (1H, s, NH); ^13^C NMR (75 MHz, DMSO-*d*_6_): *δ* 55.7 (*m*-(OCH_3_)_2_), 60.0 (*p*-OCH_3_), 105.0 (C-2′ and C-6′), 115.4 (d, ^2^*J*_CF_ = 19.4 Hz, C-3′′), 123.9 (d, ^3^*J*_CF_ = 3.4 Hz, C-4′′), 125.3 (d, ^3^*J*_CF_ = 8.4 Hz, C-6′′), 126.5 (d, ^4^*J*_CF_ = 1.5 Hz, C-5′′), 126.7 (d, ^2^*J*_CF_ = 10.8 Hz, C-1′′), 131.9 (C-1′), 140.3 (C-4′), 152.5 (C-3′ and C-5′), 155.3 (d, ^1^*J*_CF_ = 245.9 Hz, C-2′′), 165.0 (C-4), 167.3 (C-6), 169.6 (C-2). Anal. calcd for C_18_H_19_FN_5_O_3_: C, 58.22; H, 4.89; N, 18.86. Found: C, 58.05; H, 4.79; N, 18.68.

#### 4.2.12. *N* ^2^-(2-Chlorophenyl)-6-(3,4,5-trimethoxyphenyl)-1,3,5-triazine-2,4-diamine (12)

Yield 43%. Mp 176–178 °C (EtOH). ^1^H NMR (300 MHz, DMSO-*d*_6_): *δ* 3.75 (3H, *p*-OCH_3_), 3.84 (6H, *m*-(OCH_3_)_2_), 7.11 (2H, brs, NH_2_), 7.19 (1H, ddd, *J* = 1.6 Hz, *J* = 7.6 Hz, *J* = 7.8 Hz, H-4′′), 7.36 (1H, ddd, *J* = 1.3 Hz, *J* = 7.6 Hz, *J* = 7.8 Hz, H-5′′), 7.51 (1H, dd, *J* = 1.4 Hz, *J* = 8.0 Hz, H-3′′), 7.64 (2H, s, H-2′ and H-6′), 7.87 (1H, dd, *J* = 1.5 Hz, *J* = 8.1 Hz, H-6′′), 8.80 (1H, s, NH); ^13^C NMR (75 MHz, DMSO-*d*_6_): *δ* 55.7 (*m*-(OCH_3_)_2_), 60.0 (*p*-OCH_3_), 105.0 (C-2′ and C-6′), 125.8 (C-6′′), 127.1 (C-2′′ and C-4′′), 127.9 (C-5′′), 129.3 (C-3′′), 131.8 (C-1′), 135.8 (C-1′′), 140.4 (C-4′), 152.6 (C-3′ and C-5′), 165.0 (C-4), 167.3 (C-6), 169.7 (C-2). Anal. calcd for C_18_H_19_ClN_5_O_3_: C, 55.75; H, 4.68; N, 18.06. Found: C, 55.49; H, 4.57; N, 17.90.

#### 4.2.13. *N* ^2^-(2-Methoxyphenyl)-6-(3,4,5-trimethoxyphenyl)-1,3,5-triazine-2,4-diamine (13)

Yield 49%. Mp 173–175 °C (EtOH). ^1^H NMR (300 MHz, DMSO-*d*_6_): *δ* 3.75 (3H, *p*-OCH_3_), 3.86 (9H, *m*-(OCH_3_)_2_ and *o*-OCH_3_), 6.96 (1H, ddd, *J* = 4.2 Hz, *J* = 4.2 Hz, *J* = 8.4 Hz, H-4′′), 7.05–7.07 (2H, m, H-3′′ and H-5′′), 7.15 (2H, brs, NH_2_), 7.67 (2H, s, H-2′ and H-6′), 8.02 (1H, s, NH), 8.24 (1H, d, *J* = 7.5 Hz, H-6′′); ^13^C NMR (75 MHz, DMSO-*d*_6_): *δ* 55.7 (*o*-OCH_3_), 55.7 (*m*-(OCH_3_)_2_), 60.0 (*p*-OCH_3_), 105.0 (C-2′ and C-6′), 110.9 (C-3′′), 120.1 (C-5′′), 122.0 (C-6′′), 123.4 (C-4′′), 127.8 (C-1′′), 131.8 (C-1′), 140.4 (C-4′), 149.6 (C-2′′), 152.6 (C-3′ and C-5′), 164.5 (C-4), 167.2 (C-6), 169.6 (C-2). Anal. calcd for C_19_H_21_N_5_O_4_: C, 59.52; H, 5.52; N, 18.27. Found: C, 59.42; H, 5.39; N, 18.05.

#### 4.2.14. *N* ^2^-(3-Chlorophenyl)-6-(3,4,5-trimethoxyphenyl)-1,3,5-triazine-2,4-diamine (14)

Yield 52%. Mp 193–195 °C (EtOH). ^1^H NMR (300 MHz, DMSO-*d*_6_): *δ* 3.76 (3H, *p*-OCH_3_), 3.89 (6H, *m*-(OCH_3_)_2_), 7.03 (1H, ddd, *J* = 0.8 Hz, *J* = 2.0 Hz *J* = 8.0 Hz, H-4′′), 7.24 (2H, br s, NH_2_), 7.32 (1H, dd, *J* = 8.1 Hz, *J* = 8.1 Hz, H-5′′), 7.67 (3H, m, H-6′′, H-2′ and H-6′), 8.20 (1H, s, H-2′′), 9.74 (1H, s, NH); ^13^C NMR (75 MHz, DMSO-*d*_6_): *δ* 55.8 (*m*-(OCH_3_)_2_), 60.1 (*p*-OCH_3_), 105.0 (C-2′ and C-6′), 118.1 (C-6′′), 119.1 (C-2′′), 121.4 (C-1′′), 129.9 (C-5′′), 131.8 (C-1′), 132.8 (C-3′′), 140.5 (C-4′), 141.6 (C-4′′), 152.6 (C-3′ and C-5′), 164.4 (C-4), 167.0 (C-6), 169.8 (C-2). Anal. calcd for C_18_H_19_ClN_5_O_3_: C, 55.75; H, 4.68; N, 18.06. Found: C, 55.63; H, 4.54; N, 17.86.

#### 4.2.15. *N* ^2^-(3-Methylphenyl)-6-(3,4,5-trimethoxyphenyl)-1,3,5-triazine-2,4-diamine (15)

Yield 55%. Mp 192–194 °C (EtOH). ^1^H NMR (300 MHz, DMSO-*d*_6_): *δ* 2.31 (3H, s, CH_3_), 3.76 (3H, *p*-OCH_3_), 3.88 (6H, *m*-(OCH_3_)_2_), 6.82 (1H, d, *J* = 7.4 Hz, H-4′′), 7.11 (2H, br s, NH_2_), 7.18 (1H, dd, *J* = 7.8 Hz, *J* = 7.8 Hz, H-5′′), 7.63 (1H, d, *J* = 8.4 Hz, H-6′′), 7.71 (2H, s, H-2′ and H-6′), 7.75 (1H, s, H-2′′), 9.44 (1H, s, NH); ^13^C NMR (75 MHz, DMSO-*d*_6_): *δ* 21.3 (CH_3_), 55.8 (*m*-(OCH_3_)_2_), 60.1 (*p*-OCH_3_), 105.0 (C-2′ and C-6′), 117.2 (C-6′′), 120.5 (C-2′′), 122.7 (C-1′′), 128.1 (C-5′′), 132.1 (C-1′), 137.4 (C-4′′), 139.9 (C-3′′), 140.4 (C-4′), 152.6 (C-3′ and C-5′), 164.5 (C-4), 167.1 (C-6), 169.6 (C-2). Anal. calcd for C_19_H_21_N_5_O_3_: C, 62.11; H, 5.76; N, 19.06. Found: C, 61.93; H, 5.64; N, 18.88.

#### 4.2.16. *N* ^2^-(4-Chlorophenyl)-6-(3,4,5-trimethoxyphenyl)-1,3,5-triazine-2,4-diamine (16)

Yield 53%. Mp 209–211 °C (EtOH). ^1^H NMR (300 MHz, DMSO-*d*_6_): *δ* 3.75 (3H, s, *p*-OCH_3_), 3.87 (6H, s, *m*-(OCH_3_)_2_), 7.19 (2H, brs, NH_2_), 7.35 (2H, d, *J* = 8.9 Hz, H-3′′ and H-5′′), 7.68 (2H, s, H-2′ and H-6′), 7.90 (2H, d, *J* = 8.9 Hz, H-2′′ and H-6′′), 9.67 (1H, s, NH); ^13^C NMR (75 MHz, DMSO-*d*_6_): *δ* 55.8 (*m*-(OCH_3_)_2_), 60.0 (*p*-OCH_3_), 105.0 (C-2′ and C-6′), 121.2 (C-2′′ and C-6′′), 125.5 (C-1′′), 128.1 (C-3′′ and C-5′′), 131.9 (C-1′), 139.0 (C-4′′), 140.4 (C-4′), 152.6 (C-3′ and C-5′), 164.3 (C-4), 167.0 (C-6), 169.7 (C-2). Anal. calcd for C_18_H_19_ClN_5_O_3_: C, 55.75; H, 4.68; N, 18.06. Found: C, 55.62; H, 4.55; N, 17.94.

#### 4.2.17. *N* ^2^-(4-Bromophenyl)-6-(3,4,5-trimethoxyphenyl)-1,3,5-triazine-2,4-diamine (17)

Yield 61%. Mp 217–219 °C (EtOH). ^1^H NMR (300 MHz, DMSO-*d*_6_): *δ* 3.76 (3H, s, *p*-OCH_3_), 3.87 (6H, s, *m*-(OCH_3_)_2_), 7.19 (2H, brs, NH_2_), 7.47 (2H, d, *J* = 8.9 Hz, H-3′′ and H-5′′), 7.68 (2H, s, H-2′ and H-6′), 7.86 (2H, d, *J* = 8.9 Hz, H-2′′ and H-6′′), 9.68 (1H, s, NH); ^13^C NMR (75 MHz, DMSO-*d*_6_): *δ* 55.8 (*m*-(OCH_3_)_2_), 60.0 (*p*-OCH_3_), 105.0 (C-2′ and C-6′), 113.4 (C-1′′), 121.7 (C-2′′ and C-6′′), 131.0 (C-3′′ and C-5′′), 131.9 (C-1′), 139.4 (C-4′′), 140.4 (C-4′), 152.6 (C-3′ and C-5′), 164.3 (C-4), 167.0 (C-6), 169.7 (C-2). Anal. calcd for C_18_H_18_BrN_5_O_3_: C, 50.01; H, 4.20; N, 16.20. Found: C, 49.85; H, 4.09; N, 16.03.

#### 4.2.18. *N* ^2^-(4-Methylphenyl)-6-(3,4,5-trimethoxyphenyl)-1,3,5-triazine-2,4-diamine (18)

Yield 55%. Mp 208–210 °C (EtOH). ^1^H NMR (300 MHz, DMSO-*d*_6_): *δ* 2.26 (3H, s, CH_3_), 3.75 (3H, s, *p*-OCH_3_), 3.86 (6H, s, *m*-(OCH_3_)_2_), 7.07 (2H, brs, NH_2_), 7.10 (2H, d, *J* = 8.3 Hz, H-3′′ and H-5′′), 7.68 (2H, s, H-2′ and H-6′), 7.72 (2H, d, *J* = 8.3 Hz, H-2′′ and H-6′′), 9.41 (1H, s, NH); ^13^C NMR (75 MHz, DMSO-*d*_6_): *δ* 20.3 (CH_3_), 55.7 (*m*-(OCH_3_)_2_), 60.0 (*p*-OCH_3_), 105.0 (C-2′ and C-6′), 120.0 (C-2′′and C-6′′), 128.7 (C-3′′ and C-5′′), 130.8 (C-1′′), 132.1 (C-1′), 137.3 (C-4′′), 140.3 (C-4′), 152.6 (C-3′ and C-5′), 164.3 (C-4), 167.0 (C-6), 169.5 (C-2). Anal. calcd for C_19_H_21_N_5_O_3_: C, 62.11; H, 5.76; N, 19.06. Found: C, 61.97; H, 5.65; N, 18.91.

#### 4.2.19. *N* ^2^-(4-Methoxyphenyl)-6-(3,4,5-trimethoxyphenyl)-1,3,5-triazine-2,4-diamine (19)

Yield 52%. Mp 195–197 °C (EtOH). ^1^H NMR (300 MHz, DMSO-*d*_6_): *δ* 3.74 (3H, s, *p*-OCH_3_), 3.75 (3H, s, *p*-OCH_3_), 3.86 (6H, s, *m*-(OCH_3_)_2_), 6.89 (2H, d, *J* = 9.0 Hz, H-3′′ and H-5′′), 7.03 (2H, brs, NH_2_), 7.68 (2H, s, H-2′ and H-6′), 7.71 (2H, d, *J* = 9.1 Hz, H-2′′ and H-6′′), 9.34 (1H, s, NH); ^13^C NMR (75 MHz, DMSO-*d*_6_): *δ* 55.1 (*p*-OCH_3_), 55.7 (*m*-(OCH_3_)_2_), 60.0 (*p*-OCH_3_), 104.9 (C-2′ and C-6′), 113.5 (C-3′′ and C-5′′), 121.7 (C-2′′ and C-6′′), 132.1 (C-1′), 132.9 (C-1′′), 140.2 (C-4′), 152.5 (C-3′ and C-5′), 154.6 (C-4′′), 164.3 (C-4), 167.1 (C-6), 169.4 (C-2). Anal. calcd for C_19_H_21_N_5_O_4_: C, 59.52; H, 5.52; N, 18.27. Found: C, 59.36; H, 5.44; N, 18.12.

#### 4.2.20. *N* ^2^-(4-(Trifluoromethoxy)phenyl)-6-(3,4,5-trimethoxyphenyl)-1,3,5-triazine-2,4-diamine (20)

Yield 56%. Mp 179–181 °C (EtOH). ^1^H NMR (300 MHz, DMSO-*d*_6_): *δ* 3.75 (3H, s, *p*-OCH_3_), 3.87 (6H, s, *m*-(OCH_3_)_2_), 7.20 (2H, brs, NH_2_), 7.30 (2H, d, *J* = 8.8 Hz, H-3′′ and H-5′′), 7.67 (2H, s, H-2′ and H-6′), 7.95 (2H, d, *J* = 9.1 Hz, H-2′′ and H-6′′), 9.72 (1H, s, NH); ^13^C NMR (75 MHz, DMSO-*d*_6_): *δ* 55.8 (*m*-(OCH_3_)_2_), 60.1 (*p*-OCH_3_), 105.0 (C-2′ and C-6′), 120.2 (q, ^1^*J*_CF_ = 255.1 Hz, OCF_3_), 121.1 (C-3′′ and C-5′′), 121.2 (C-2′′ and C-6′′), 131.9 (C-1′), 139.2 (C-1′′), 140.4 (C-4′), 142.7 (q, ^3^*J*_CF_ = 1.7 Hz, C-4′′), 152.6 (C-3′ and C-5′), 164.4 (C-4), 167.1 (C-6), 169.7 (C-2). Anal. calcd for C_19_H_18_F_3_N_5_O_4_: C, 52.18; H, 4.15; N, 16.01. Found: C, 52.07; H, 4.08; N, 15.96.

#### 4.2.21. *N* ^2^-(4-Isopropylphenyl)-6-(3,4,5-trimethoxyphenyl)-1,3,5-triazine-2,4-diamine (21)

Yield 49%. Mp 190–192 °C (EtOH). ^1^H NMR (300 MHz, DMSO-*d*_6_): *δ* 1.20 (6H, d, *J* = 6.9 Hz, CH(CH̲_3_)_2_), 2.85 (1H, sept, *J* = 6.9 Hz, CH̲(CH_3_)_2_), 3.75 (3H, s, *p*-OCH_3_), 3.87 (6H, s, *m*-(OCH_3_)_2_), 7.06 (2H, brs, NH_2_), 7.16 (2H, d, *J* = 8.6 Hz, H-3′′ and H-5′′), 7.68 (2H, s, H-2′ and H-6′), 7.72 (2H, d, *J* = 8.6 Hz, H-2′′ and H-6′′), 9.41 (1H, s, NH); ^13^C NMR (75 MHz, DMSO-*d*_6_): *δ* 23.9 (CH(C̲H_3_)_2_), 32.7 (C̲H(CH_3_)_2_), 55.7 (*m*-(OCH_3_)_2_), 60.0 (*p*-OCH_3_), 105.0 (C-2′ and C-6′), 120.3 (C-2′′ and C-6′′), 126.0 (C-3′′ and C-5′′), 132.1 (C-1′), 137.5 (C-1′′), 140.3 (C-4′), 142.1 (C-4′′), 152.6 (C-3′ and C-5′), 164.4 (C-4), 167.1 (C-6), 169.5 (C-2). Anal. calcd for C_21_H_25_N_5_O_3_: C, 63.78; H, 6.37; N, 17.71. Found: C, 63.65; H, 6.22; N, 17.54.

### 4.3. X-ray structure determination of 20

Intensity data for a colourless crystal of 20 (0.05 × 0.09 × 0.13 mm) were measured at 100 K on an XtaLAB Synergy Dual Atlas diffractometer equipped with a CCD area detector and graphite-monochromated Cu Kα radiation (*λ* = 1.54184 Å) so that *θ*_max_ = 67.1°. Data reduction and empirical absorption corrections, based on a multi-scan technique, were applied.^23^ The structure was solved by direct methods^24^ and refined on *F*^2^ with anisotropic displacement parameters and C-bound H atoms in the riding model approximation.^25^ The nitrogen-bound H atoms were refined with a distance restraint N–H = 0.88 ± 0.01 Å. A weighting scheme of the form *w* = 1/[*σ*^2^(*F*_o_^2^) + (0.104*P*)^2^ + 1.179*P*] where *P* = (*F*_o_^2^ + 2*F*_c_^2^)/3 was introduced. The final refinement on 292 parameters yielded *R* = 0.059 (3044 data with *I* ≥ 2*σ*(*I*)) and *wR*_2_ = 0.176 (all 3357 data). The maximum and minimum residual electron density peaks of 1.73 and 0.62 eÅ^−3^, respectively, were located 1.85 and 0.69 Å from the C19 and F1 atoms, respectively, that is, in chemically non-sensible positions. The molecular structure diagram was generated at the 70% probability level by ORTEP for Windows,^26^ and the packing diagrams were generated with DIAMOND.^27^ Additional analysis was conducted with PLATON.^28^

Crystal data for C_19_H_18_F_3_N_5_O_4_: *M* = 437.38, triclinic, *P*1̄, *a* = 7.2212(2), *b* = 10.9597(3), *c* = 13.4202(3) Å, *α* = 104.412(2)°, *β* = 99.253(2)°, *γ* = 108.517(2)°, *V* = 941.57(4) Å^3^, *Z* = 2, *D*_x_ = 1.543 g cm^−3^, *F*(000) = 452 and *μ* = 1.125 mm^−1^.

### 4.4. *In vitro* cytotoxicity assay

The synthesized compounds were tested against three breast tumor cell lines (MDA-MB231, SKBR-3 and MCF-7) and epithelial breast cell line (MCF-10A) by the MTT colorimetric assay.^29,30^ All cells were obtained from the American Type Culture Collection. The cancerous cell lines were grown in Dulbecco's modified eagle medium (DMEM) supplemented with 10% fetal bovine serum (FBS) and 1% pen-strep antibiotic. The MCF-10A human epithelial breast cell line was grown in the complete mammary epithelial growth medium containing horse serum 5%, epithelial growth factor 20 ng mL^−1^, hydrocortisone 0.5 mg mL^−1^, cholera toxin 100 ng mL^−1^, insulin 10 μg mL^−1^, and pen-step antibiotic.^31^ For the cytotoxic assay, 20 to 75 × 10^3^ cells per mL (based on the doubling time for each cell line) were seeded in 96-well plates and the plates were incubated overnight in a humidified air atmosphere at 37 °C in 5% CO_2_ incubator.

The cells were then treated with compounds at different concentrations. After 72 h of incubation, the MTT (0.5 mg mL^−1^) was added to wells, followed by 4 h of incubation. The culture medium was then removed and DMSO (100 μL per well) was added and the absorbance values were measured at 570 nm using the multi-well Tecan NanoQuant, Infinite m200 Pro plate reader. Growth inhibitory values (GI_50_) were calculated using GraphPad Prism 7 (GraphPad Software, San Diego, USA) by nonlinear regression analysis. Three independent experiments were carried out and the data was expressed in mean ± standard deviation (SD). The concentration–response curves used for the GI_50_ calculation are available in ESI.†

### 4.5. Time-dependent cytotoxicity

MDA-MB231 breast cancer cells were grown in DMEM supplemented with 10% FBS and 1% pen-strep antibiotic. The cells were seeded in 96-well plates (20 × 10^3^ cells per mL) and the plates were incubated overnight in a humidified air atmosphere at 37 °C in 5% CO_2_ incubator. The cells were then treated with compounds 16 or 18 in concentrations ranging from 0.2 nM to 125 nM for 12, 24, 48, and 72 h. After specified time for each experiment, MTT (0.5 mg mL^−1^) was added to wells, followed by 4 h of incubation. The culture medium was then removed and DMSO (100 μL per well) was added and the absorbance values were measured at 570 nm using the multi-well Tecan NanoQuant, Infinite m200 Pro plate reader. GI_50_ values were calculated using GraphPad Prism 7 (GraphPad Software, San Diego, USA) by nonlinear regression analysis. All the time-dependent experiments at different times were carried out using the same passage of MDA-MB231 cells. Three independent experiments were carried out and the data were expressed in mean ± standard deviation (SD).

### 4.6. Acridine orange/propidium iodide staining

Fluorescence microscopy was used to visualize the apoptosis in cancer cells with AO/PI staining.^20^ MDA-MB231 cells were grown in 6-well plate (5 × 10^5^ cells per well). The cells were treated with respective compounds at concentrations equal to estimated GI_20_ and GI_50_ values and incubated for 24 h. One of the six wells was treated with 1% DMSO and served as a negative control, another well treated with methotrexate served as a positive control. After 24 h of incubation, the wells were washed with phosphate buffered saline (PBS) three times and 100 μL of AO (100 μg mL^−1^ in PBS) and 25 μL of PI (100 μg mL^−1^ in PBS) in 1 mL of media were added to each well. The plate was then observed under a motorized inverted fluorescent microscope (Eclipse Ti2-E, Nikon). Three independent experiments at each concentration were carried out.

### 4.7. Live cell imaging

MDA-MB231 cells (1 × 10^6^ cells) were seeded in a Petri dish and incubated overnight in a humidified air atmosphere at 37 °C in 5% CO_2_ incubator. Then, the Petri dish was washed with PBS three times and 300 μL of AO (100 μg mL^−1^ in PBS) and 75 μL of PI (100 μg mL^−1^ in PBS) in 3 mL of media were added to the Petri dish. The cells were treated with 10 μM of compound 18 and the dish was then transferred to motorized inverted fluorescent microscope (Eclipse Ti2-E, Nikon). The pictures were taken every 10 minutes for 4 h and intercalated into video (see ESI†).

### 4.8. QSAR model testing

The previously reported^16^ 3D-QSAR model for 6,*N*^2^-1,3,5-triazine-2,4-diamines against MDA-MB231 breast carcinoma was applied. The structures of the proposed compounds were drawn using ChemDraw 15.0 and imported to Discovery Studio v18 (ref. 32) for the activity prediction. The structures were prepared for the 3D-QSAR modeling and aligned to minimum energy using the ‘align small molecules’ protocol, which is based on 50% steric and 50% electrostatic fields for alignment of molecules. The predicted pGI_50_ values were then obtained by the ‘calculate molecular properties’ protocol in Discover Studio using the previously prepared 3D-QSAR model.

### 4.9. ADME properties prediction

QikProp module of the Schrodinger^33^ was used to predict the absorption, distribution, metabolism and excretion (ADME) properties. The QikProp module predicts the descriptors which are pharmaceutically significant to identify the relevant properties of the organic molecule in relation to the 95% of the marketed drugs. The molecules were drawn and prepared (energy minimized and aligned) in Maestro program (v10.1) of Schrodinger software suit. QikProp (v4.3) was run with default options in normal processing mode.

## Conflicts of interest

There are no conflicts to declare.

## Supplementary Material

RA-010-D0RA04970K-s001

RA-010-D0RA04970K-s002

RA-010-D0RA04970K-s003
