# Supplementary material for: Design, synthesis, and biological evaluation of new 6,N2-diaryl-1,3,5-triazine-2,4-diamines as anticancer agents selectively targeting triple negative breast cancer cells
Source: RSC Adv. 2020 Jul 6;10(43):25517–28. doi: 10.1039/d0ra04970k (PMC9055250; doi:10.1039/d0ra04970k)
Supplement: RA-010-D0RA04970K-s002 [file RA-010-D0RA04970K-s002.pdf]

Electronic Supplementary Material (ESI) for RSC Advances.
This journal is © The Royal Society of Chemistry 2020

RSC Advances

Electronic supplementary information (ESI)

Design, synthesis, and biological evaluation of new 6,*N*[2]-diaryl-1,3,5-triazine-2,4-diamines as anticancer agents selectively targeting triple negative breast cancer cells

Ahmad Junaid,[a] Felicia Phei Lin Lim,[a] Edward R. T. Tiekink[b] and Anton V. Dolzhenko*[ac]

[a] School of Pharmacy, Monash University Malaysia, Jalan Lagoon Selatan, Bandar Sunway, Selangor Darul Ehsan 47500, Malaysia

[b] Research Centre for Crystalline Materials, School of Science and Technology, 5 Jalan Universiti, Sunway University, Bandar Sunway, Selangor Darul Ehsan 47500, Malaysia

[c] School of Pharmacy and Biomedical Sciences, Curtin Health Innovation Research Institute, Faculty of Health Sciences, Curtin University, GPO Box U1987 Perth, Western Australia 6845, Australia

Contents

Copies of ^{1}H and ^{13}C NMR spectra of 6,N^2,-diaryl-1,3,5-triazine-2,4-diamines **1-21** …………..S3

X-ray structure analysis of N^2-(4-(trifluoromethoxy)phenyl)-

6-(3,4,5-trimethoxyphenyl)-1,3,5-triazine-2,4-diamine (**20**) ……………………………..……..S25

Concentration-response curves for the compounds tested against

MDA-MB231 breast cancer cells ………………………………………………………….…S27

Concentration-response curves for the most active compounds tested against

SKBR-3 breast cancer cells ……………………………………………………………..S31

Concentration-response curves for the most active compounds tested against

MCF-7 breast cancer cells ………………………………………………………………S34

Time-dependent concentration-response curves for compounds **16** and **18** tested against

MDA-MB231 breast cancer cells ………………………………………………………….S35

Predicted ADME properties of compounds **1-21** ………………………………………….S36

Copies of ^{1}H and ^{13}C NMR spectra of 6,*N*^{2},-diaryl-1,3,5-triazine-2,4-diamines 1-21

6-(3-Fluorophenyl)-N^2-(2-methoxyphenyl)-1,3,5-triazine-2,4-diamine (1)

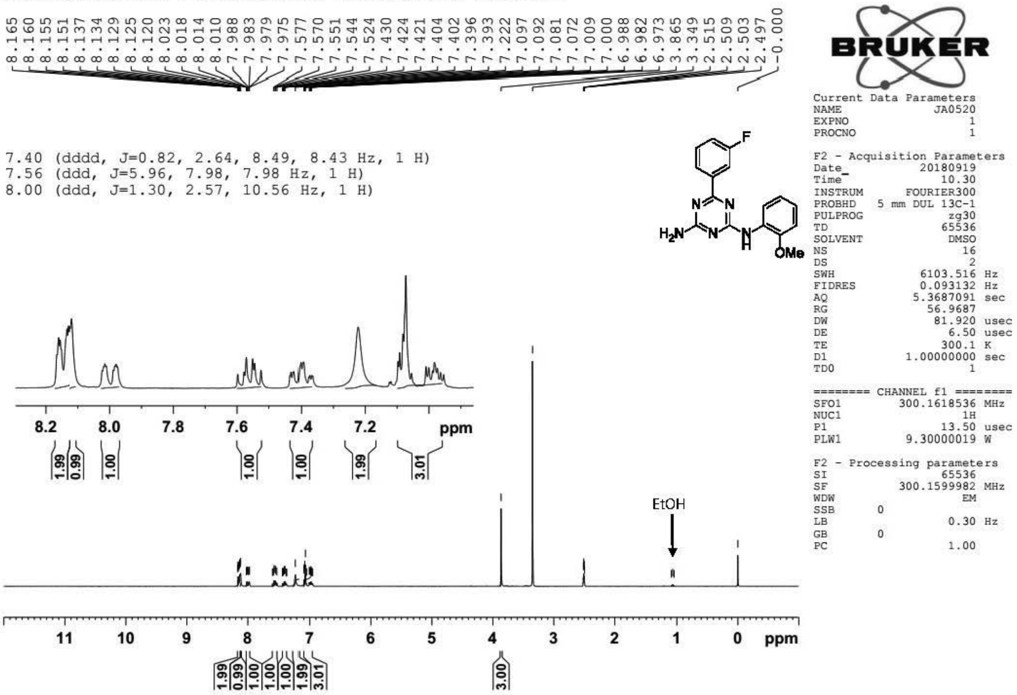

6-(3-Fluorophenyl)-N^2-(2-methoxyphenyl)-1,3,5-triazine-2,4-diamine (1)

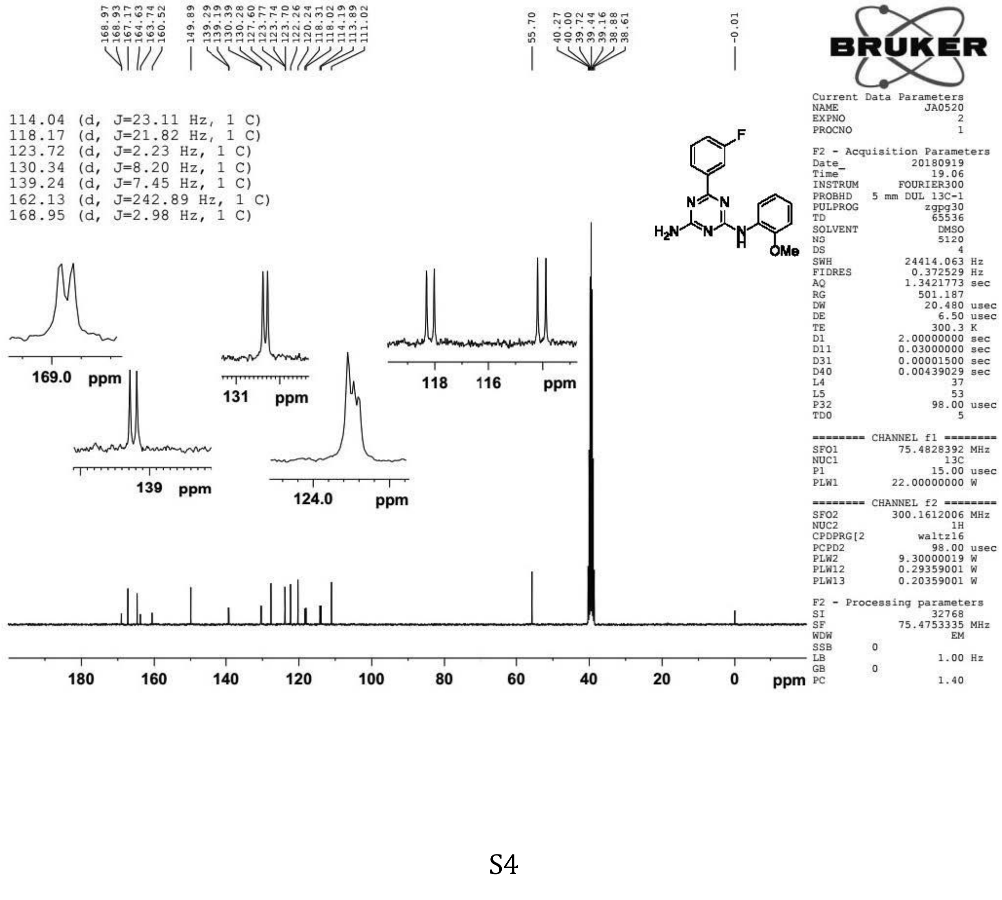

6-(4-Chlorophenyl)-N²-(p-tolyl)-1,3,5-triazine-2,4-diamine (2)

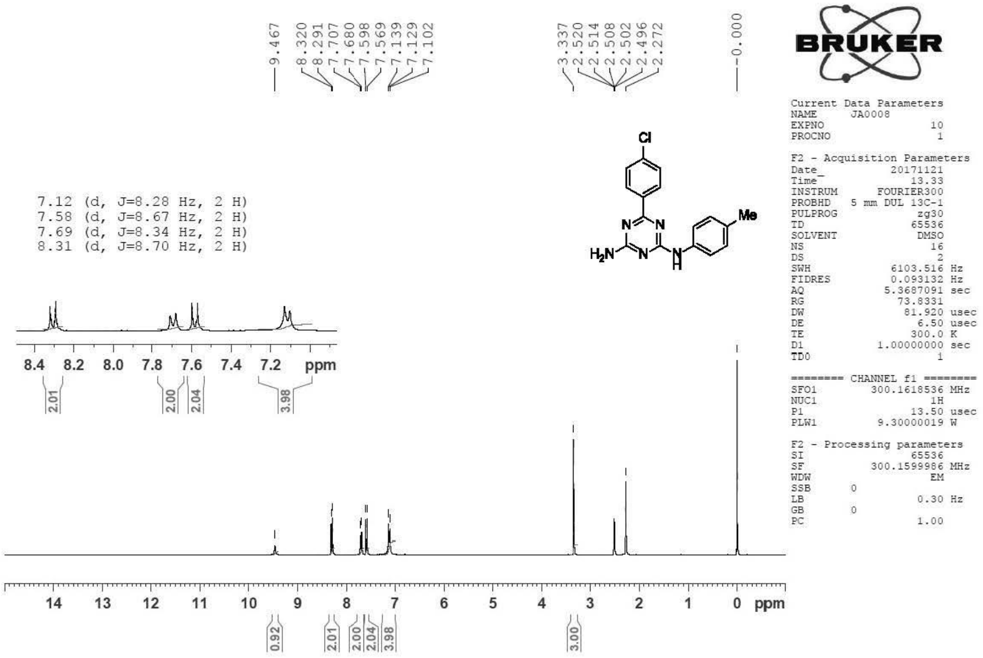

6-(4-Chlorophenyl)-N²-(p-tolyl)-1,3,5-triazine-2,4-diamine (2)

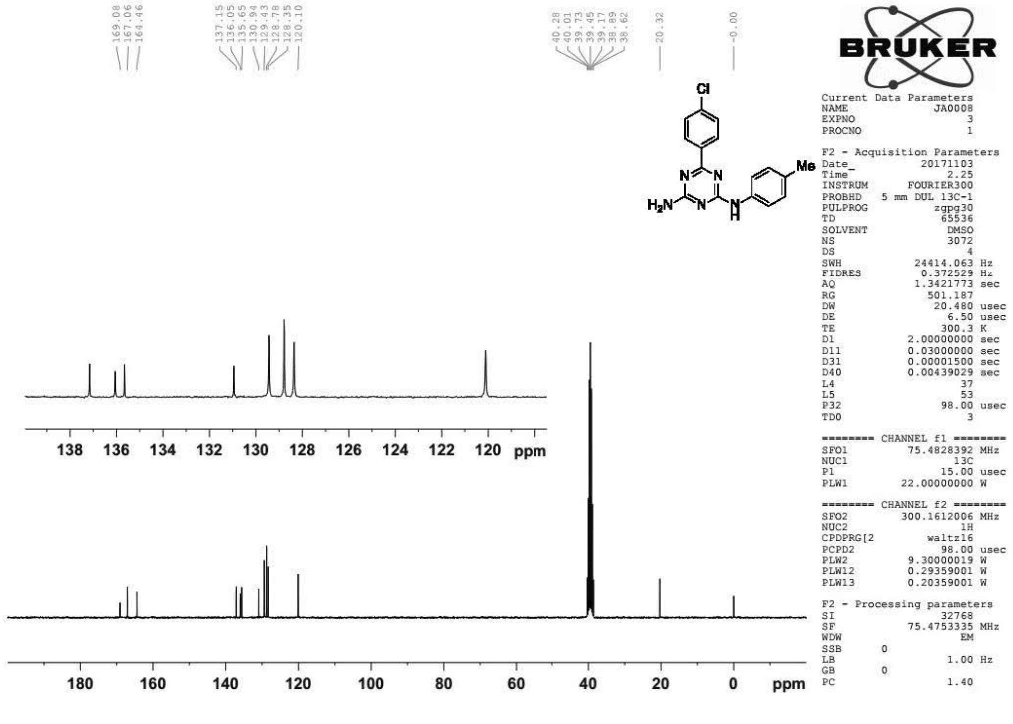

N[2]-(2-Chlorophenyl)-6-(4-(trifluoromethyl)phenyl)-1,3,5-triazine-2,4-diamine (3)*

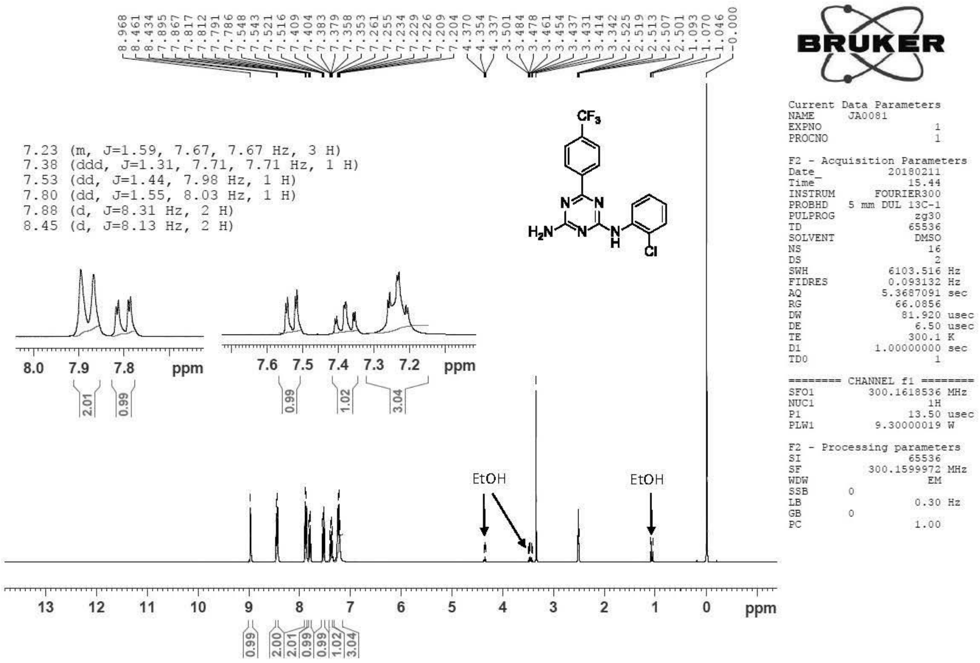

N[2]-(2-Chlorophenyl)-6-(4-(trifluoromethyl)phenyl)-1,3,5-triazine-2,4-diamine (3)*

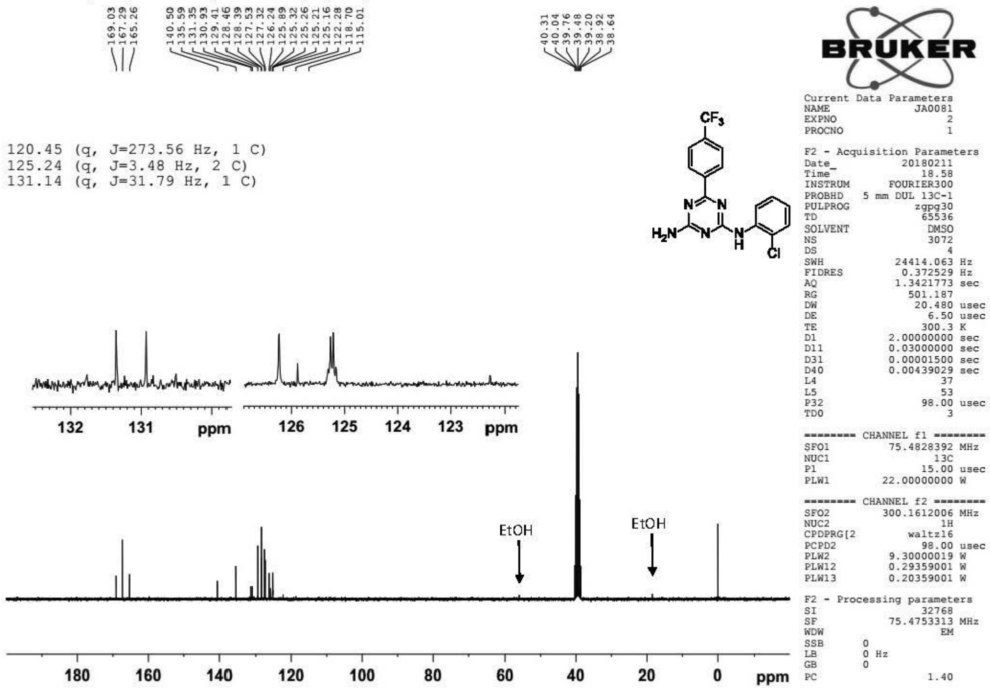

N[2]-(4-Chlorophenyl)-6-(4-(trifluoromethoxy)phenyl)-1,3,5-triazine-2,4-diamine (4)

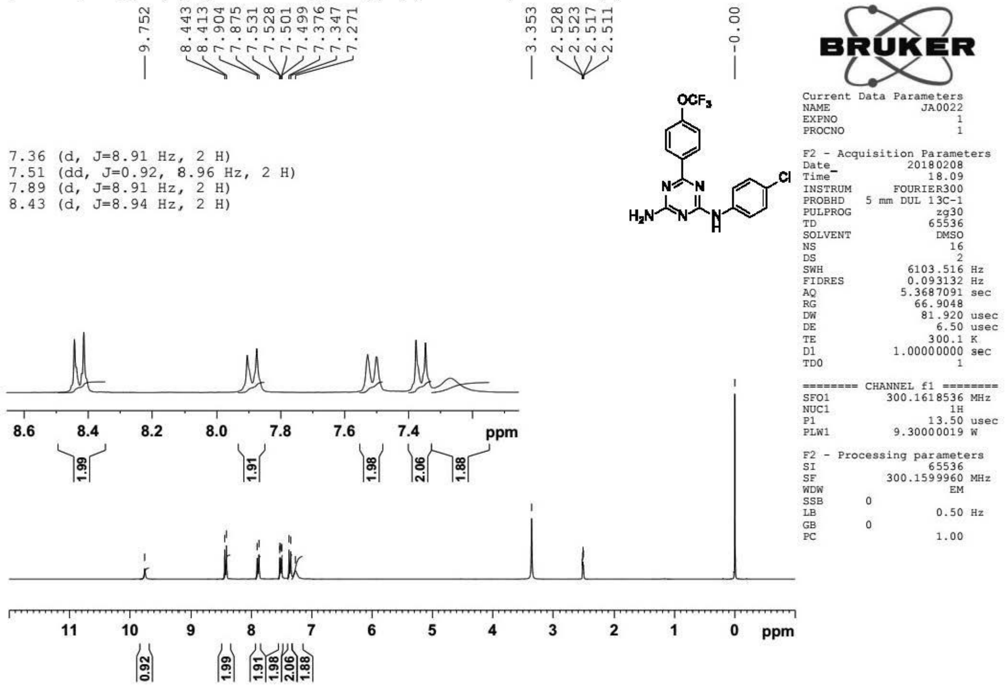

N[2]-(4-Chlorophenyl)-6-(4-(trifluoromethoxy)phenyl)-1,3,5-triazine-2,4-diamine (4)

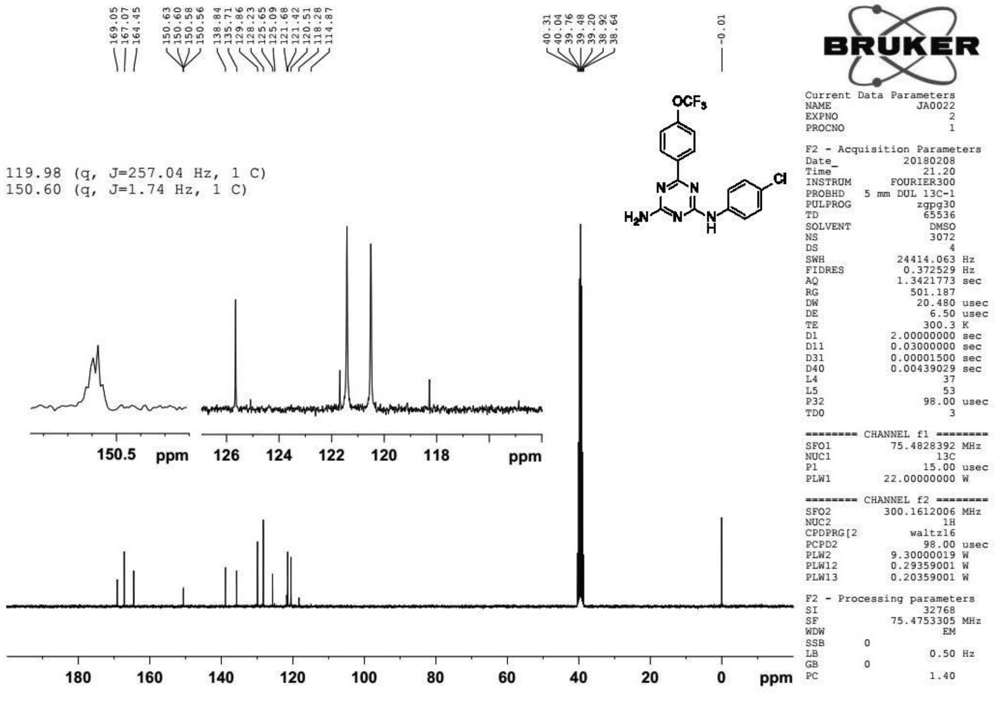

N[2]-(2-Chlorophenyl)-6-(4-(dimethylamino)phenyl)-1,3,5-triazine-2,4-diamine (5)

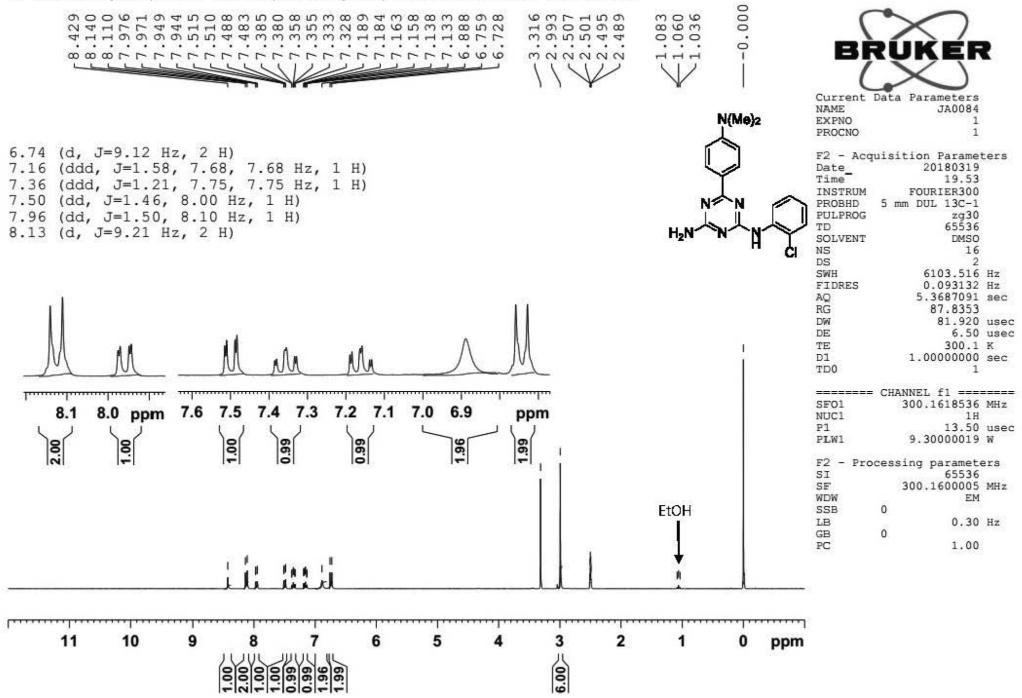

N[2]-(2-Chlorophenyl)-6-(4-(dimethylamino)phenyl)-1,3,5-triazine-2,4-diamine (5)

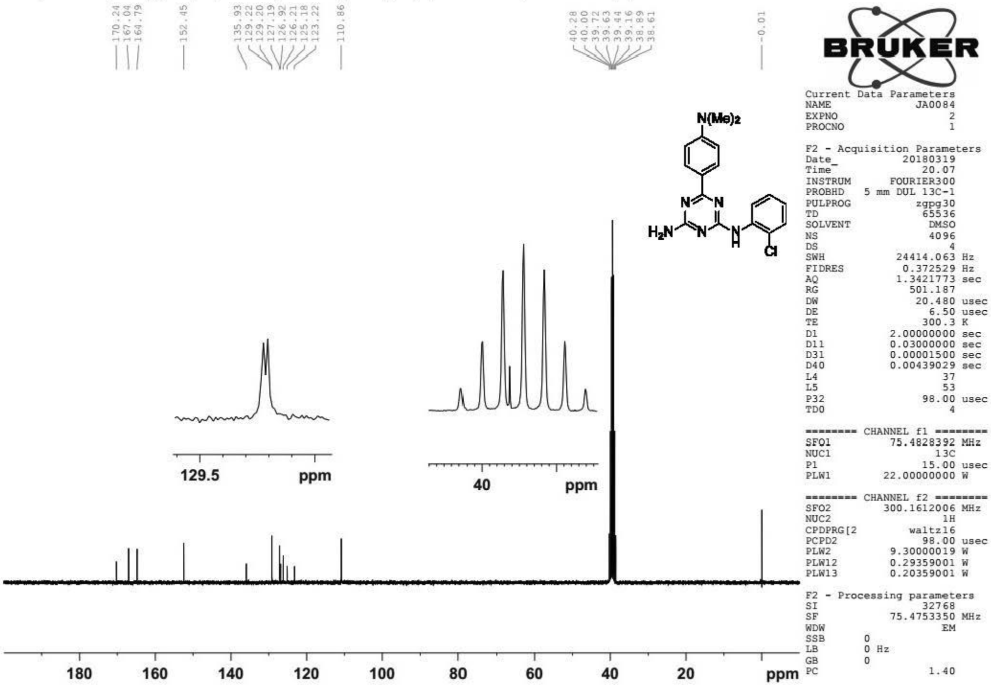

N[2]-(2-Chlorophenyl)-6-(p-tolyl)-1,3,5-triazine-2,4-diamine (6)

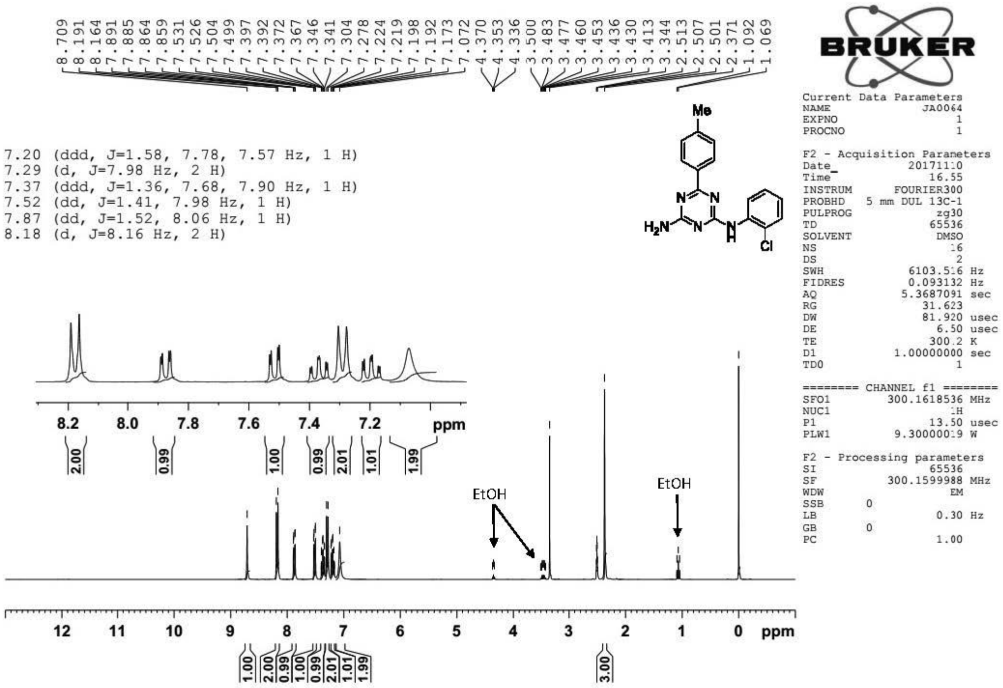

N[2]-(2-Chlorophenyl)-6-(p-tolyl)-1,3,5-triazine-2,4-diamine (6)

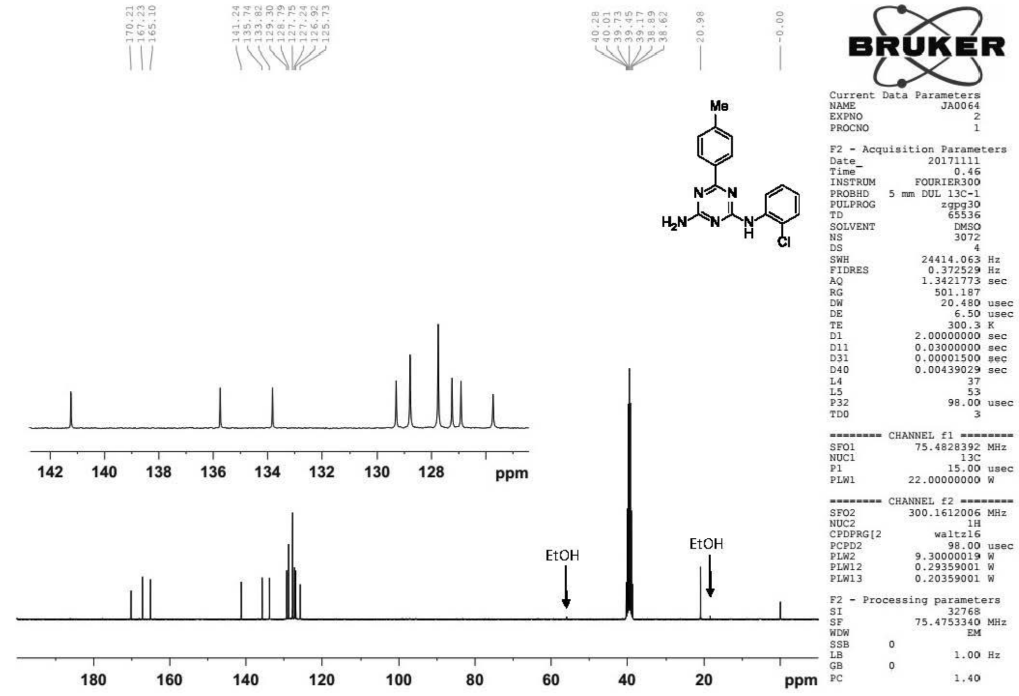

N[2]-(4-Chlorophenyl)-6-(*p*-tolyl)-1,3,5-triazine-2,4-diamine (7)

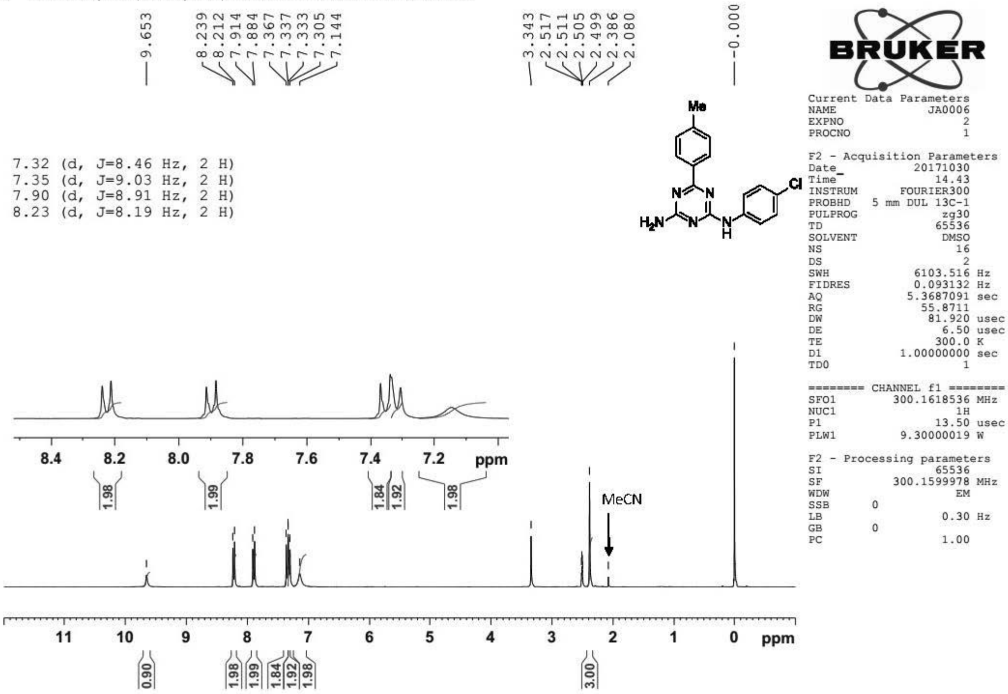

N[2]-(4-Chlorophenyl)-6-(*p*-tolyl)-1,3,5-triazine-2,4-diamine (7)

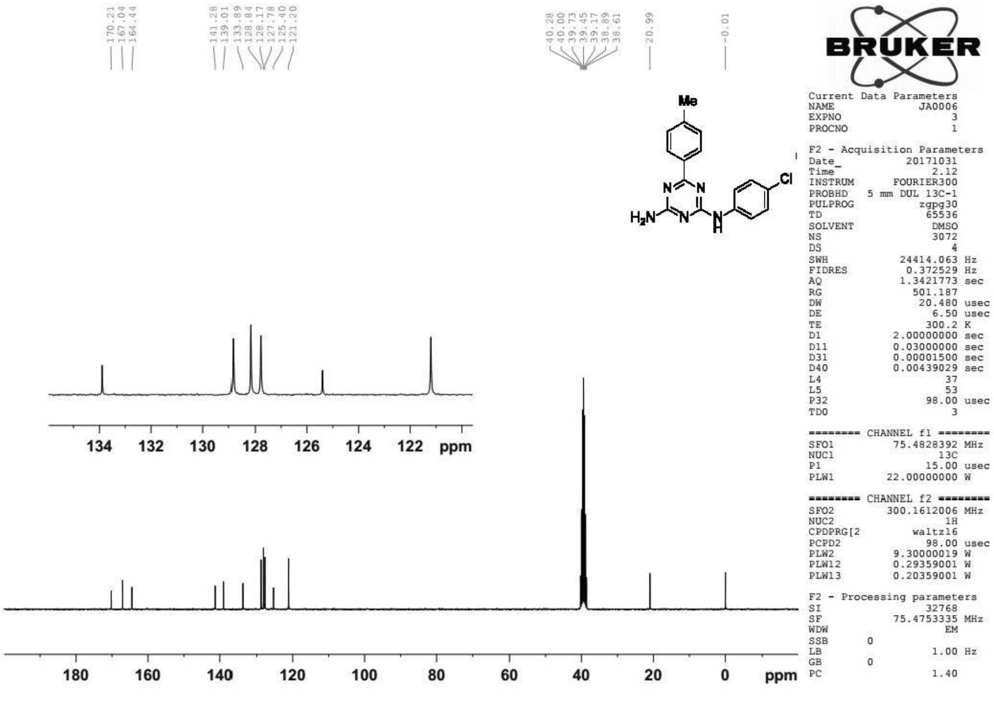

6-(4-Methoxyphenyl)-N²-phenyl-1,3,5-triazine-2,4-diamine (8)

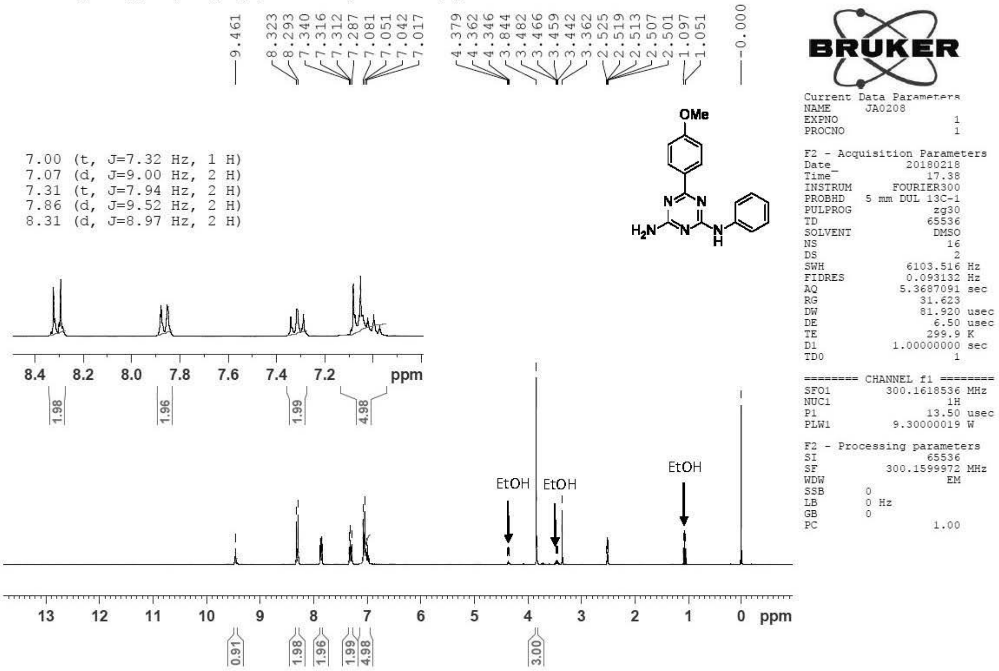

6-(4-Methoxyphenyl)-N²-phenyl-1,3,5-triazine-2,4-diamine (8)

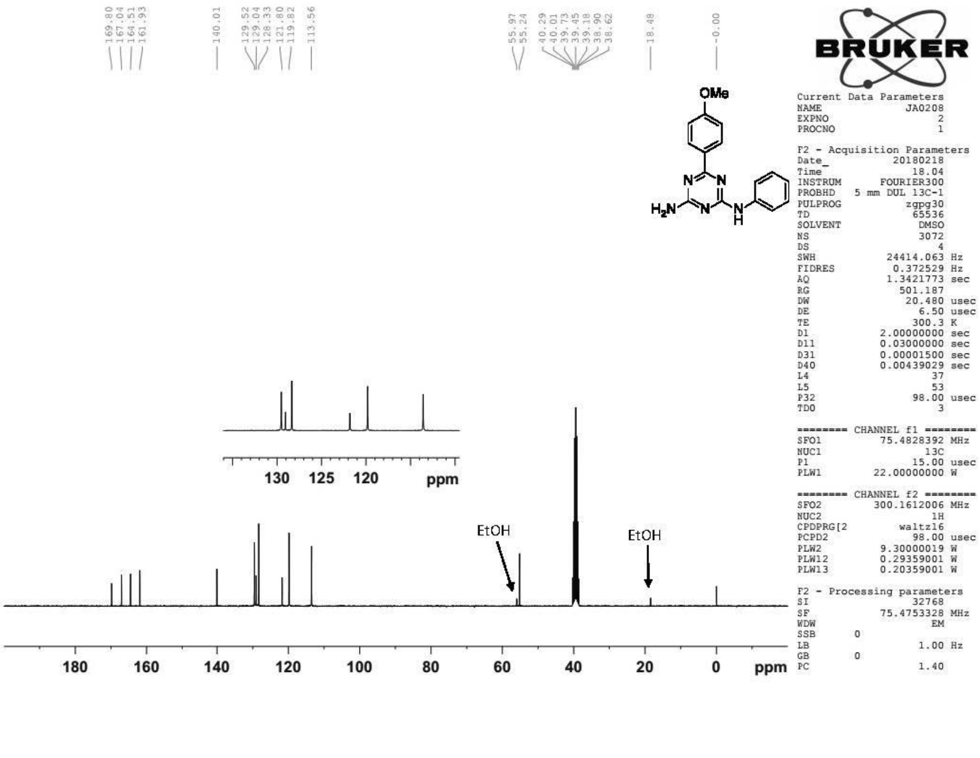

6-(4-Methoxyphenyl)-N²-(p-tolyl)-1,3,5-triazine-2,4-diamine (9)

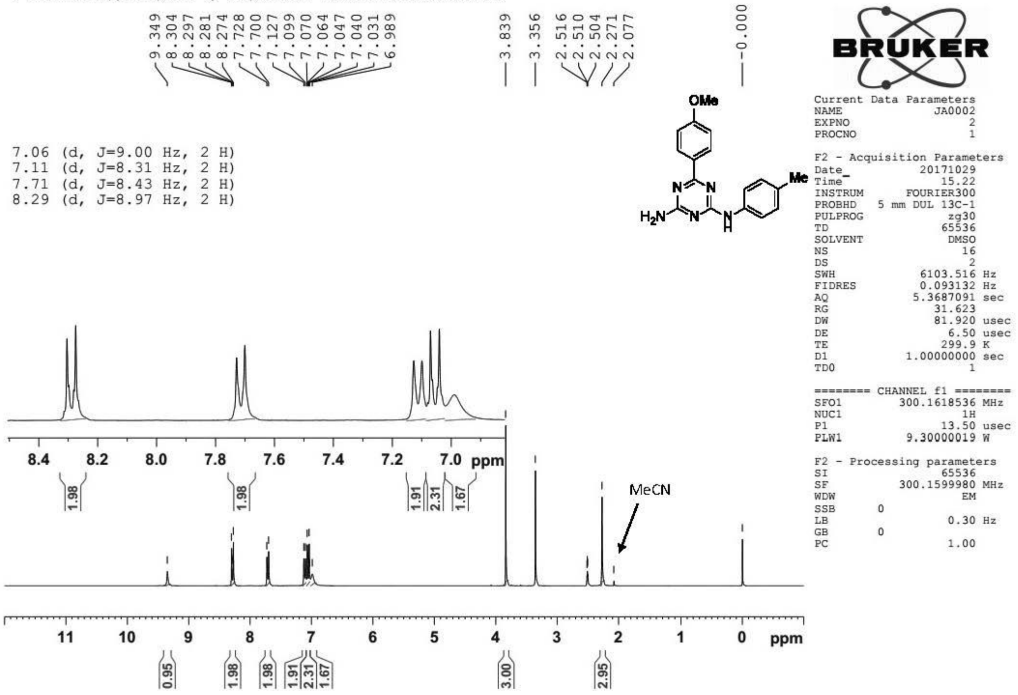

6-(4-Methoxyphenyl)-N²-(p-tolyl)-1,3,5-triazine-2,4-diamine (9)

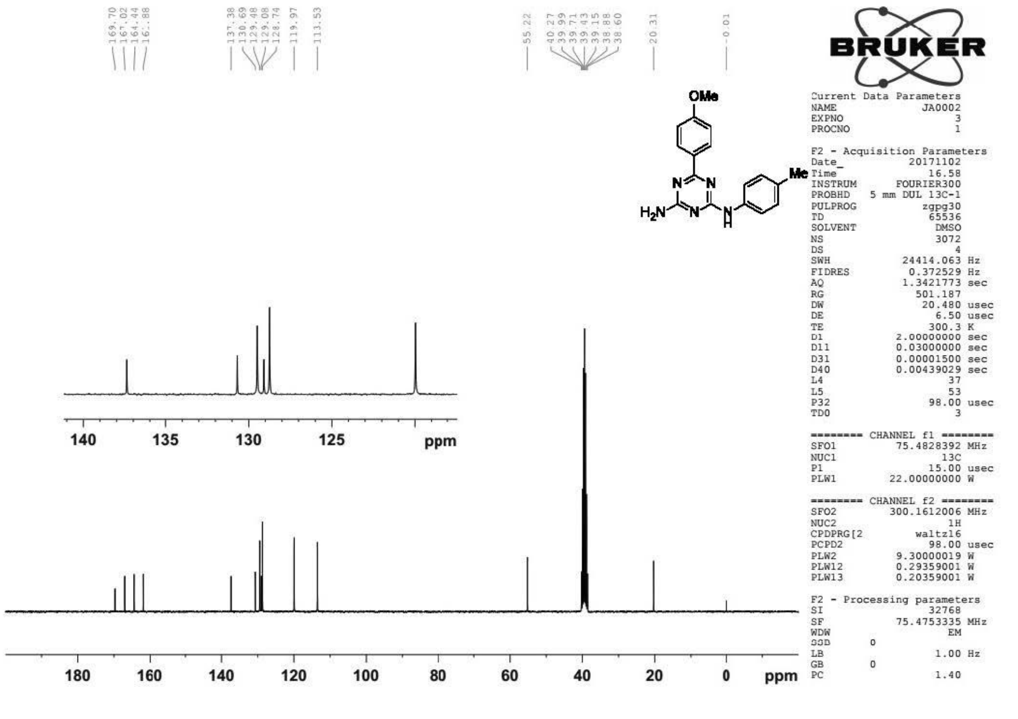

N^2-Phenyl-6-(3,4,5-trimethoxyphenyl)-1,3,5-triazine-2,4-diamine (10)

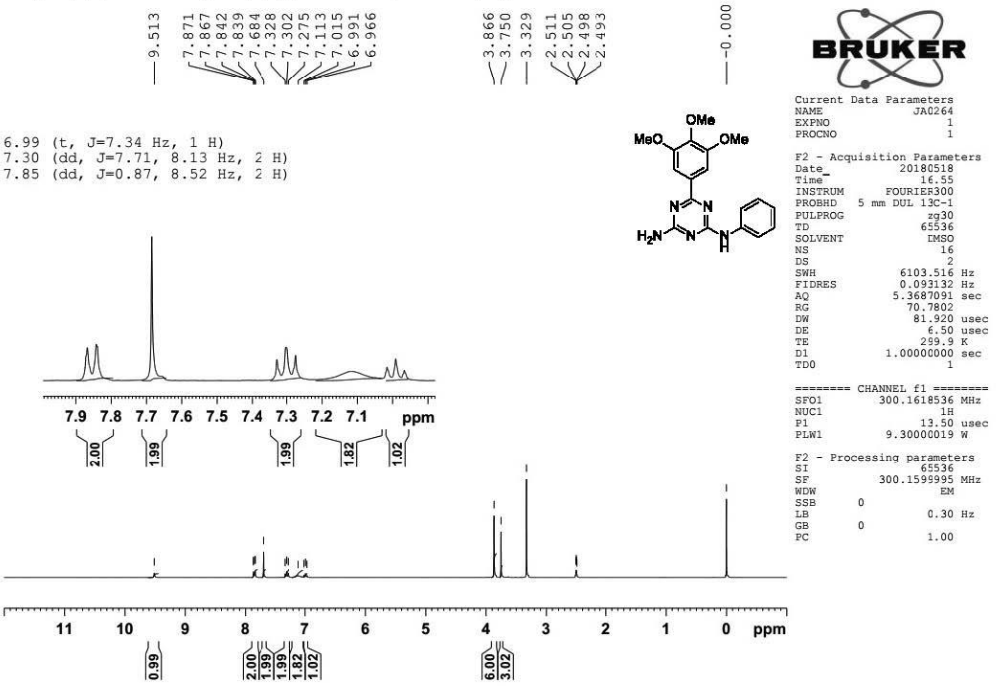

N^2-Phenyl-6-(3,4,5-trimethoxyphenyl)-1,3,5-triazine-2,4-diamine (10)

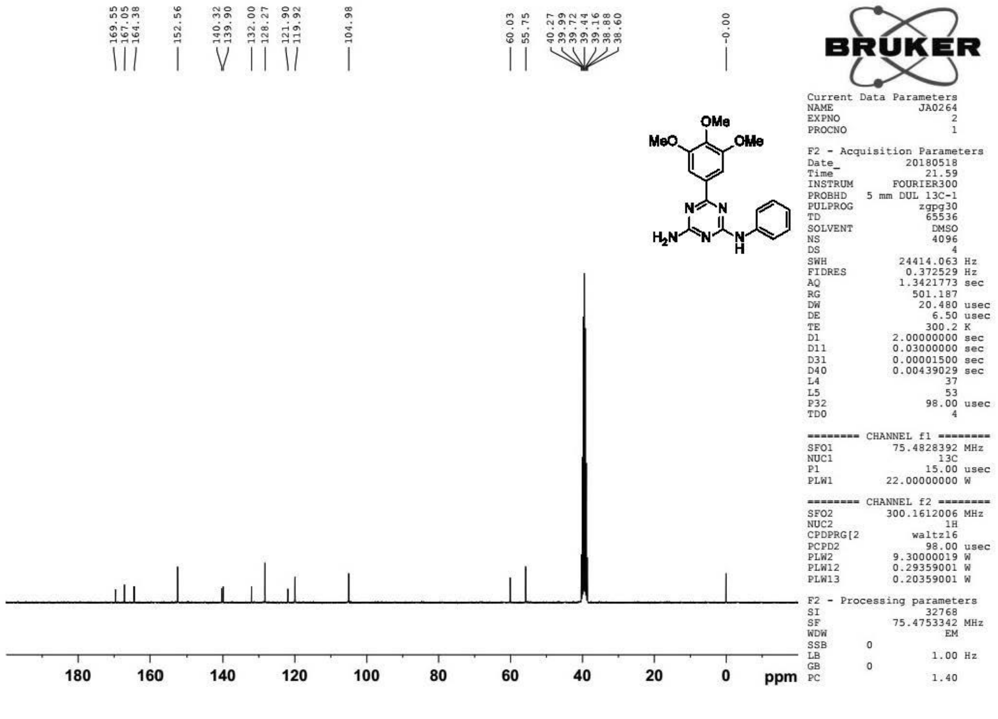

N²-(2-Fluorophenyl)-6-(3,4,5-trimethoxyphenyl)-1,3,5-triazine-2,4-diamine (11)

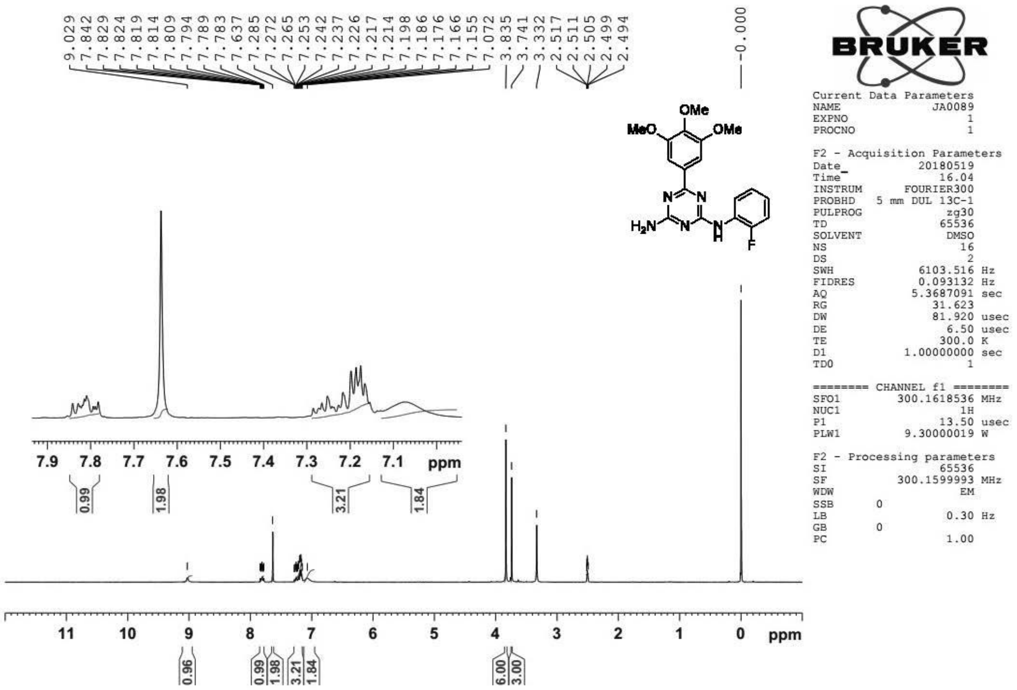

N²-(2-Fluorophenyl)-6-(3,4,5-trimethoxyphenyl)-1,3,5-triazine-2,4-diamine (11)

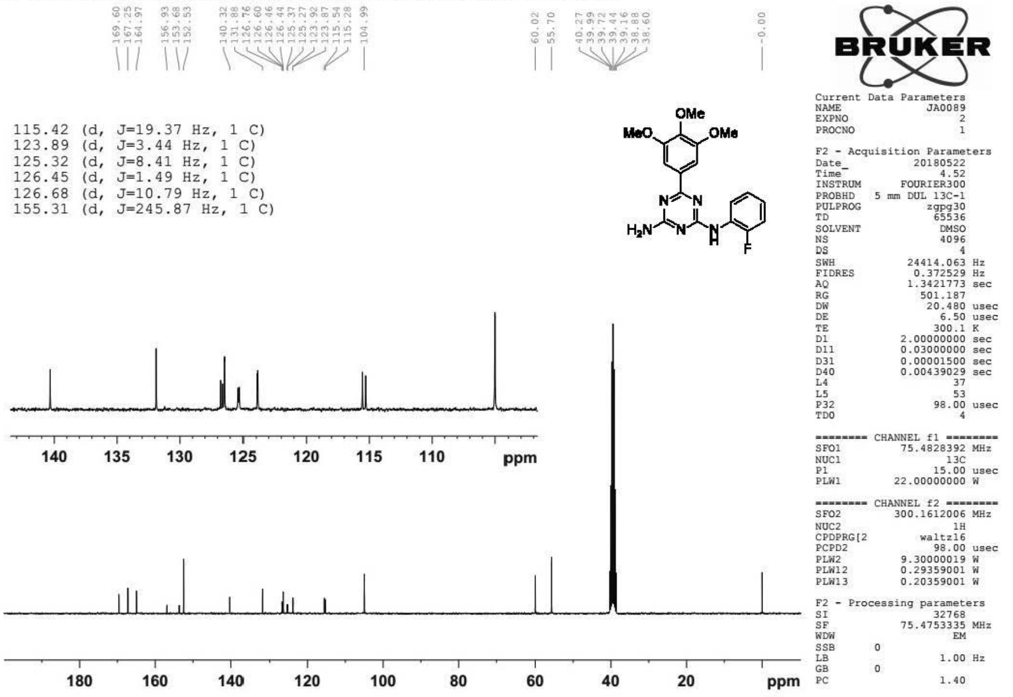

N[2]-(2-Chlorophenyl)-6-(3,4,5-trimethoxyphenyl)-1,3,5-triazine-2,4-diamine (12)

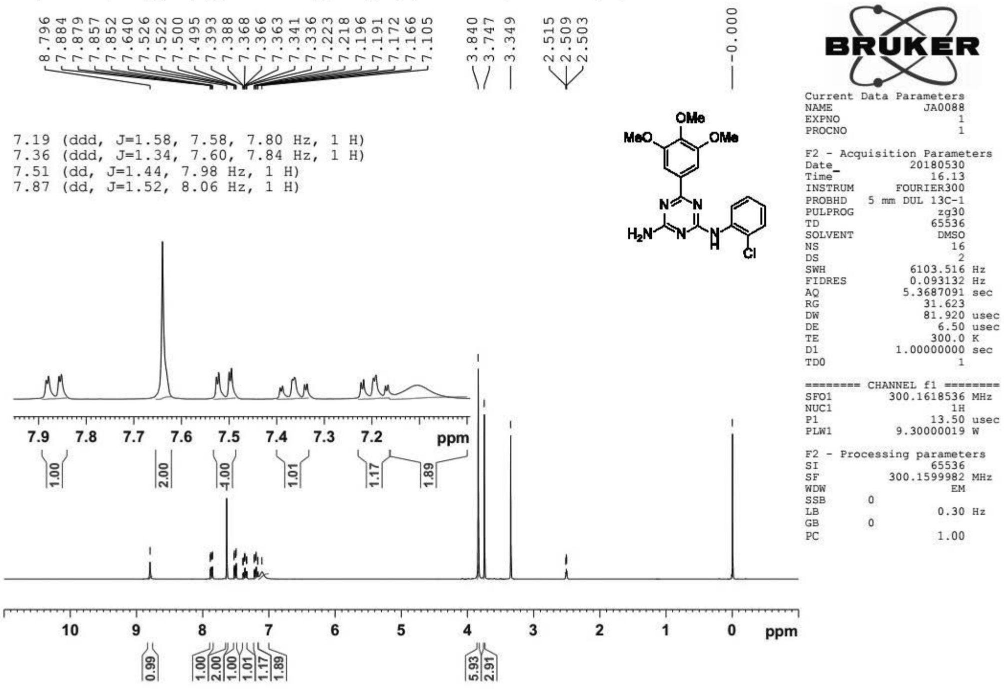

N[2]-(2-Chlorophenyl)-6-(3,4,5-trimethoxyphenyl)-1,3,5-triazine-2,4-diamine (12)

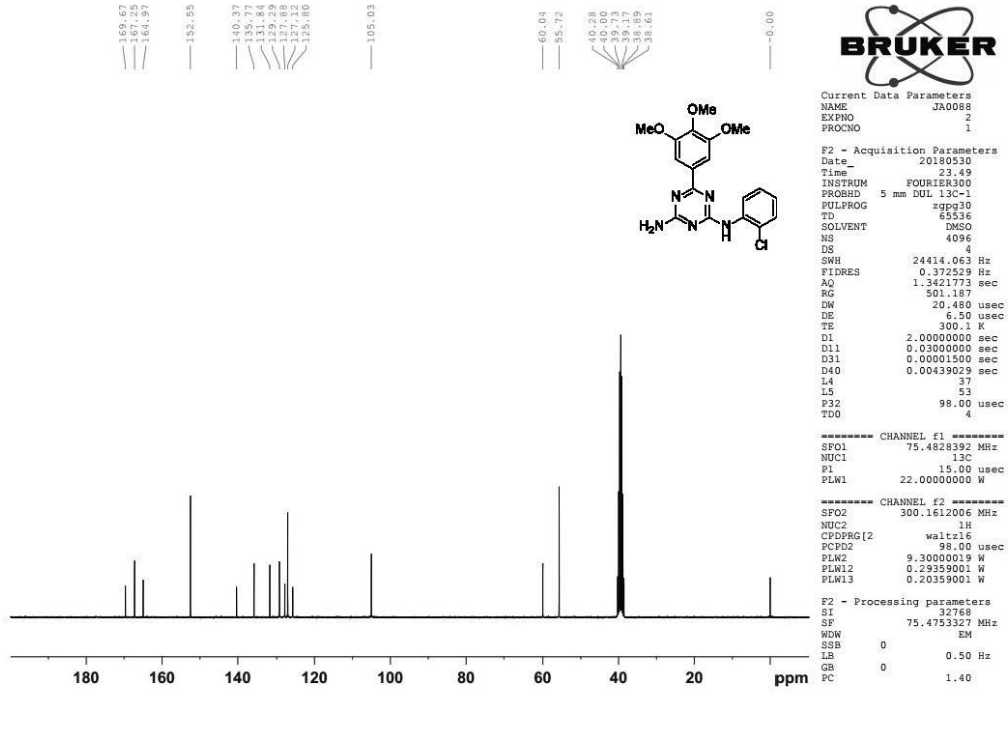

N[2]-(2-Methoxyphenyl)-6-(3,4,5-trimethoxyphenyl)-1,3,5-triazine-2,4-diamine (13)*

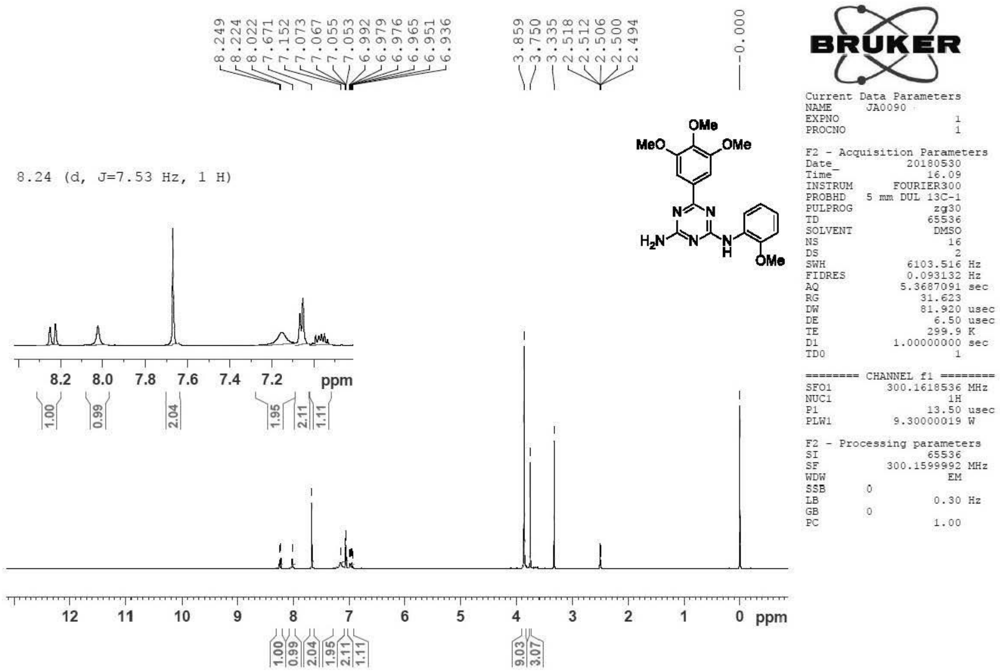

N[2]-(2-Methoxyphenyl)-6-(3,4,5-trimethoxyphenyl)-1,3,5-triazine-2,4-diamine (13)*

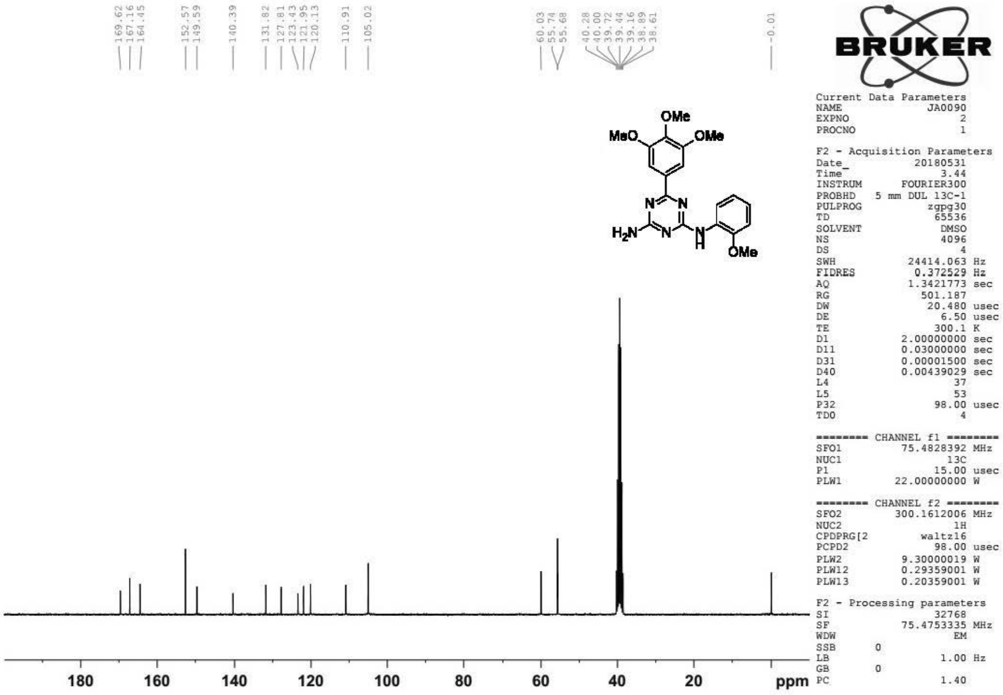

N^2-(3-Chlorophenyl)-6-(3,4,5-trimethoxyphenyl)-1,3,5-triazine-2,4-diamine (14)

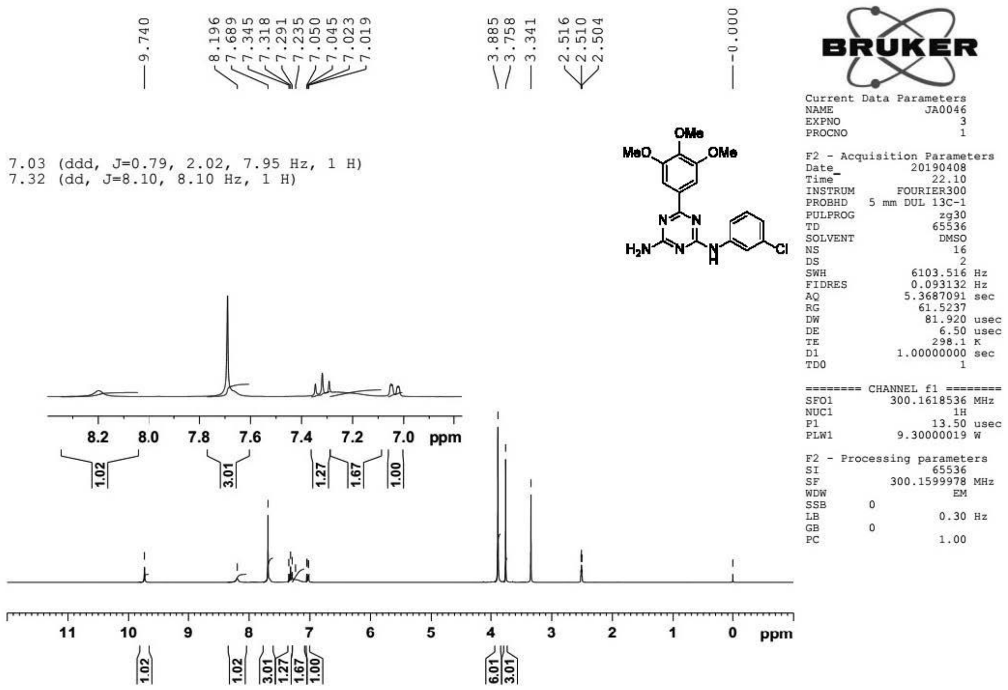

N^2-(3-Chlorophenyl)-6-(3,4,5-trimethoxyphenyl)-1,3,5-triazine-2,4-diamine (14)

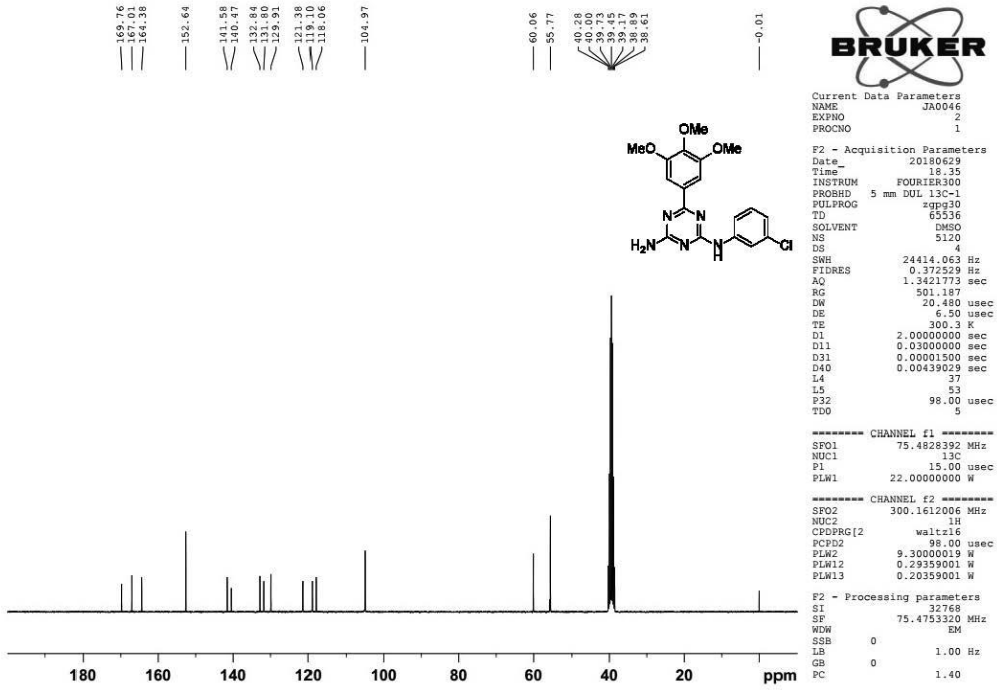

N²-(m-Tolyl)-6-(3,4,5-trimethoxyphenyl)-1,3,5-triazine-2,4-diamine (15)

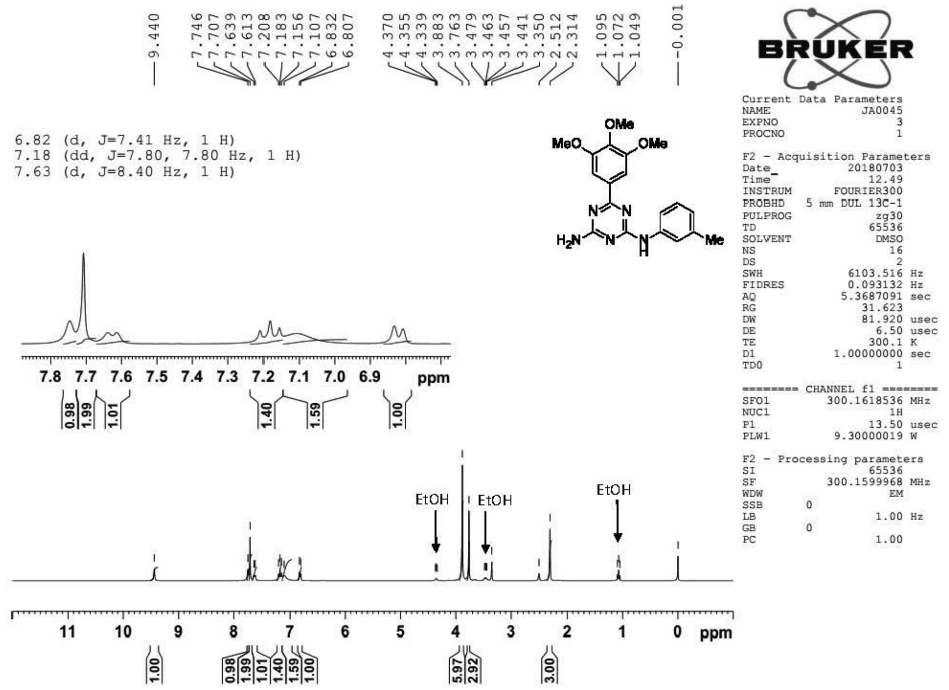

N²-(m-Tolyl)-6-(3,4,5-trimethoxyphenyl)-1,3,5-triazine-2,4-diamine (15)

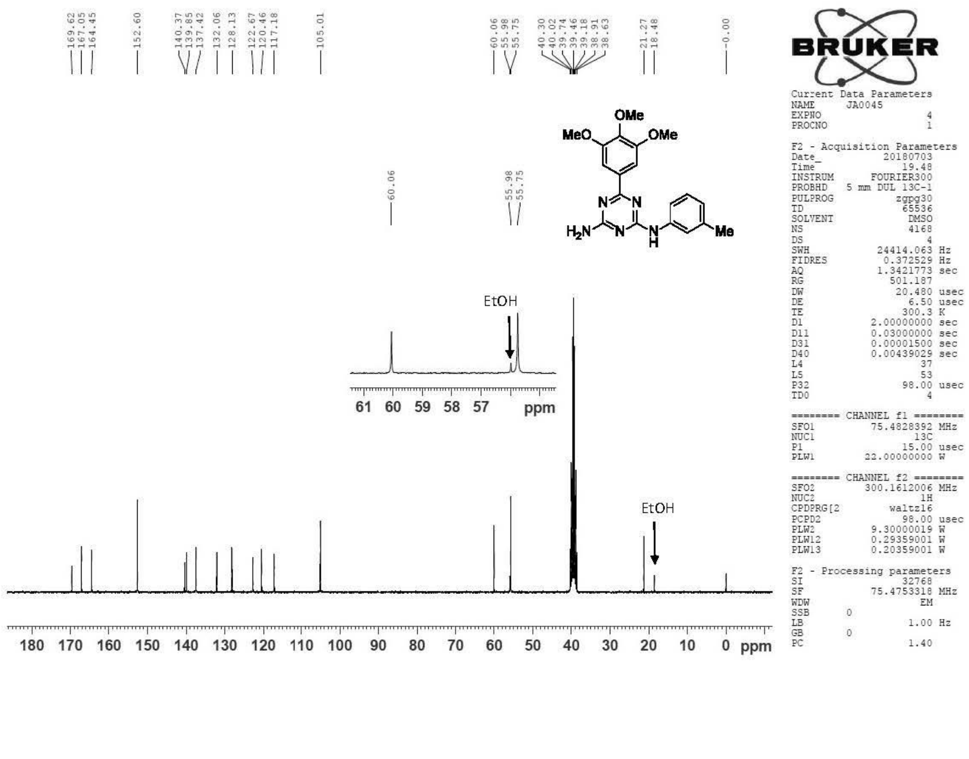

N[2]-(4-Chlorophenyl)-6-(3,4,5-trimethoxyphenyl)-1,3,5-triazine-2,4-diamine (16)

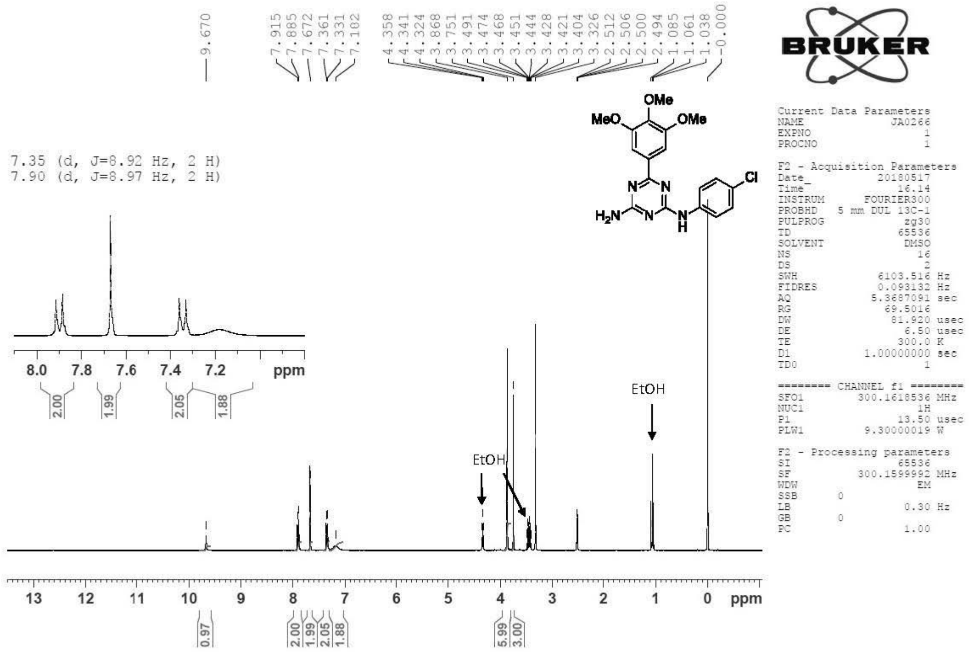

N[2]-(4-Chlorophenyl)-6-(3,4,5-trimethoxyphenyl)-1,3,5-triazine-2,4-diamine (16)

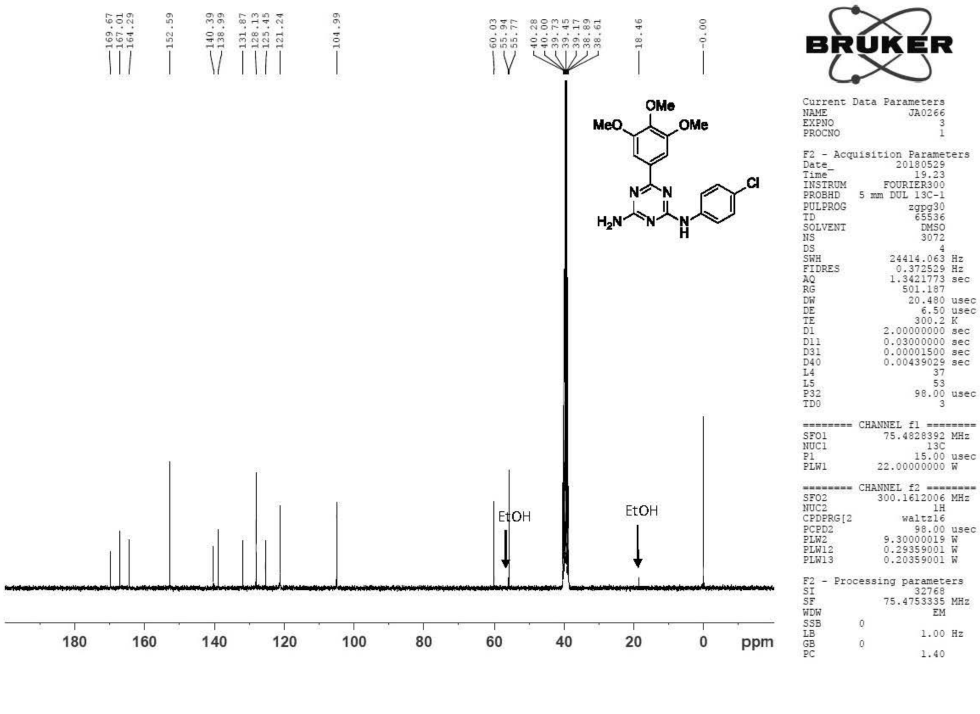

N²-(4-Bromophenyl)-6-(3,4,5-trimethoxyphenyl)-1,3,5-triazine-2,4-diamine (17)

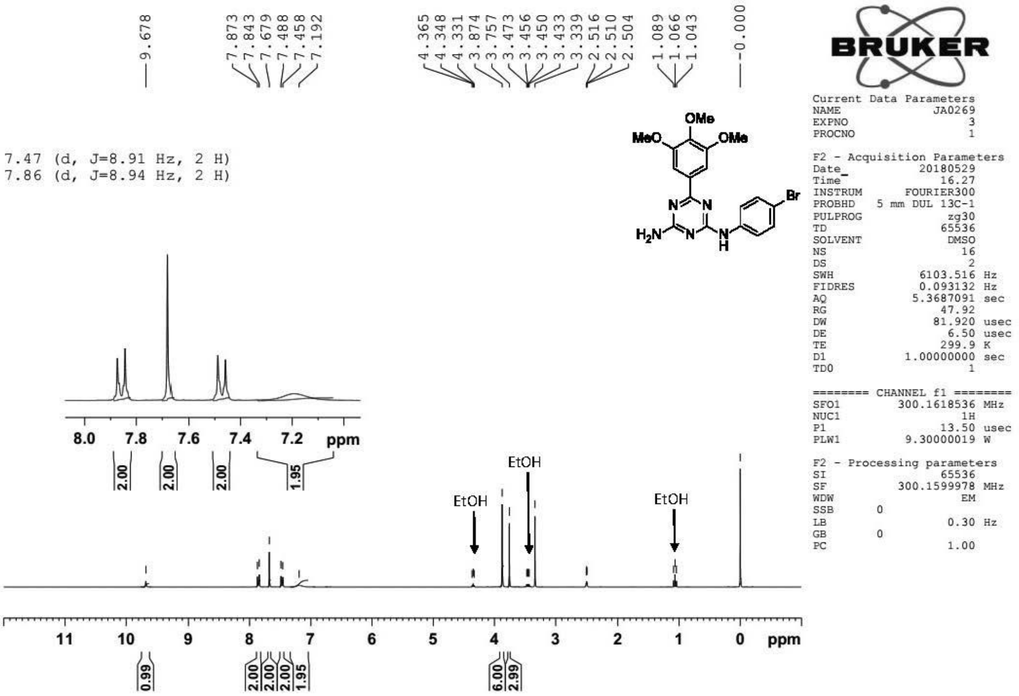

N²-(4-Bromophenyl)-6-(3,4,5-trimethoxyphenyl)-1,3,5-triazine-2,4-diamine (17)

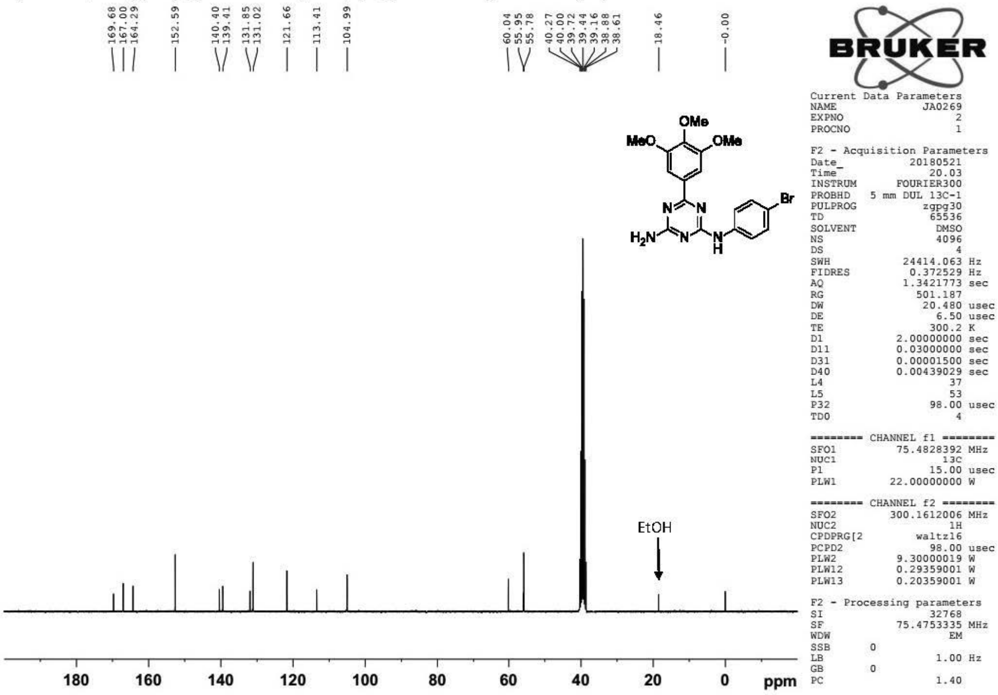

N²-(p-Tolyl)-6-(3,4,5-trimethoxyphenyl)-1,3,5-triazine-2,4-diamine (18)

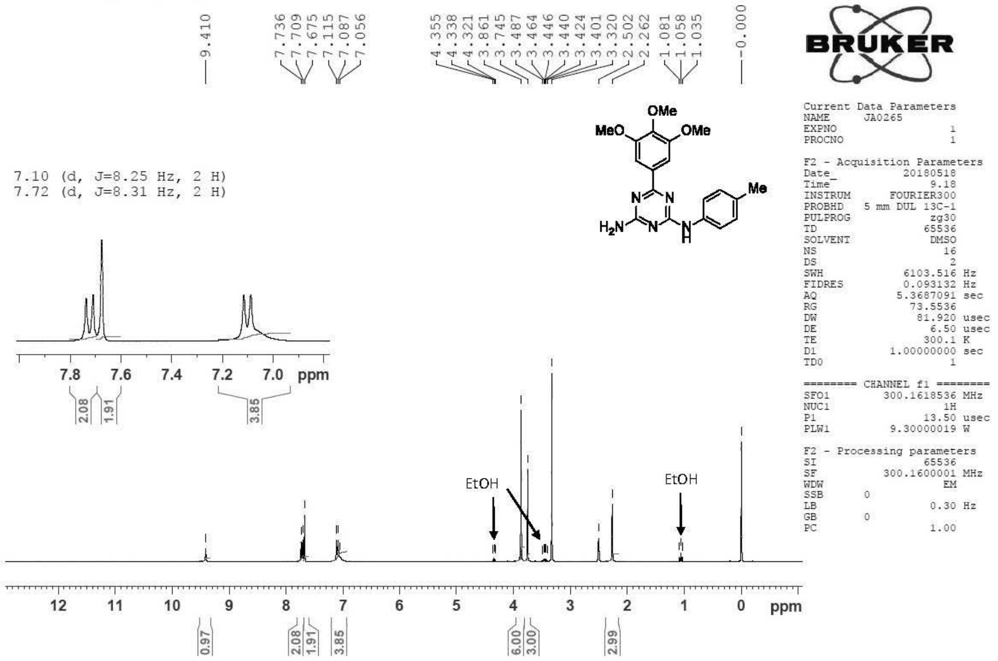

N²-(p-Tolyl)-6-(3,4,5-trimethoxyphenyl)-1,3,5-triazine-2,4-diamine (18)

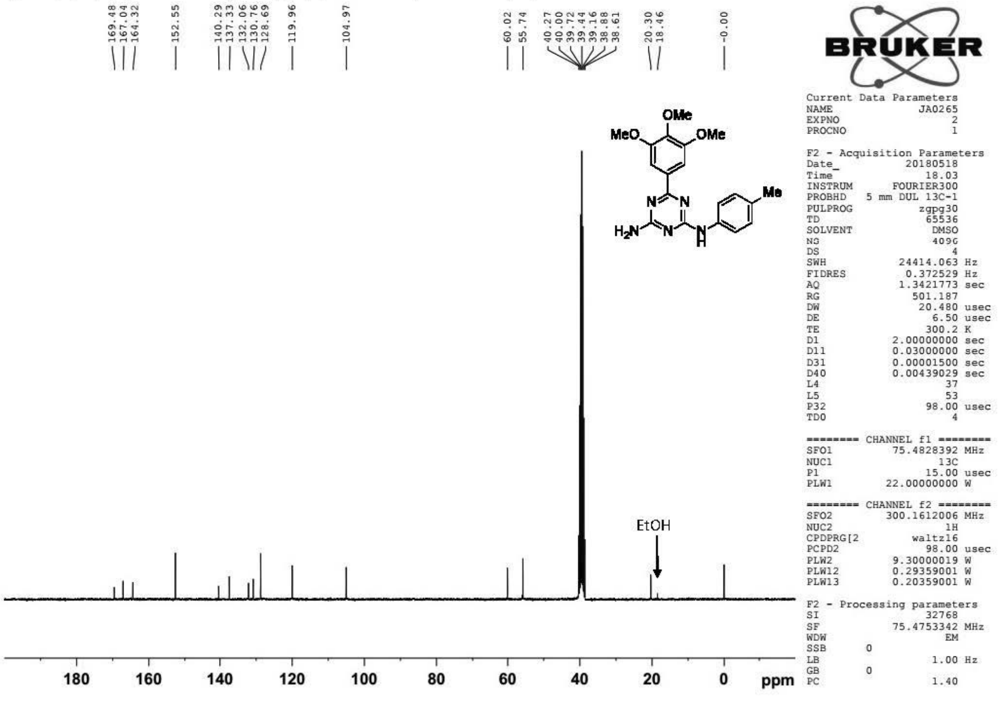

N[2]-(4-Methoxyphenyl)-6-(3,4,5-trimethoxyphenyl)-1,3,5-triazine-2,4-diamine (19)*

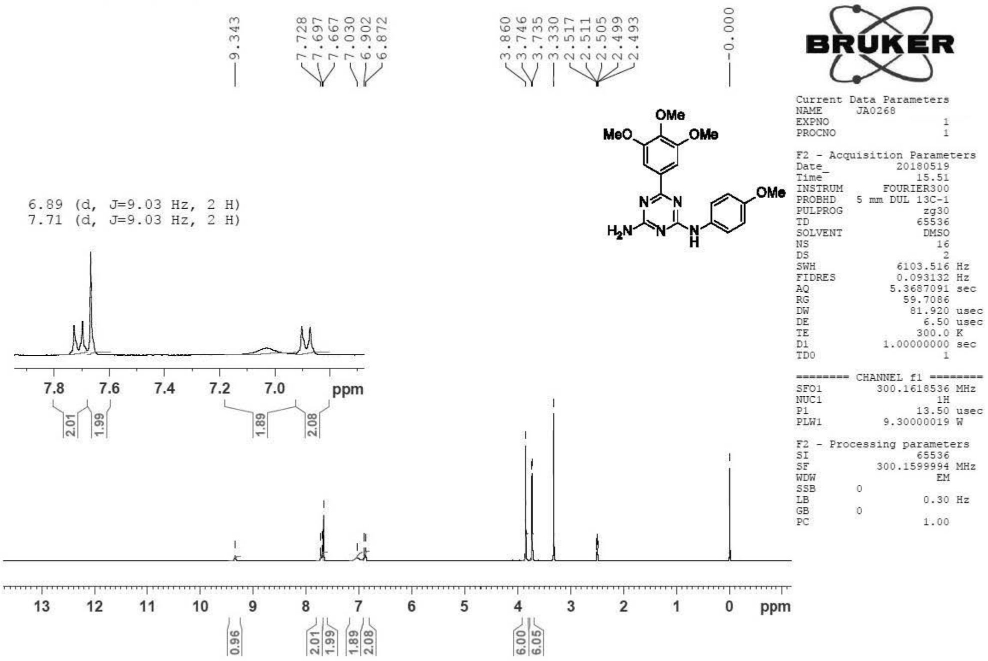

N[2]-(4-Methoxyphenyl)-6-(3,4,5-trimethoxyphenyl)-1,3,5-triazine-2,4-diamine (19)*

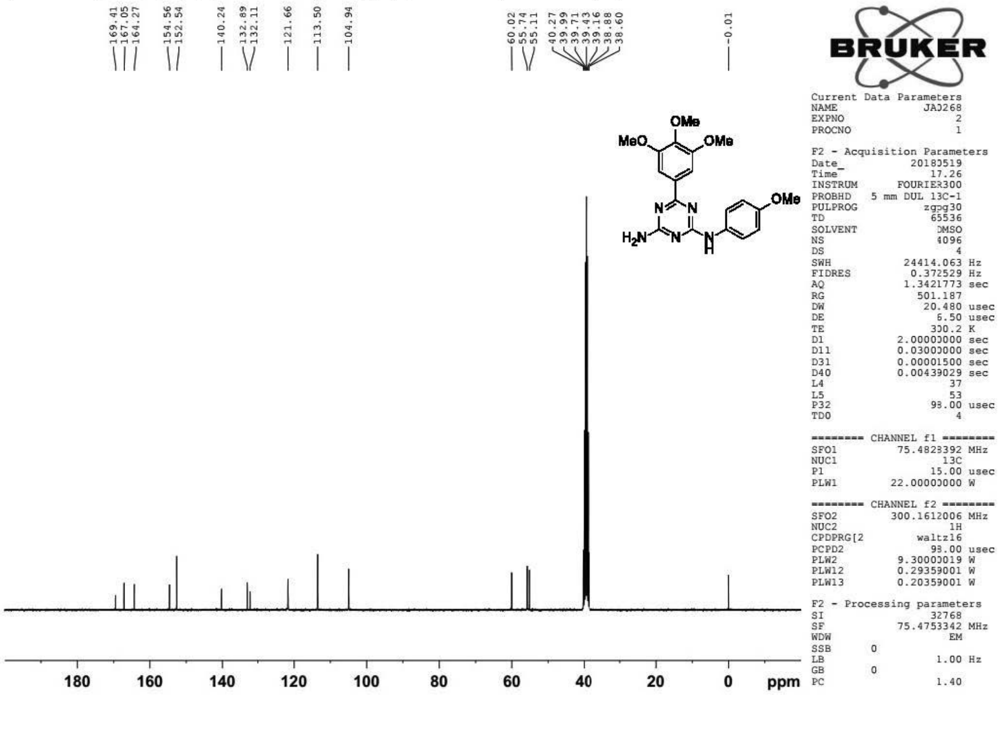

N²-(4-(Trifluoromethoxy)phenyl)-6-(3,4,5-trimethoxyphenyl)-1,3,5-triazine-2,4-diamine (20)

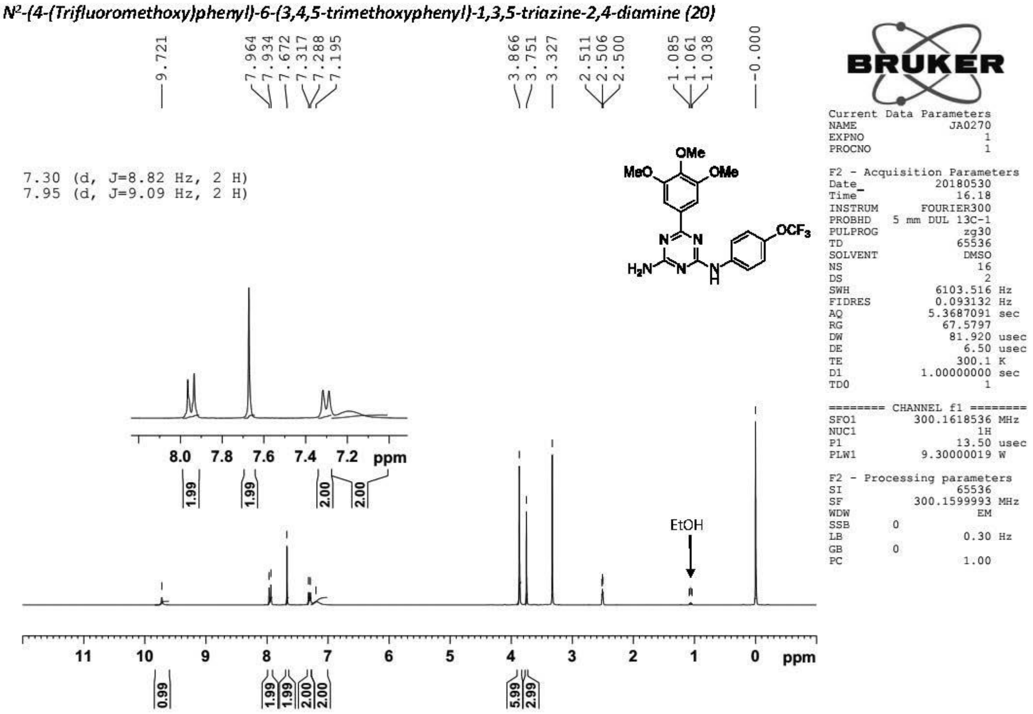

N²-(4-(Trifluoromethoxy)phenyl)-6-(3,4,5-trimethoxyphenyl)-1,3,5-triazine-2,4-diamine (20)

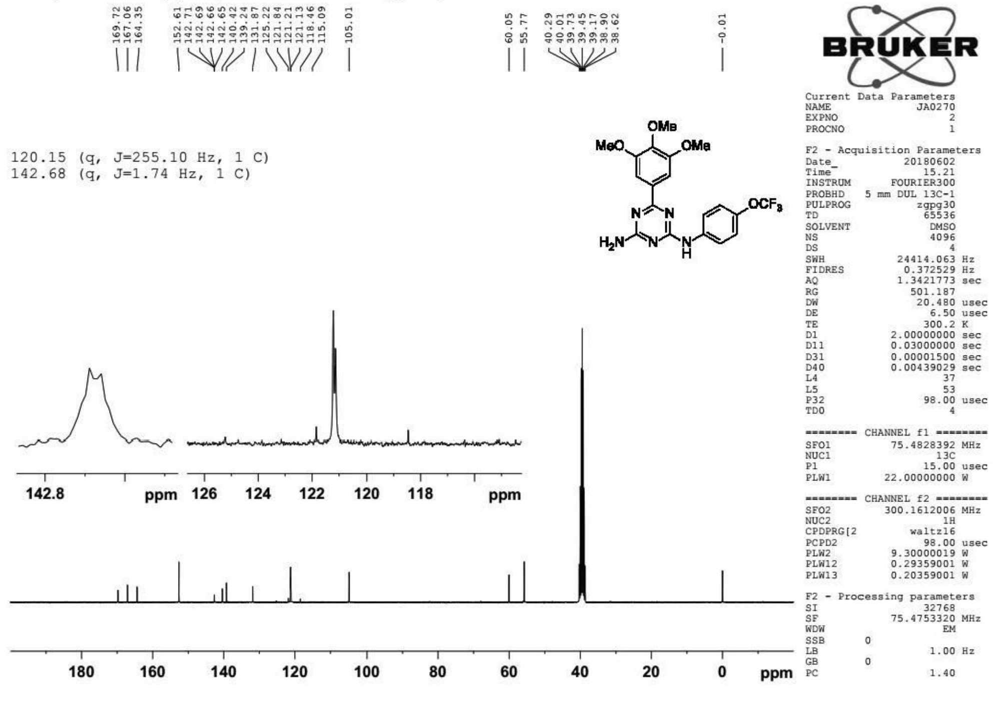

N[2]-(4-Isopropylphenyl)-6-(3,4,5-trimethoxyphenyl)-1,3,5-triazine-2,4-diamine (21)

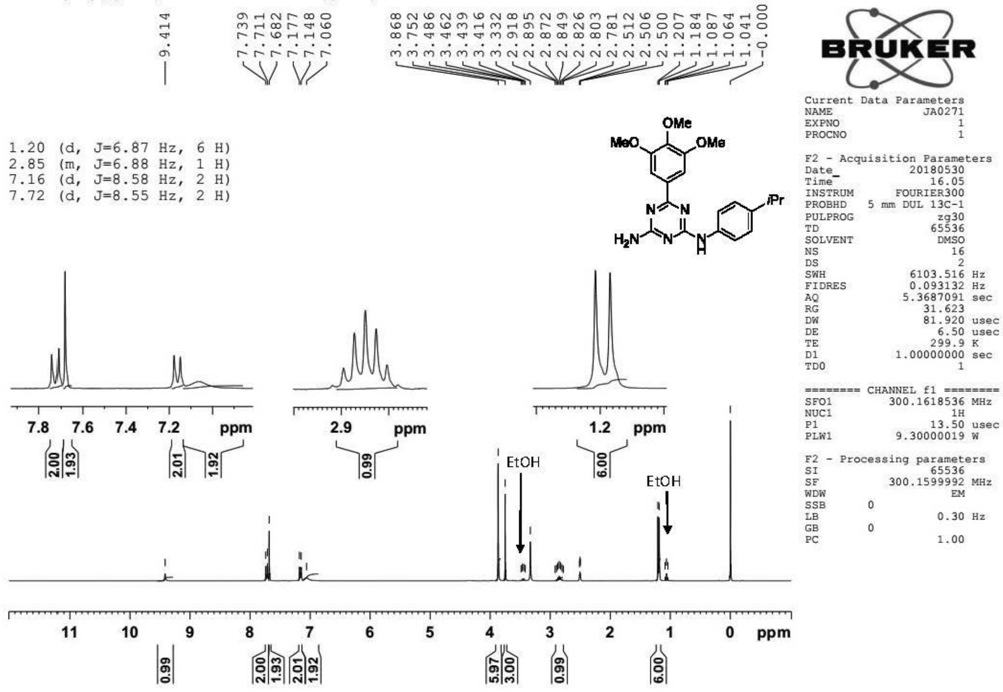

N[2]-(4-Isopropylphenyl)-6-(3,4,5-trimethoxyphenyl)-1,3,5-triazine-2,4-diamine (21)

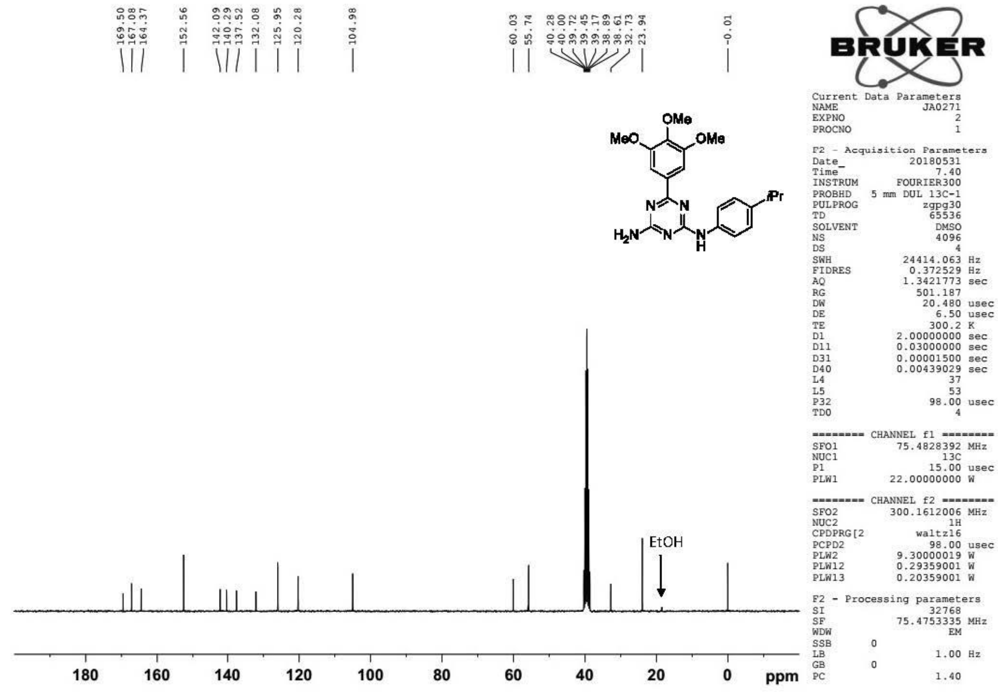

X-ray structure analysis of
N^2-(4-(trifluoromethoxy)phenyl)-6-(3,4,5-trimethoxyphenyl)-1,3,5-triazine-2,4-diamine (20)

The molecular structure of **20**, being representative of the series, has been established by single crystal X-crystallography and is illustrated in Figure S1. The triazine ring is strictly planar with the r.m.s. deviation of the six atoms comprising the plane being 0.0059 Å. The C–N bond lengths in the triazine ring lie within the relatively narrow range 1.331(3) Å, for C1–N3, to 1.354(3) Å, for C3–N3, suggesting substantial delocalisation of π-electron density over the ring. The appended N4, N5 and C4 atoms are close to co-planar with the ring lying, respectively, -0.039(3), 0.049(3) and 0.012(3) Å, out of the plane of the ring. The dihedral angle formed between the central ring and the methoxy-substituted ring of 2.30(8)° is consistent with a co-planar relationship, and the dihedral angle between the triazine and N5-bound rings of 11.93(7) indicates an inclined relationship. Overall, to a first approximation, the molecule is considered planar as seen in the dihedral angle between the outer rings of 10.84(7)°. The peripheral rings are orientated to the same side of the molecule, so the molecule has the shape of the letter U. The methoxy substituents adopt different conformations in order to minimise steric strain. Thus, the central methoxy group lies out of the plane of the ring to which it is connected with the C11–O2–C7–C8 torsion angle being 81.7(2)° whereas the flanking methoxy groups are co-planar with the ring: C10–O1–C6–C7 is 179.57(18)° and C12–O3–C8–C7 is 177.66(18)°. The trifluoromethoxy group lies out of the plane of the ring to which it is connected as seen in the C19–O4–C16–C15 torsion angle of 77.9(3)°.

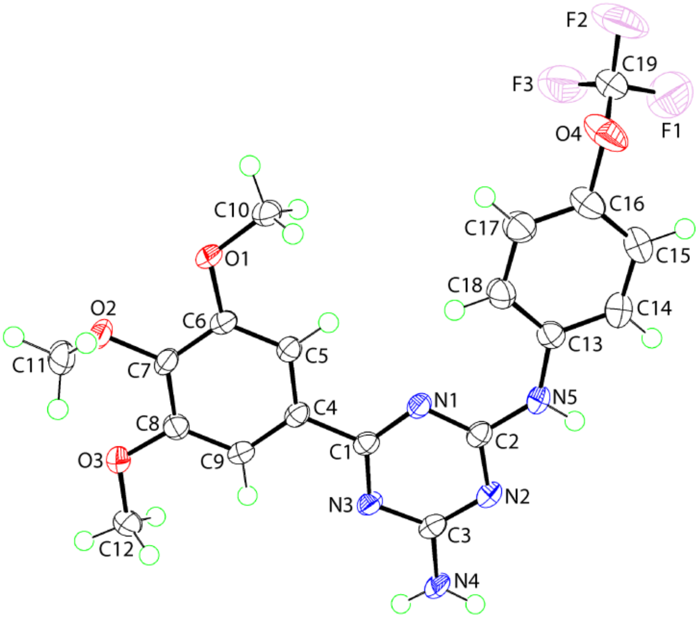

Figure S1. Molecular structure of **20**, showing atom labelling scheme and anisotropic displacement parameters at the 70% probability level.

In the crystal of **20**, the primary-amine forms a hydrogen bond with a methoxy-oxygen atom to sustain a linear supramolecular chain along the *b*-axis, as shown in Figure S2(a); geometric parameters characterising the intermolecular interactions are collated in the figure caption. The two remaining acidic hydrogen atoms do not

participate in structure-directing intermolecular interactions. A supramolecular layer in the ac-plane is formed through the cooperativity of π···π interactions between the triazine-(N1-N3,C1-C3) and appended phenyl-(C4-C9) rings along with methyl-C–H···π(C13-C18) interactions. Layer stack along the *b*-axis with no directional interactions between them.

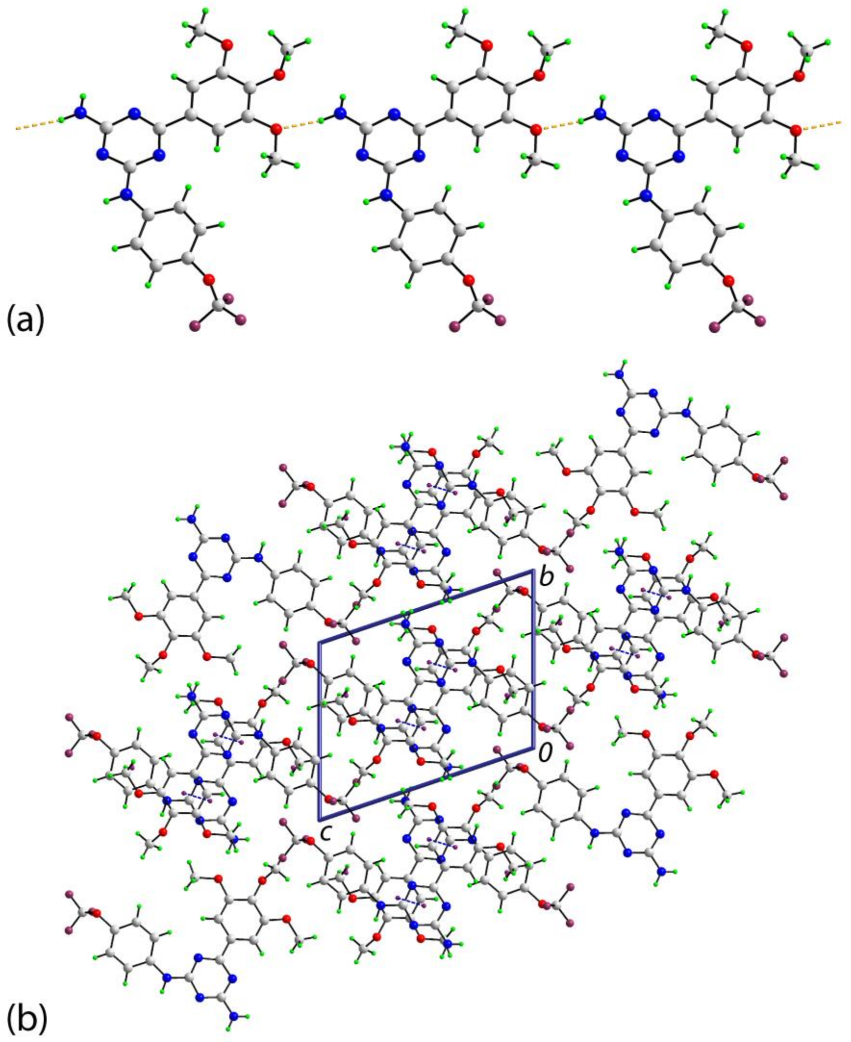

Figure S2. Molecular packing in the crystal of **20**:

(a) supramolecular linear chain sustained by amine-N–H···O(methoxy) hydrogen bonds (shown as orange dashed lines) [N4–H2n···O1^{i}: H2n···O1^{i} = 2.08(3) Å, N4···O1^{i} = 2.941(3) Å and angle at H2n= 164(3)° for symmetry operation (i) x, 1+y, z];

(b) a view of the unit contents in projection down the a-axis. The π···π [Cg(triazine) ···Cg(trimethoxyphenyl)^{ii} = 3.5987(13) Å and angle of inclination = 2.30(11)° for symmetry operation (ii) 2-x, 1-y, 1-z] and methyl-C–H···π [C12–H12a···Cg(trifluoromethoxyphenyl)^{iii} = 2.88 Å, C12···Cg(trifluoromethoxyphenyl)^{iii} = 3.461(3) Å with angle at H12a = 119° and C12–H12c···Cg(trifluoromethoxyphenyl)^{iv} = 2.90 Å, C12 ···Cg(trifluoromethoxyphenyl)^{iv} = 3.821(3) Å with angle at H12c = 157° for symmetry operations (iii) 2-x, 1-y, 1-z and (iv) 1-x, 1-y, 1-z] interactions are shown as blue and purple dashed lines, respectively.

Concentration-response curves for the compounds tested against MDA-MB231 breast cancer cells

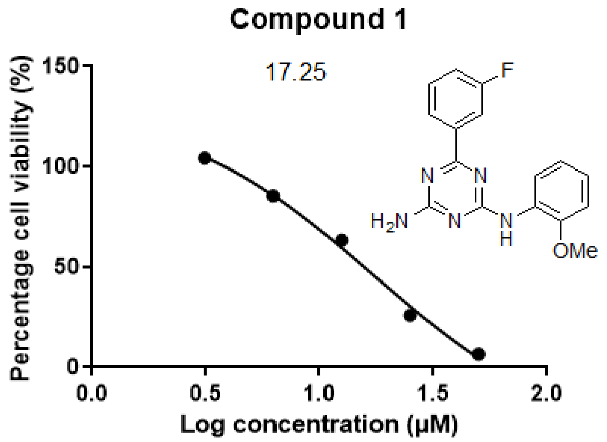

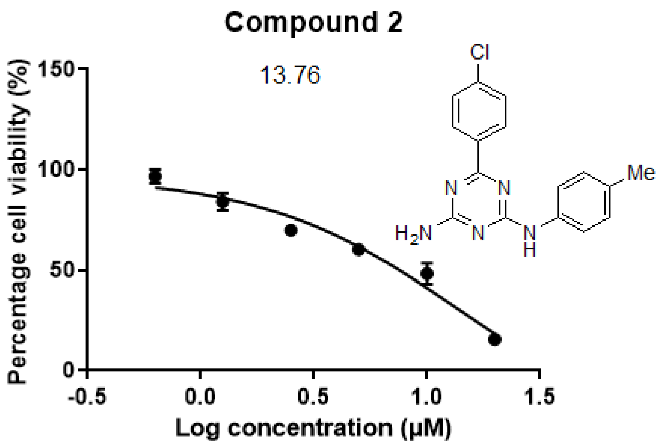

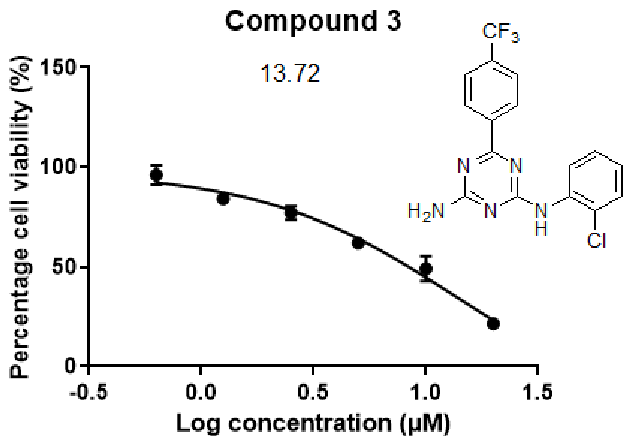

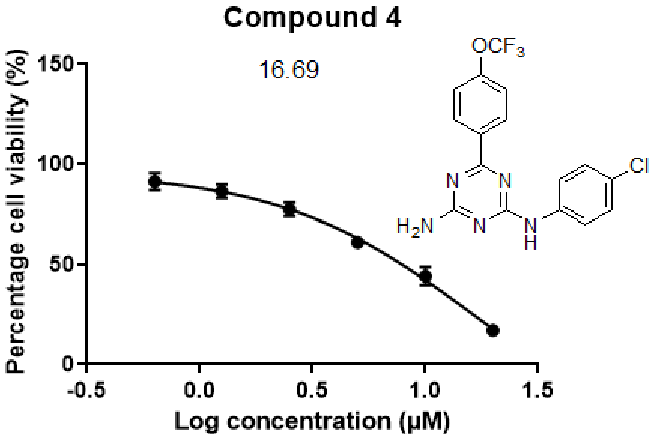

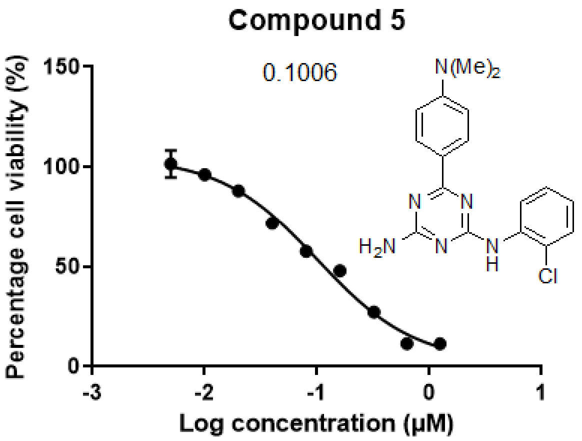

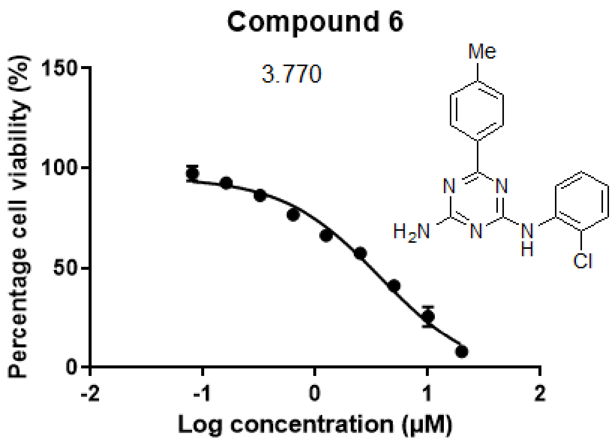

Compound 7

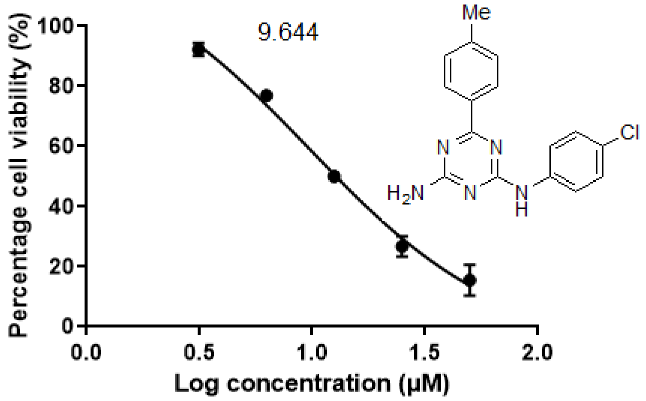

Compound 8

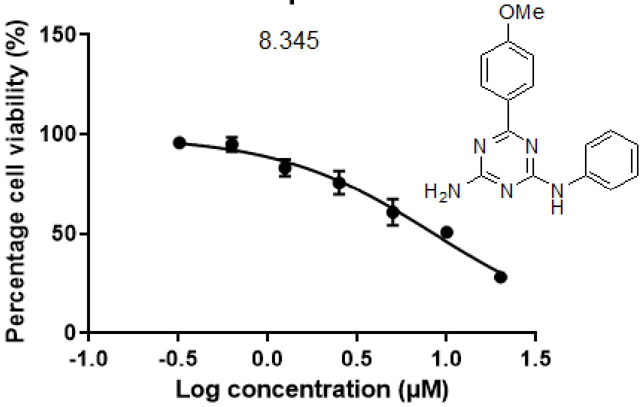

Compound 9

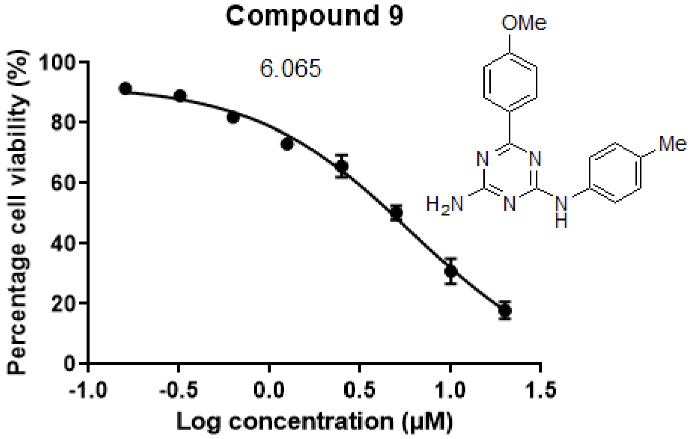

Compound 10

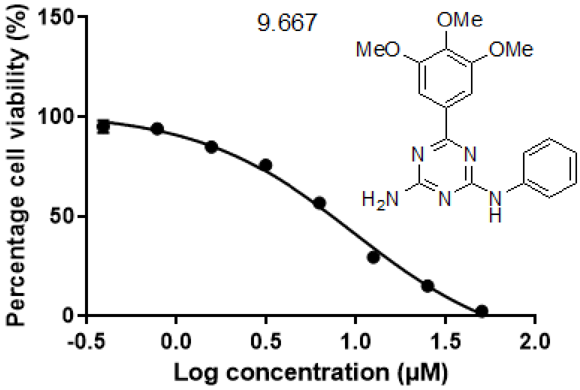

Compound 11

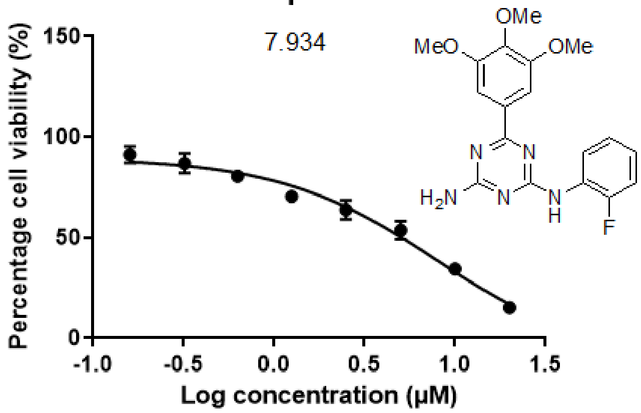

Compound 12

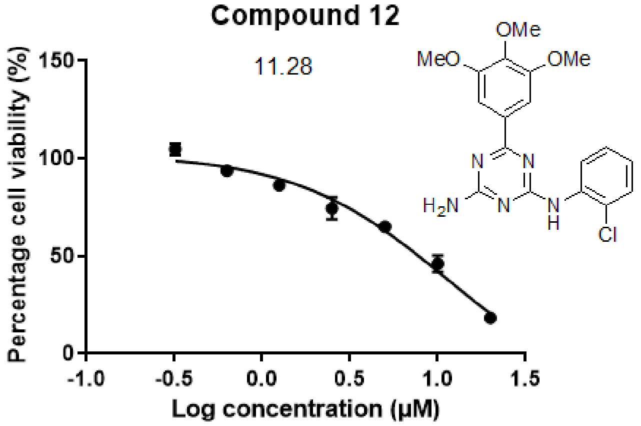

Compound 13

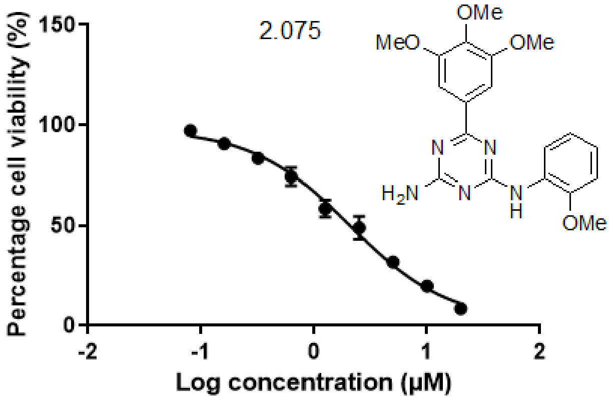

Compound 14

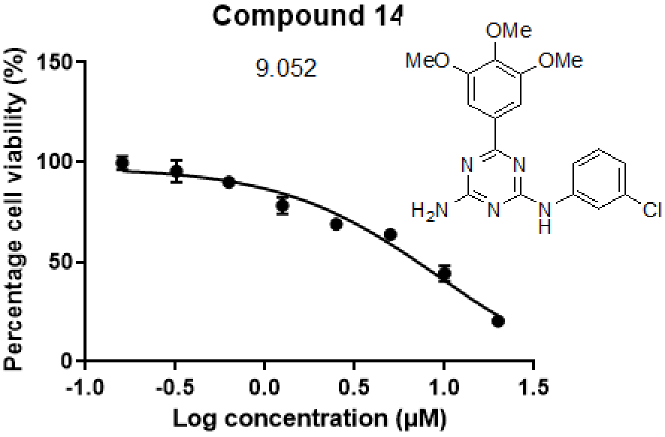

Compound 15

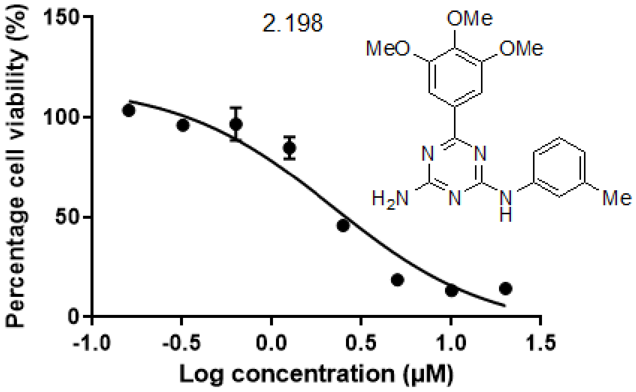

Compound 16

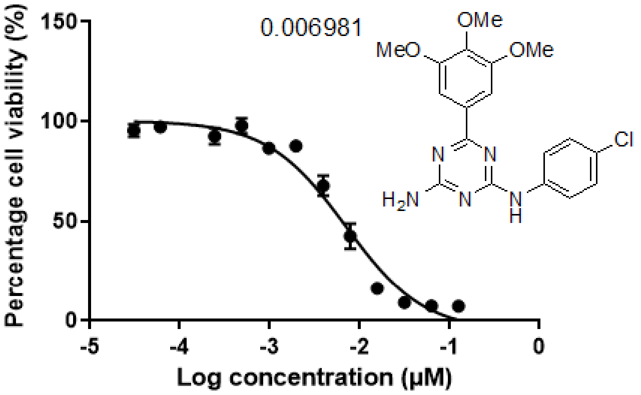

Compound 17

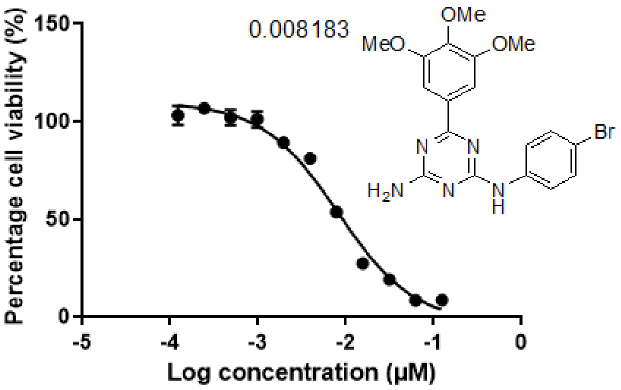

Compound 18

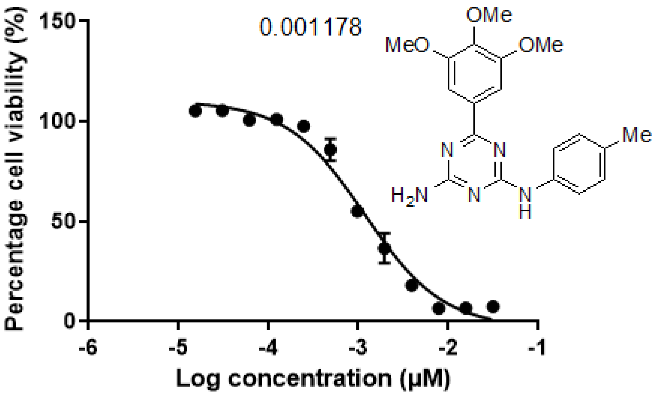

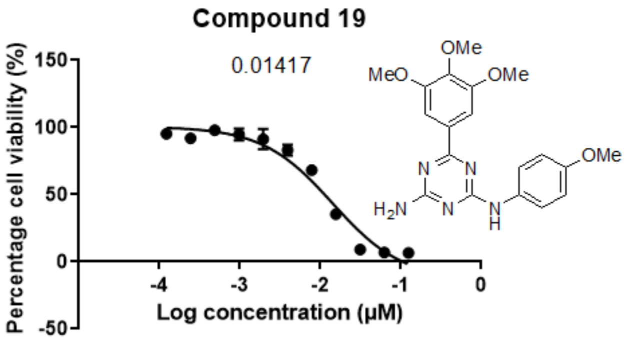
Compound 19
0.01417
Percentage cell viability (%)
Log concentration (μM)
MeO
OMe
OMe
OMe
N
N
N
H2N
N
H

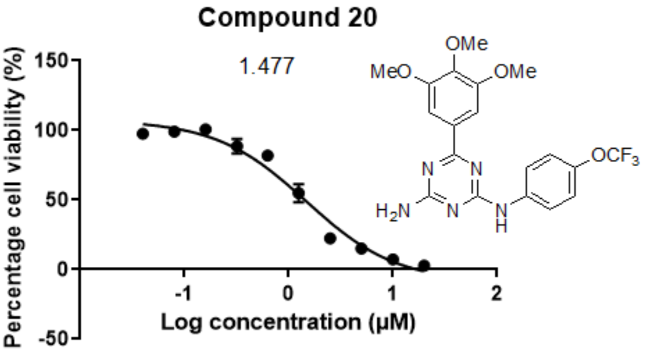
Compound 20
1.477
Percentage cell viability (%)
Log concentration (μM)
MeO
OMe
OMe
OCF3
H2N
N
H

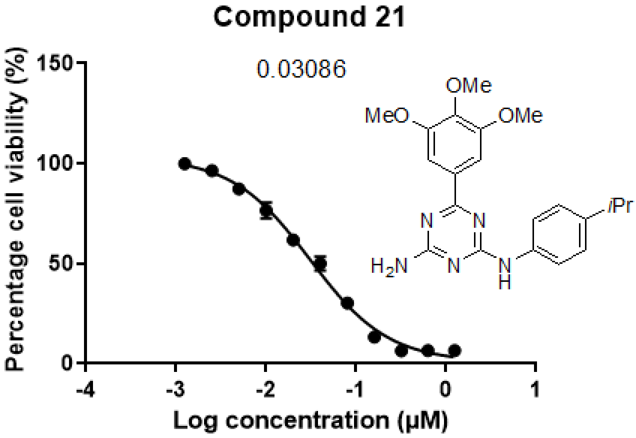
Compound 21
0.03086
Percentage cell viability (%)
Log concentration (μM)
MeO
OMe
OMe
iPr
H2N
N
H

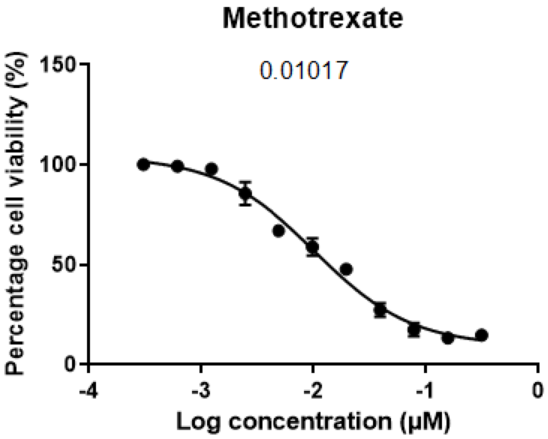
Methotrexate
0.01017
Percentage cell viability (%)
Log concentration (μM)

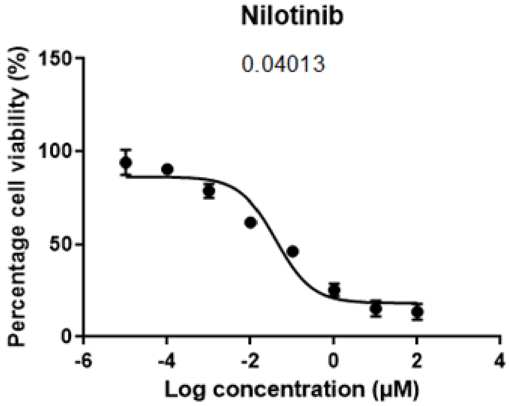
Nilotinib
0.04013
Percentage cell viability (%)
Log concentration (μM)

Concentration-response curves for the most active compounds tested against SKBR-3 breast cancer cells

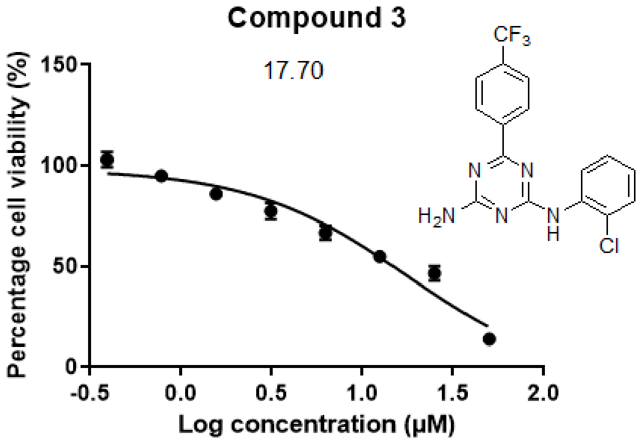

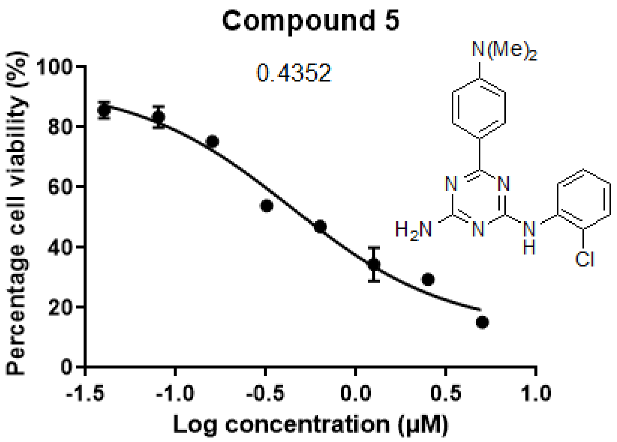

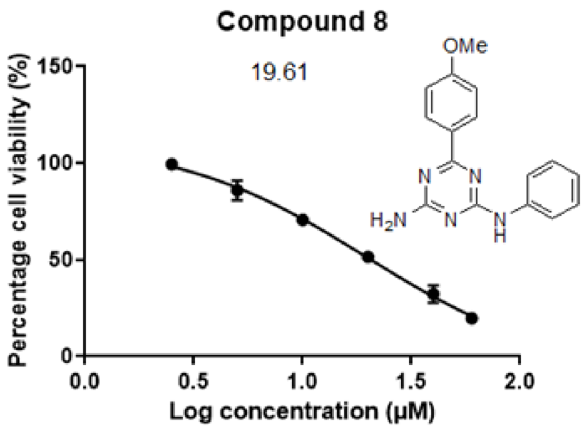

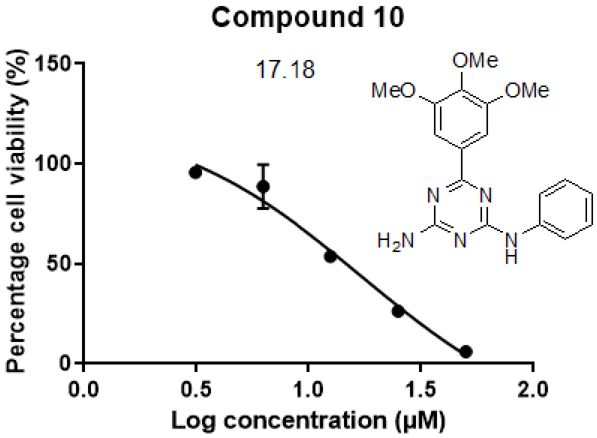

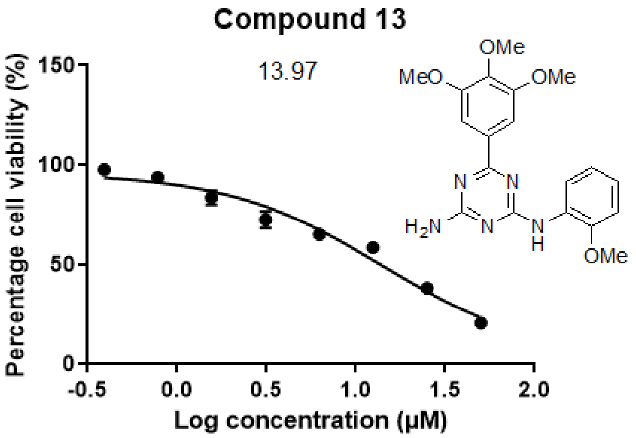

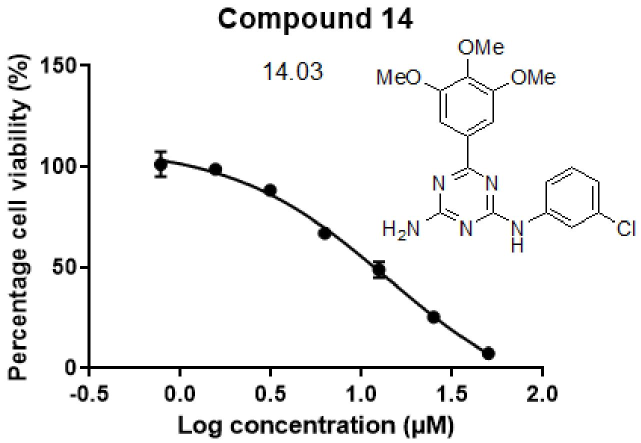

Compound 15

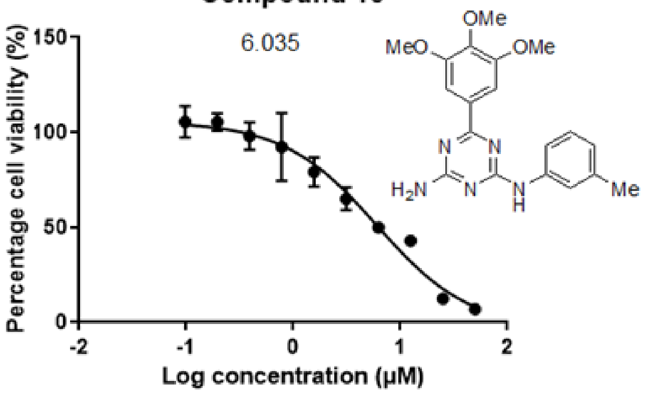

Compound 16

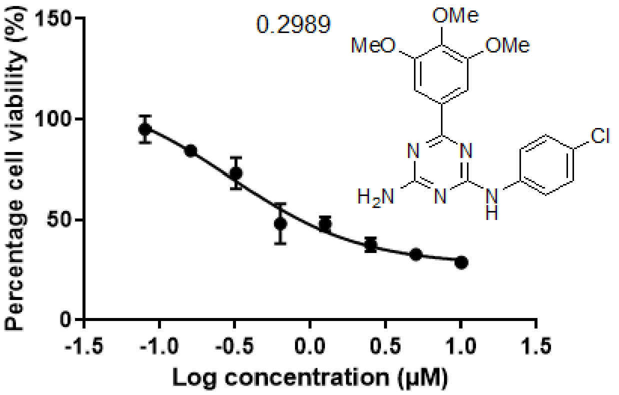

Compound 17

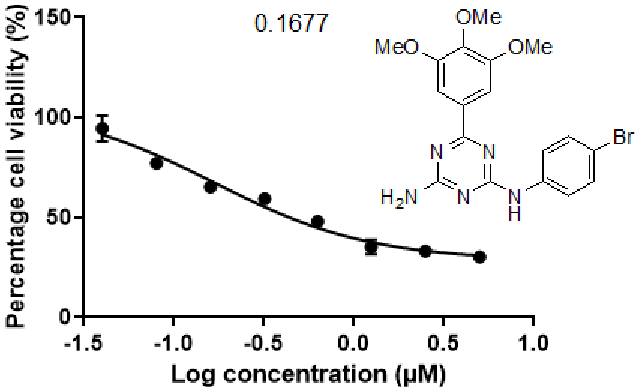

Compound 18

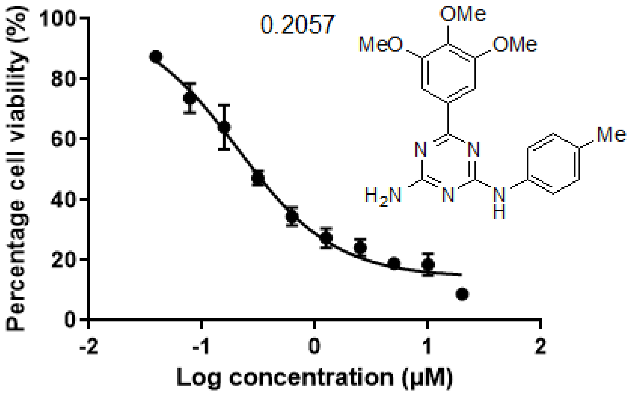

Compound 19

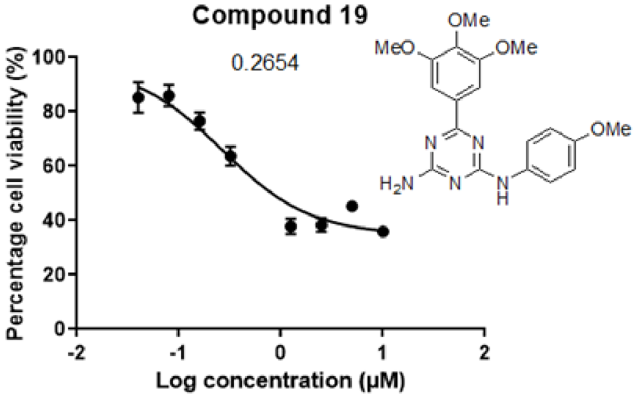

Compound 20

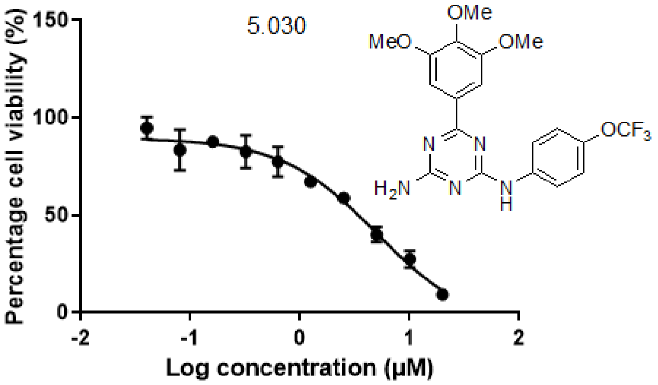

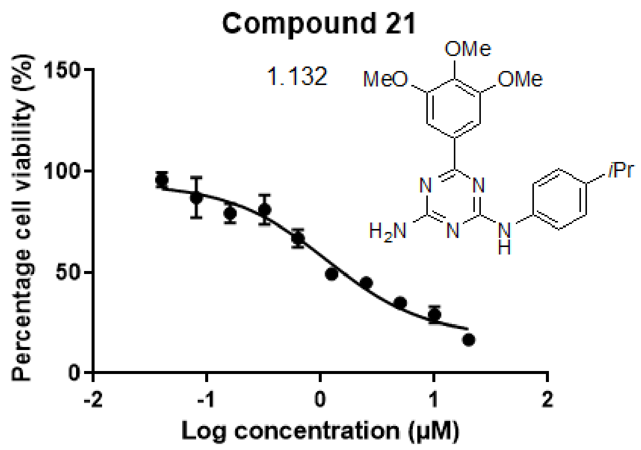
Compound 21
1.132
OMe
MeO
OMe
iPr
N
N
H_2N
N
N
H
Percentage cell viability (%)
150
100
50
0
-2
-1
0
1
2
Log concentration (μM)

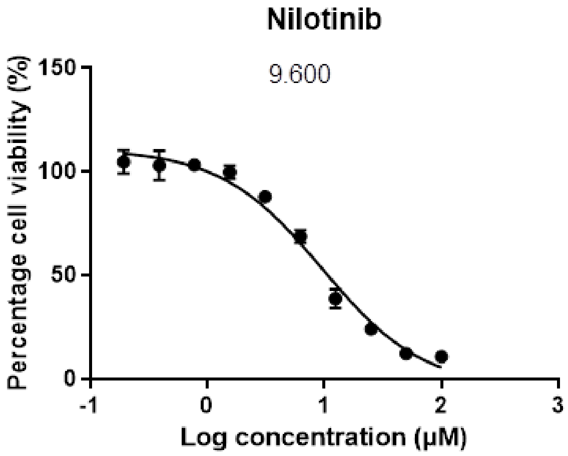
Nilotinib
9.600
Percentage cell viability (%)
150
100
50
0
-1
0
1
2
3
Log concentration (μM)

Concentration-response curves for the most active compounds tested against MCF-7 breast cancer cells

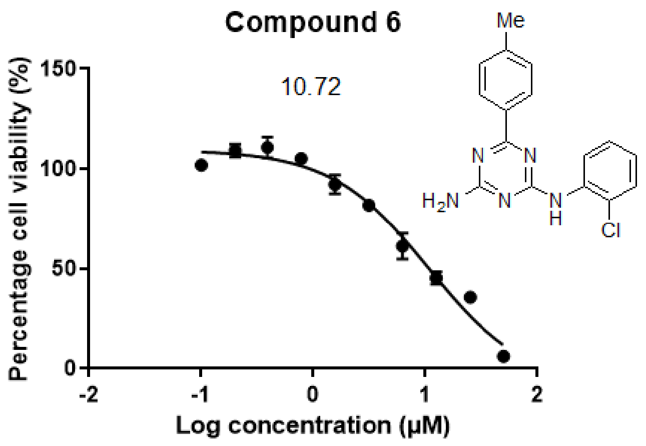

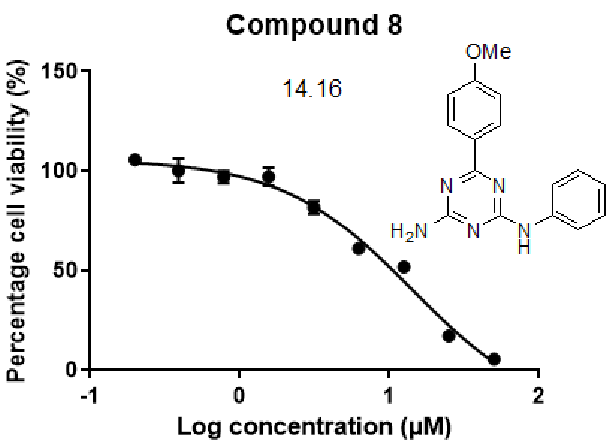

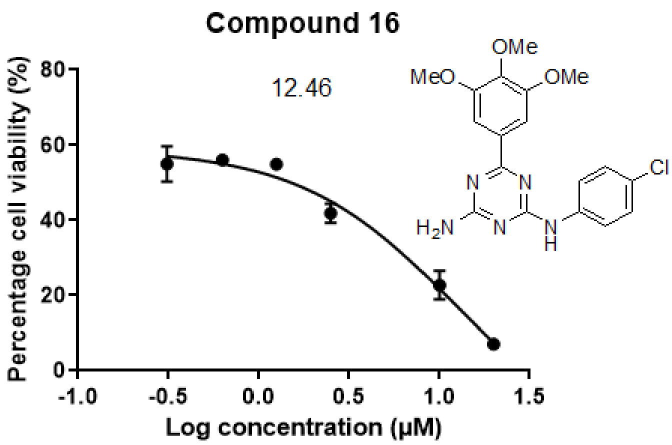

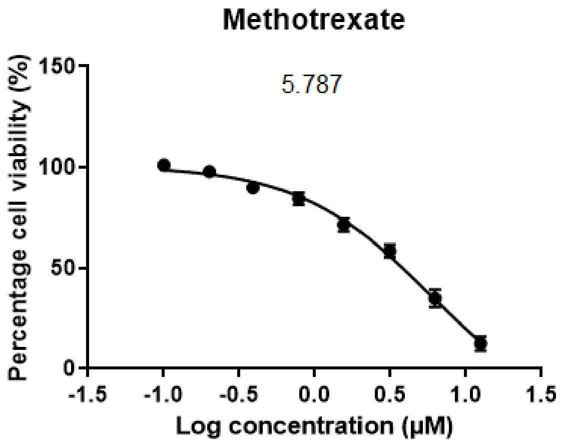

Time-dependent concentration-response curves for compounds 16 and 18 tested against MDA-MB231 breast cancer cells

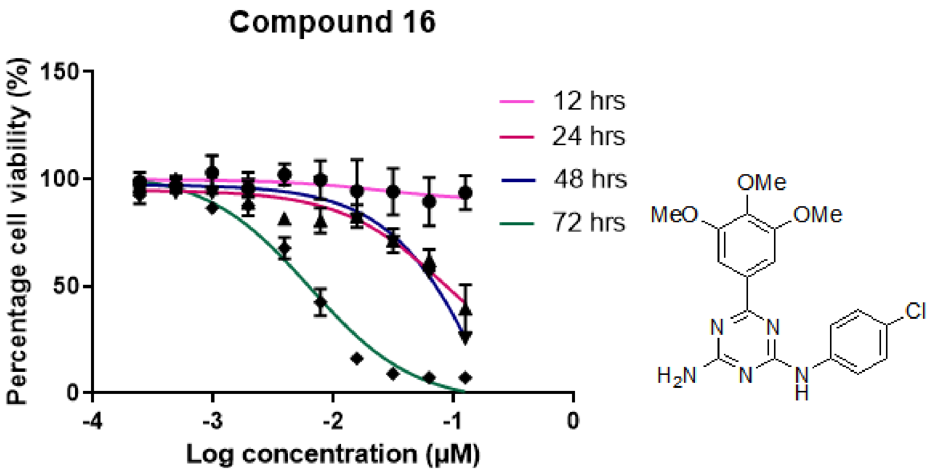

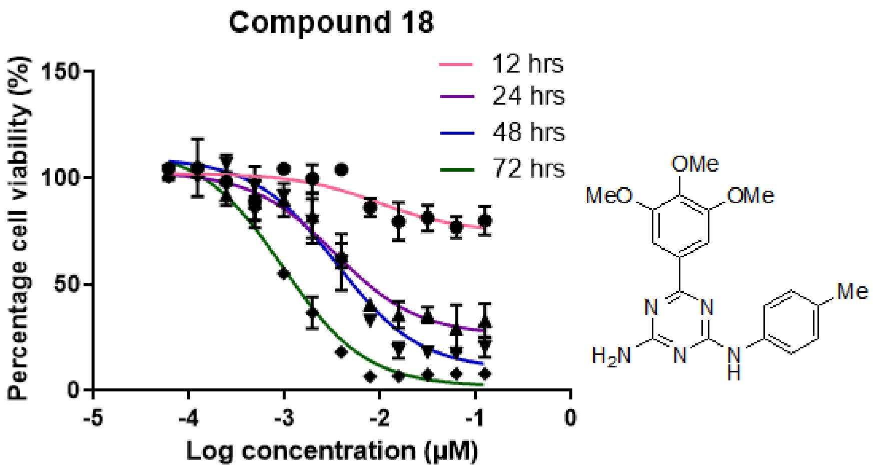

Predicted ADME properties of compounds 1-21

Table S1(a). Properties calculated / predicted by QikProp for compounds **1-7**

	Compounds						
**Property / Descriptor**	**1**	**2**	**3**	**4**	**5**	**6**	**7**
#stars	0	0	1	3	1	0	0
#rotor	5	4	4	5	5	4	4
CNS	-1	-1	-1	-1	-1	-1	-1
mol_MW	311.318	311.773	365.745	381.744	340.814	311.773	311.773
dipole	3.52	4.139	5.015	6.86	3.999	2.649	5.011
SASA	663.942	659.67	642.845	696.372	709.92	664.899	668.187
FOSA	88.845	87.813	22.596	2.005	153.168	87.856	87.857
FISA	112.503	116.153	108.718	115.201	109.667	108.614	116.165
PISA	420.217	384.054	357.419	384.981	381.353	402.696	392.521
WPSA	42.377	71.65	154.112	194.184	65.732	65.732	71.644
volume	1071.642	1076.618	1054.303	1114.602	1163.85	1074.633	1078.449
donorHB	3	3	3	3	3	3	3
accptHB	4.75	4	4	4	5	4	4
dip^2/V	0.011561	0.015911	0.023854	0.042222	0.013738	0.006528	0.023283
ACxDN^.5/SA	0.012392	0.010503	0.010777	0.009949	0.012199	0.01042	0.010369
glob	0.762784	0.770098	0.779296	0.74657	0.753738	0.763102	0.761144
QPpolrz	37.536	38.032	36.884	38.918	40.855	38.13	38.186
QPlogPC16	12.329	12.918	11.82	12.583	13.76	12.947	13.014
QPlogPoct	19.513	19.512	19.584	20.655	20.85	19.351	19.643
QPlogPw	12.925	12.186	11.942	12.096	13.007	12.272	12.264
QPlogPo/w	3.498	3.856	4.058	4.555	4.033	3.891	3.872
QPlogS	-5.716	-6.13	-6.198	-7.141	-6.539	-6.195	-6.278
CIQPlogS	-4.59	-4.844	-5.91	-6.23	-5.076	-4.844	-4.844
QPlogHERG	-7.374	-7.084	-6.808	-7.469	-7.335	-7.286	-7.269
QPPCaco	849.238	784.193	922.413	800.653	903.506	924.508	783.98
IP(eV)	8.288	8.467	8.728	8.652	8.115	8.546	8.546
EA(eV)	0.659	0.674	3.22	0.846	0.349	0.549	0.567
#metab	2	1	1	1	2	2	1
QPlogKhsa	0.229	0.405	0.352	0.448	0.421	0.395	0.404
HumanOralAbsorption	3	3	3	1	1	3	1
PercentHumanOralAbsorption	100	100	100	100	100	100	100
SAfluorine	42.377	0	88.37	122.533	0	0	0
SAamideO	0	0	0	0	0	0	0
PSA	73.984	66.209	64.771	74.891	67.919	64.702	66.211
#NandO	6	5	5	6	6	5	5
RuleOfFive	0	0	0	0	0	0	0
#ringatoms	18	18	18	18	18	18	18
#in56	18	18	18	18	18	18	18
#noncon	0	0	0	0	0	0	0
#nonHatm	23	22	25	26	24	22	22
RuleOfThree	1	1	1	1	1	1	1

Table S1(b). Properties calculated / predicted by QikProp for compounds **8-14**

	Compounds						
**Property / Descriptor**	**8**	**9**	**10**	**11**	**12**	**13**	**14**
#stars	0	0	1	1	2	1	2
#rotor	5	5	7	7	7	8	7
CNS	-2	-2	-2	-2	-2	-2	-2
mol_MW	293.327	307.354	353.38	371.37	387.825	383.406	387.825
dipole	4.364	3.967	5.125	4.618	4.676	3.498	6.725
SASA	644.114	666.657	735.081	742.371	755.895	766.443	759.212
FOSA	88.878	176.695	258.471	258.445	258.533	347.289	258.535
FISA	115.866	115.959	113.811	110.489	106.442	110.196	113.995
PISA	439.37	374.003	362.799	331.748	325.172	308.958	315.079
WPSA	0	0	0	41.689	65.749	0	71.603
volume	1043.191	1099.821	1194.192	1208.377	1234.265	1263.357	1238.065
donorHB	3	3	3	3	3	3	3
accptHB	4.75	4.75	6.25	6.25	6.25	7	6.25
dip^2/V	0.018256	0.014308	0.021993	0.01765	0.017719	0.009684	0.036532
ACxDN^.5/SA	0.012773	0.012341	0.014727	0.014582	0.014321	0.015819	0.014259
glob	0.772286	0.772937	0.740536	0.739058	0.736166	0.737399	0.734454
QPpolrz	36.58	38.222	40.608	40.879	41.852	42.219	41.908
QPlogPC16	12.45	12.575	13.569	13.212	14.156	13.898	14.229
QPlogPoct	19.145	19.571	21.089	21.259	21.678	21.744	21.997
QPlogPw	13.016	12.626	13.736	13.534	13.503	13.939	13.496
QPlogPo/w	3.167	3.44	3.433	3.665	3.934	3.337	3.914
QPlogS	-5.194	-5.578	-6.002	-6.308	-6.643	-6.15	-6.725
CIQPlogS	-4.248	-4.514	-4.891	-5.237	-5.556	-5.205	-5.556
QPlogHERG	-7.312	-6.99	-7.461	-7.336	-7.352	-7.255	-7.331
QPPCaco	789.122	787.511	825.332	887.434	969.421	893.131	822.034
IP(eV)	8.492	8.399	8.468	8.579	8.528	8.198	8.625
EA(eV)	0.496	0.486	0.572	0.634	0.625	0.5	0.646
#metab	2	2	4	4	4	5	4
QPlogKhsa	0.163	0.309	0.2	0.235	0.299	0.205	0.307
HumanOralAbsorption	3	3	3	1	1	3	1
PercentHumanOralAbsorption	100	100	100	100	100	100	100
SAfluorine	0	0	0	41.689	0	0	0
SAamideO	0	0	0	0	0	0	0
PSA	75.304	75.314	85.119	84.546	83.623	92.902	85.146
#NandO	6	6	8	8	8	9	8
RuleOfFive	0	0	0	0	0	0	0
#ringatoms	18	18	18	18	18	18	18
#in56	18	18	18	18	18	18	18
#noncon	0	0	0	0	0	0	0
#nonHatm	22	23	26	27	27	28	27
RuleOfThree	0	0	1	1	1	1	1

Table S1(c). Properties calculated / predicted by QikProp for compounds **15-21**

	Compounds						
**Property / Descriptor**	**15**	**16**	**17**	**18**	**19**	**20**	**21**
#stars	2	2	2	1	0	2	2
#rotor	7	7	7	7	8	8	8
CNS	-2	-2	-2	-2	-2	-2	-2
mol_MW	367.407	387.825	432.276	367.407	383.406	437.378	395.46
dipole	4.992	7.55	7.209	4.732	5.887	8.48	4.615
SASA	766.568	759.198	764.151	758.052	750.265	803.458	800.441
FOSA	346.307	258.528	258.531	346.327	347.388	260.791	422.065
FISA	113.872	113.995	114.023	113.955	114.03	115.09	114.194
PISA	306.388	315.035	314.274	297.77	288.846	306.026	264.182
WPSA	0	71.64	77.322	0	0	121.551	0
volume	1253.024	1238.006	1246.71	1250.965	1255.543	1349.821	1348.757
donorHB	3	3	3	3	3	3	3
accptHB	6.25	6.25	6.25	6.25	7	6.25	6.25
dip^2/V	0.019885	0.046045	0.041685	0.017901	0.027605	0.053274	0.015789
ACxDN^.5/SA	0.014122	0.014259	0.014167	0.01428	0.01616	0.013473	0.013524
glob	0.733253	0.734444	0.733099	0.740678	0.750191	0.735167	0.73755
QPpolrz	42.423	41.905	42.246	42.259	41.715	45.652	45.209
QPlogPC16	13.825	14.229	14.363	13.698	13.629	14.019	14.477
QPlogPoct	21.575	22.127	22.208	21.515	21.863	23.765	22.433
QPlogPw	13.429	13.496	13.505	13.35	13.789	13.345	13.005
QPlogPo/w	3.723	3.914	3.987	3.706	3.443	4.781	4.27
QPlogS	-6.538	-6.725	-6.834	-6.393	-5.878	-7.54	-6.958
CIQPlogS	-5.16	-5.556	-6.429	-5.16	-5.205	-6.53	-5.705
QPlogHERG	-7.309	-7.331	-7.349	-7.148	-6.952	-7.277	-7.039
QPPCaco	824.23	822.033	821.517	822.742	821.392	802.602	818.458
IP(eV)	8.442	8.52	8.624	8.372	8.302	8.661	8.357
EA(eV)	0.561	0.642	0.655	0.559	0.594	0.684	0.554
#metab	5	3	3	4	4	4	4
QPlogKhsa	0.344	0.307	0.328	0.345	0.197	0.555	0.554
HumanOralAbsorption	1	1	1	1	3	1	1
PercentHumanOralAbsorption	100	100	100	100	100	100	100
SAfluorine	0	0	0	0	0	121.551	0
SAamideO	0	0	0	0	0	0	0
PSA	85.128	85.141	85.146	85.136	94.304	94.193	85.163
#NandO	8	8	8	8	9	9	8
RuleOfFive	0	0	0	0	0	0	0
#ringatoms	18	18	18	18	18	18	18
#in56	18	18	18	18	18	18	18
#noncon	0	0	0	0	0	0	0
#nonHatm	27	27	27	27	28	31	29
RuleOfThree	1	1	1	1	1	1	1

QikProp descriptors and properties

#stars = Number of property or descriptor values that fall outside the 95% range of similar values for known drugs.

#rotor = Number of non-trivial (not CX3), non-hindered (not alkene, amide, small ring) rotatable bonds.

CNS = Predicted central nervous system activity on a –2 (inactive) to +2 (active) scale.

Dipole = Computed dipole moment of the molecule.

SASA = Total solvent accessible surface area (SASA) in square angstroms using a probe with a 1.4 Å radius.

FOSA = Hydrophobic component of the SASA (saturated carbon and attached hydrogen).

FISA = Hydrophilic component of the SASA (SASA on N, O, and H on heteroatoms).

PISA = π (carbon and attached hydrogen) component of the SASA.

WPSA = Weakly polar component of the SASA (halogens, P, and S).

Volume = Total solvent-accessible volume in cubic angstroms using a probe with a 1.4 Å radius. 500.0 – 2000.0

donorHB = Estimated number of hydrogen bonds that would be donated by the solute to water molecules in an aqueous solution.

accptHB = Estimated number of hydrogen bonds that would be accepted by the solute from water molecules.

dip^2/V = Square of the dipole moment divided by the molecular volume. This is the key term in the Kirkwood-Onsager equation for the free energy of solvation of a dipole with volume V.

ACxDN^.5/SA = Index of cohesive interaction in solids, $(accptHB)\ (donorHB)^{1/2})\,/\,(SA)$

glob = Globularity descriptor, $(4\pi r^2)\,/\,(SASA)$, r is the radius of a sphere with a volume equal to the molecular volume. Globularity is 1.0 for a spherical molecule.

QPpolrz = Predicted polarizability in cubic angstroms.

QPlogPC16 = Predicted hexadecane/gas partition coefficient.

QPlogPoct = Predicted octanol/gas partition coefficient.

QPlogPw = Predicted water/gas partition coefficient.

QPlogPo/w = Predicted octanol/water partition coefficient.

QPlogS = Predicted aqueous solubility, log S. S in mol dm–3 is the concentration of the solute in a saturated solution that is in equilibrium with the crystalline solid.

CIQPlogS = Conformation-independent predicted aqueous solubility, log S. S in mol dm–3 is the concentration of the solute in a saturated solution that is in equilibrium with the crystalline solid.

QPlogHERG = Predicted IC50 value for blockage of HERG K+ channels.

QPPCaco = Predicted apparent Caco-2 cell permeability in nm/sec. Caco-2 cells are a model for the gut blood barrier.

IP(ev) = PM3 calculated ionization potential.

EA(eV) = PM3 calculated electron affinity.

#metab = Number of likely metabolic reactions.

QPlogKhsa = Prediction of binding to human serum albumin.
HumanOralAbsorption = Predicted qualitative human oral absorption:
PercentHuman-OralAbsorption = Predicted human oral absorption on 0 to 100% scale. The prediction is based on a quantitative multiple linear regression model. This property usually correlates well with HumanOral-Absorption, as both measure the same property.
SAFluorine = Solvent-accessible surface area of fluorine atoms.
SAamideO = Solvent-accessible surface area of amide oxygen atoms.
PSA = Van der Waals surface area of polar nitrogen and oxygen atoms.
#NandO = Number of nitrogen and oxygen atoms.
RuleOfFive = Number of violations of Lipinski's rule of five. The rules are: mol_MW < 500, QPlogPo/w < 5, donorHB ≤ 5, accptHB ≤ 10. Compunds that satisfy these rules are considered drug-like.
RuleOfThree = Number of violations of Jorgensen's rule of three. The three rules are: QPlogS > -5.7, QP PCaco > 22 nm/s, # Primary Metabolites < 7. Compounds with fewer (and preferably no) violations of these rules are more likely to be orally available.
#ringatoms = Number of atoms in rings.
#in56 = Number of atoms in 5- or 6-membered rings.
#noncon = Number of ring atoms not able to form conjugated aromatic systems (e.g. sp3 C).
#nonHatm = Number of heavy atoms (nonhydrogen atoms).
